# Scaling and self-similarity in the formation of the embryonic epigenome

**DOI:** 10.1038/s41567-026-03263-x

**Published:** 2026-04-29

**Authors:** Fabrizio Olmeda, Tim Lohoff, Ioannis Kafetzopoulos, Stephen J. Clark, Laura Benson, Fatima Santos, Felix Krueger, Simon Walker, Wolf Reik, Steffen Rulands

**Affiliations:** 1 Max Planck Institute for the Physics of Complex Systems, Dresden, Germany; 2 Institute of Science and Technology Austria, Klosterneuburg, Austria; 3 Epigenetics Programme, The Babraham Institute, Cambridge, UK; 4 Wellcome-MRC Cambridge Stem Cell Institute, Jeffrey Cheah Biomedical Centre, University of Cambridge, Cambridge, UK; 5 Altos Labs, Cambridge Institute of Science, Cambridge, UK; 6 Bioinformatics Group, The Babraham Institute, Cambridge, UK; 7 Imaging Facility, The Babraham Institute, Cambridge, UK; 8 Department of Physiology, Development and Neuroscience, University of Cambridge, Cambridge, UK; 9 Arnold Sommerfeld Center for Theoretical Physics and Center for NanoScience, Department of Physics, Ludwig-Maximilians-Universität, Munich, Germany

## Abstract

The development of complex tissues relies on the precise assignment of cell identity. At the molecular scale, this process depends on the deposition of epigenetic modifications—such as methylation—that are regulated by complex biochemical networks and occur at specific regions on the DNA and chromatin. Here we show that despite the complexity of epigenetic regulation, dynamical scaling and self-similarity of DNA methylation marks emerge in embryonic development. Drawing on single-cell multi-omics experiments, super-resolution microscopy and statistical physics, we demonstrate that these phenomena originate in dynamical feedback between DNA methylation and the formation of nanoscale dynamic chromatin aggregates. These nanoscale processes lead to genome-wide increase in DNA methylation marks following a power law and self-similar correlation functions. Using this framework, we identify methylation patterns that precede gene expression changes in embryonic symmetry breaking. Our work identifies linear sequencing measurements as a laboratory to study mesoscopic biophysical processes in vivo.

The self-organization of cells into complex tissues during development relies on the tightly regulated behaviour of individual cells. This regulation is governed by molecular programs that include not only gene expression but also epigenetic modifications—chemical changes to DNA and histones, the proteins around which DNA is wrapped—as well as their spatial organization within the nucleus^1,2^ (Fig. 1a). Recent advances in genomics have enabled the characterization of intricate biochemical networks that orchestrate the spatial and temporal positioning of these epigenetic modifications. Among these, DNA methylation—an essential layer of epigenetic regulation—involves the addition of a methyl group to cytosine residues, primarily within CpG dinucleotides. This process has a pivotal role in development, ageing and disease^3,4^.

In early mammalian development, the zygote comprises pluripotent cells capable of differentiating into all adult cell types. During this phase, a genome-wide erasure of DNA methylation occurs, effectively resetting the epigenetic landscape^5^. As development progresses and cells exit pluripotency, the genes *Dnmt3a* and *Dnmt3b* become upregulated. These gene produce de novo methyltransferase enzymes that rapidly re-establish methylation patterns across the genome. This results in methylation of over 80% of CpGs^6,7^.

Despite this well-documented transition, the spatiotemporal dynamics governing the establishment of the embryonic methylome remain poorly understood. Here we combine single-cell DNA methylation experiments with a novel theoretical framework that applies methods from non-equilibrium statistical physics^8^ to epigenomic processes. Our analysis reveals that feedback between one-dimensional biochemical modifications along the genome and three-dimensional chromatin organization gives rise to emergent scaling laws during the establishment of embryonic methylation patterns. Specifically, we identify self-similar time evolution in average DNA methylation levels, characterized by a power law with an exponent of 5/2, invariant across genomic regions. Furthermore, our theory predicts spatial two-point correlation functions that are in excellent agreement with in vitro experimental data. The emergence of scaling suggests that physical constraints—emerging from the chromatin architecture and enzyme dynamics—have a central role in the establishment of the embryonic epigenome. Finally, using these results, we identify genomic regions that dynamically change before gene silencing events during the earliest symmetry-breaking transitions in the embryo.

## Results

**Fig. 1:**
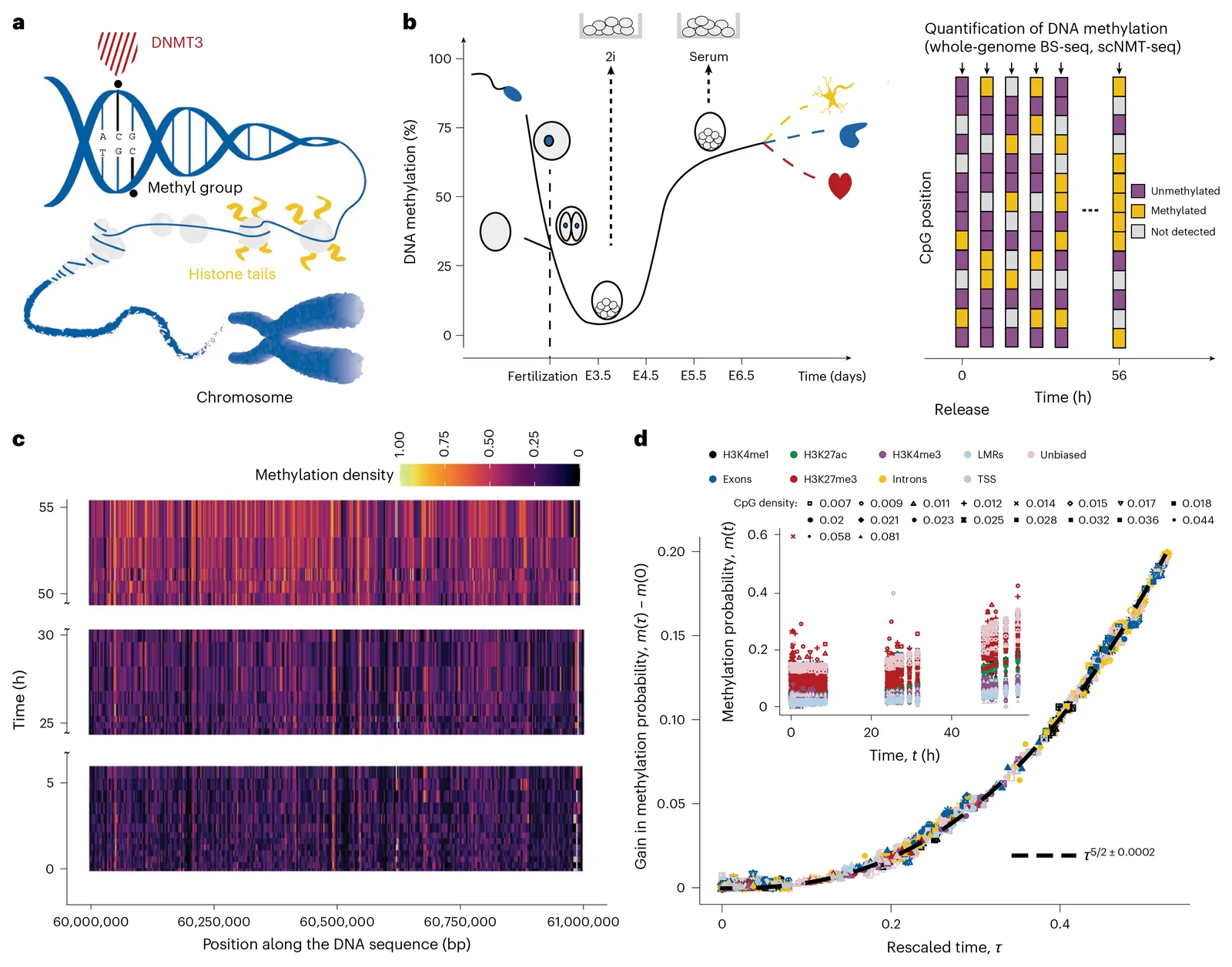
Scaling and self-similarity of de novo DNA methylation. **a**, Schematic showing epigenetic processes involved in regulating cell fate. **b**, Schematic showing the time evolution of global DNA methylation levels during early mouse development (left) and the quantification of DNA methylation in the 2i release experiment. In single-cell BS-seq, a given CpG site can be measured as methylated, unmethylated or undetected. **c**, Heat map showing DNA methylation levels across an exemplary genomic domain over time. **d**, Inset: average probability that a CpG site is methylated increases at different rates in genomic regions with different functional annotation (colour; Extended Data Table 1) and CpG density (shape). H3K4me1, histone H3 Lys4 monomethylation; H3K4me3, histone H3 Lys4 trimethylation; H3K27me3, histone H3 Lys27 monomethylation; H3K27ac, histone H3 Lys27 acetylation; LMR, lowly methylated regions; TSS, transcription start sites; Unbiased, tiling of the genome into windows of equal coverage. The main plot shows the curves that were parameterized as *m*(*t*) = *m*(0) + *bt*^*c*^; *τ* = *t*/*b* is shown on the *x* axis and *m*(*τ*) − *m*(0) on the *y* axis (see ‘Quantification of DNA methylation dynamics’ section). The gain in average DNA methylation follows a single power law with an exponent of 5/2 (dashed line; Methods). The exponent is robust with respect to log transformation and its uncertainty denotes 95% confidence intervals.

To systematically understand the dynamics of DNA methylation marks, we used an experimental model system that has been shown to recapitulate the epigenetic and transcriptional changes occurring in vivo before cells are primed for differentiation between 3.5 and 5.5 days after fertilization in the mouse embryo^9–12^. In this model system, mouse embryonic stem cells (mESCs) were cultured long term in 2i culture conditions (see ‘2i release cell culture’ section), in which DNA methylation is globally reduced. Cells were then released into serum conditions (Fig. 1b; see ‘2i release whole-genome BS-seq’ section for details of the cell culture and a discussion of similarity to the mouse embryo), where the methyltransferase enzymes DNMT3a and DNMT3b that can convert unmethylated cytosines into methylated cytosines are upregulated^13^. After release into serum conditions, we performed two complementary sets of experiments (Fig. 1b). (1) A whole-genome bisulfite-sequencing (BS-seq) time course of 31 time points over a period of 56 h, giving access to a high-coverage quantification of DNA methylation with high temporal resolution. (2) A single-cell NMT-sequencing (scNMT-seq) experiment of 288 cells with a lower temporal resolution (0 h, 24 h and 48 h), providing simultaneous information on the genomic distribution of DNA methylation and accessibility as well as on the transcriptome in single cells^14^ (see ‘2i release scNMT-seq’ section). Details on the processing of sequencing data are given in the ‘Processing of whole-genome BS-seq data’ section.

**Extended Data Fig. 1:**
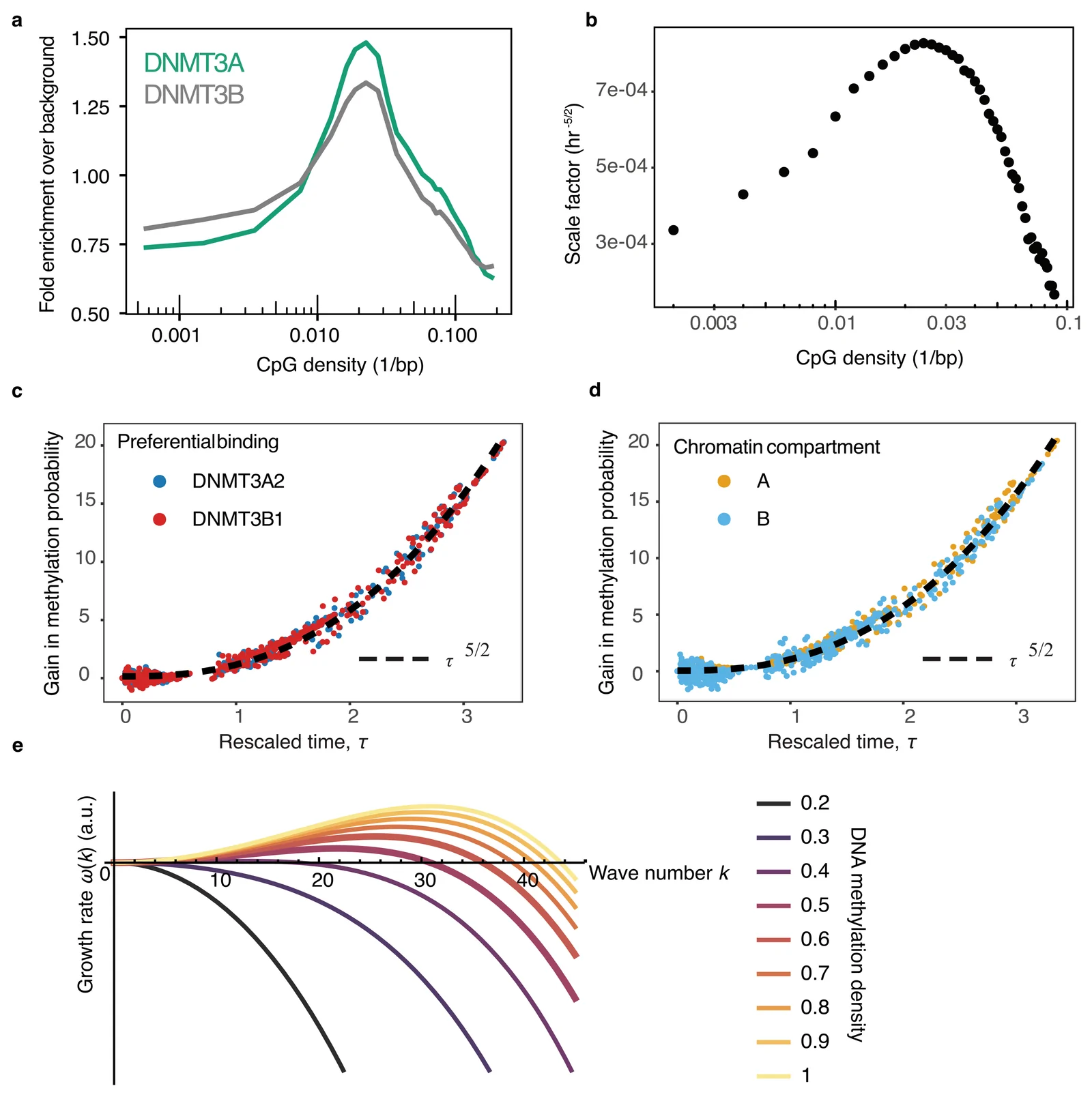
Enzyme-independent scaling and self-similarity. (**a**) Fold enrichment of DNMT3A/B Chip-seq signals from^15^ over a background signal as a function of CpG densitiy for DNMT3A and DNMT3B. (**b**) Scale factor of the scaling form in Fig. 1d as a function of the CpG density. The same genomic windows were used as for the unbiased probes with constant coverage in Fig. 1d. (**c**) Scaling form of average DNA methylation as in Fig. 1d. Here, we partitioned the genome into genomic regions based on the preferential binding of DNMT3A2 or DNMT3B1 based on their respective Chip-seq signals from^15^. (**d**) Scaling form of average DNA methylation as in Fig. 1d. Here, we subdivided the genome into large heterochromatin (A) and euchromatin regions (B). Annotations were taken from [59]. The dashed line in (**c**) and (**d**) is identical to the dashed line in Fig. 1d. (**e**) Linear stability analysis considers the rate of growth of small perturbations with a given wave number, k (spatial frequency). It shows that for sufficiently high levels of DNAme the equation presented in (1) leads to the growth of perturbations with a finite wave number, and therefore the formation of mesoscopic patterns in the methylation density.

We initially focused on the time evolution of DNA methylation levels during de novo DNA methylation in the BS-seq experiment (Fig. 1c). The placement of methylation marks is thought to be regulated by a number of different factors, including the density of CpGs, especially highly dense regions known as CpG islands, transcription and histone modifications^15^. As a reflection of the complexity of de novo DNA methylation, we found that functionally distinct genomic regions (such as promoters, gene bodies, CpG islands and so on), obtained from previously published Chip-seq data (Extended Data Table 1) acquired average DNA methylation levels at different rates (Fig. 1d, inset). However, rescaling time by a scale factor (see ‘Quantification of DNA methylation dynamics’ section) collapsed all curves onto a single scaling form. A comparison of the magnitude of the scale factor to DNMT3A/B binding locations obtained from previously published Chip-seq data (Extended Data Table 1 and Extended Data Fig. 1a,b) suggests that the scale factor mostly describes genomic variations in enzyme binding affinities. More importantly, the emergence of scaling itself suggests that there is a generic mechanism of how DNA methylation is established genome wide. In particular, the collapsed scaling curve almost perfectly follows a power law with a non-trivial exponent of 5/2 (Fig. 1d). This implies self-similarity in time such that the time evolution of average DNA methylation levels is scale invariant. Temporal scale invariance and scaling behaviour with a specific exponent of 5/2 are reminiscent of collective, self-organization processes^16^, suggesting that DNA methylation marks are established via a collective mechanism involving interacting DNMT3 enzyme molecules^17,18^. The shape of the scaling curve reflects a genome-wide, generic mechanism governing de novo DNA methylation. Unintuitively, the emergence of self-similar scaling and the specific shape of the scaling curve are independent of the the genomic context, even for regions that are regulated differently, such as active or repressive regions (Fig. 1d), binding regions of two distinct members of the DNMT3 family (Extended Data Fig. 1c), or compartments of dense or dilute chromatin (A and B compartments; Extended Data Fig. 1d).

### From sequence to physical space

**Fig. 2:**
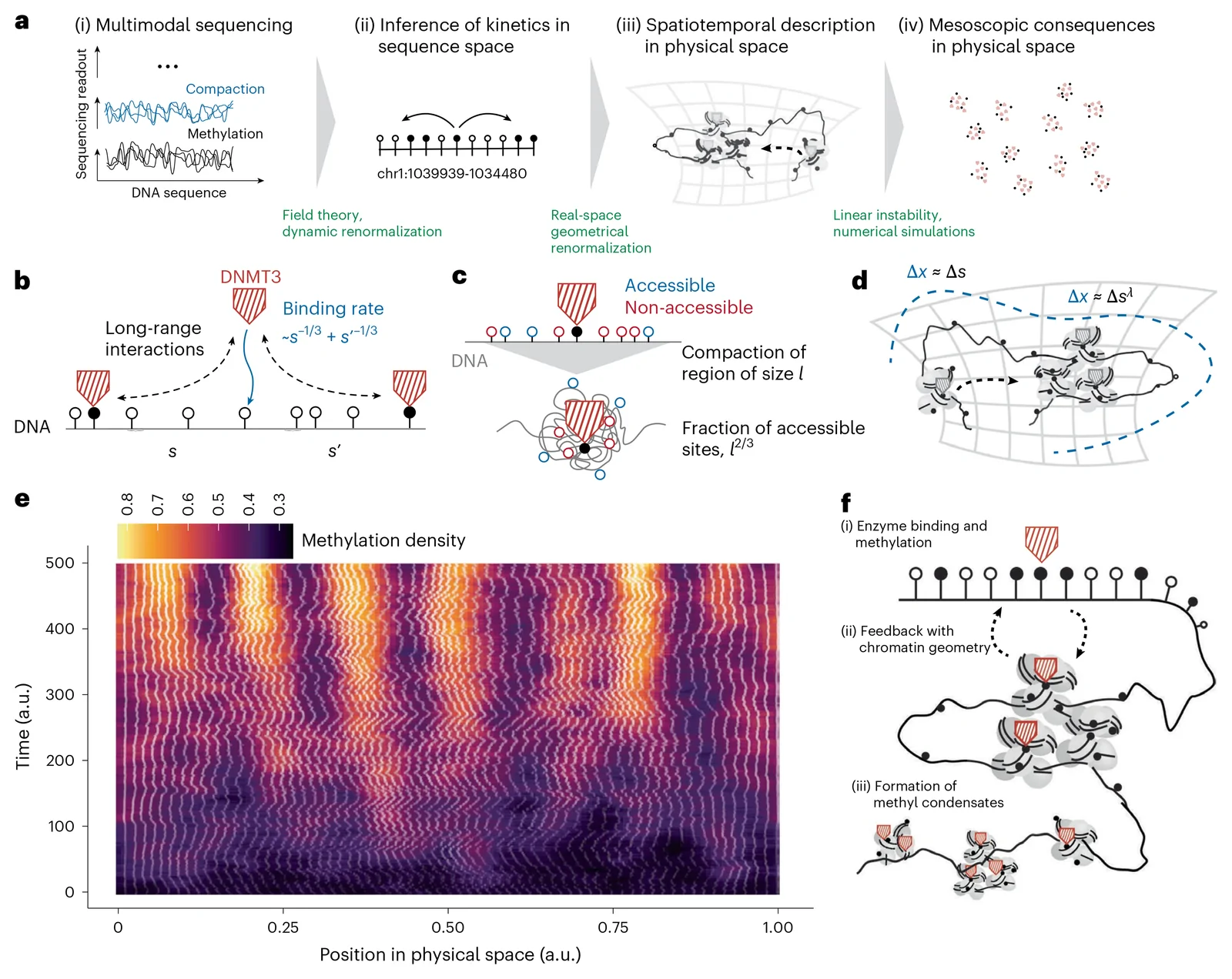
Inference from sequence to physical space and time. **a**, Summary of the theoretical approach. **b**, Schematic illustrating the inferred quantification of interactions between methylation events in sequence space. DNMT3A/B enzymes bind to unoccupied CpG sites with a rate that decreases with the distance to the nearest bound sites. **c**, Geometric interpretation of the inferred interactions. The binding rate along the one-dimensional DNA sequence is interpreted as feedback between de novo methylation and chromatin compaction in the three-dimensional space of the cell nucleus. **d**, Illustration of the transformation from sequence space to physical space. Binding and methylation induce a local compaction of chromatin, thereby creating a dynamical feedback loop between methylation and chromatin conformation. **e**, Simulation of the stochastic dynamics in equation (1) (Supplementary Information, section III). Colour denotes DNA methylation density and white lines denote the trajectories of fixed positions in sequence in physical space. Both time and physical space are dimensionless. **f**, Dynamics of DNA methylation in physical space gives rise to feedback between local chromatin condensates and the establishment of new DNA methylation marks.

Understanding the biophysical origin of self-similarity and scaling requires understanding how these collective processes come about in space and time and on the mesoscopic scale. To do this, we developed a theoretical framework that maps detailed measurements along the linear DNA sequence to mesoscopic processes in physical space (Fig. 2a and Supplementary Information, section I). Specifically, we begin by defining a minimal ansatz for general out-of-equilibrium stochastic enzyme kinetics incorporating (1) binding and unbinding of enzymes to the DNA and (2) chemical modifications of the DNA. Apart from these processes describing typical enzyme kinetics, this framework also incorporates (3) general and unknown interactions of enzymes along the DNA sequence. The time evolution of the probability, $P(\vec{D},\vec{m})$, of finding a given enzyme binding profile $\vec{D}$ and DNA methylation profile $\vec{m}$ at a given time *t* then follows a master equation that contains an as-yet-unknown interaction kernel *J*_*ij*_ quantifying how individual binding events at genomic positions *i* and *j* influence each other (Supplementary Information, section II). By calculating the first and second moments of $P(\vec{D},\vec{m})$ using a path-integral approach (Supplementary Information, sections II.1 and II.2 and ref. 19), we found that for interactions decaying as *J*_*ij*_ ∝ ∣*i* − *j*∣^−*λ*^ restricted to the closest bound enzymes (Fig. 2b), the first moment of DNA methylation increases according to ⟨*m*⟩ ∝ *t*^1+1/(1−*λ*)^. We, therefore, obtain *λ* = 1/3 by comparison with Fig. 1d (Supplementary Information, section II.3).

To understand the biophysical implication of the mathematical form of the interaction kernel, it is instructive to consider the ensuing total binding rate in the vicinity of size *l* of a bound site. This is obtained by summing over all contributions in this region. For linear DNA, this binding rate should increase linearly with the length of the region. In the present case, the integration of *J*_*ij*_ shows, however, that it scales as *l*^2/3^ (Fig. 2c and Supplementary Information, section II.3). The scaling with *l*^2/3^, therefore, implies that only a fraction of sites in the vicinity of a bound site is accessible for further binding. Indeed, *l*^2/3^ is the surface-to-volume ratio of three-dimensional objects or the fraction of base pairs that would be accessible if the DNA was compacted in a volume proportional to *l*. Therefore, the inferred interaction kernel has the biophysical interpretation of the compaction of the DNA around methylated sites and the preferential binding of DNMT3 to compacted regions (Fig. 1c). This is fully consistent with biochemical studies, showing that DNA methylation leads to attractive forces between tetra-nucleosomes in vitro^20^.

Having inferred the kinetics of de novo DNA methylation along the one-dimensional sequence of the DNA, we now infer how such dynamics translate to the yet-unknown three-dimensional conformational changes of the DNA. To derive a description in physical space, we take a purely statistical and geometrical approach that does not require detailed knowledge of the biophysical parameters governing chromatin on this scale (Supplementary Information, section III.1). In brief, for a given genomic position *i*, we consider the co-evolution of length elements in sequence space Δ*s*_*i*_ and small-length elements in physical space Δ*x*_*i*_ (Fig. 2d). The relation between both is encoded in a metric that is a function of the time derivative of the concentration field of bound DNMT3 enzymes. We define a physical space *x* as a projection of DNA along a one-dimensional curve that has the property that its total length remains constant^21^ (Fig. 2d). We achieved this by applying a real-space geometrical renormalization-group scheme in which concentrations at a discrete position *i* change in response to binding events by locally contracting the chromatin. By incorporating this scheme into the master equation (Supplementary Information, section III.2), we found that the density of DNMT3 enzymes along the projected one-dimensional physical space, *ϕ*(*x, t*), evolves in dimensionless form as

(1) $$\begin{array}{c} \partial_{t}\phi (x,t)=\phi {\left(x,t\right)}^{\lambda }+\phi {\left(x,t\right)}^{\lambda -3}\partial_{x}^{2}\phi (x,t)\\ -r\phi {\left(x,t\right)}^{\lambda }\partial_{x}^{2}\phi (x,t)+O\left(\partial_{x}^{4}\phi (x,t)\right) \end{array}$$

with *λ* = 1/3. The first term describes the local increase in the density of DNMT3 enzymes. The second and third terms describe diffusion and aggregation in physical space, respectively, whereas the higher-order terms prevent the formation of arbitrarily small structures. The dimensionless parameter *r* gives the relative strength of chromatin aggregation and dispersion. DNA methylation density is then obtained from equation (1) as ∂_*t*_*m*(*x*) = *kϕ*(*x*), where *k* is the methylation rate.

As the local concentration of DNMT3 enzymes, *ϕ*(*x, t*), reaches a threshold value, aggregation processes dominate. Specifically, linear stability analysis (Supplementary Information, section III.2 and Extended Data Fig. 1e) shows that the system undergoes a type-II instability if the local average value of the DNMT3 concentration reaches a threshold of *r*^−1/3^. Equation (1), therefore, describes the formation of highly methylated genomic domains that coincide with domains of dense chromatin. This behaviour resembles phase separation or spinodal decomposition processes^22,23^, describing systems in which two or more components—here methylated and non-methylated DNA—spatially separate. As observed in the case of phase-separating systems when chemical reactions are present in the system^24^, these domains do not coarsen over time.

This finding is supported by stochastic simulations (Fig. 2e and Supplementary Information, section VII). Because such clusters depend on the local DNA methylation level, their size is heterogeneous with a predicted typical size in the order of 5,000 bp (Supplementary Information, section III.3). With a typical extension of 5.5 nm for a nucleosome and a random packing fraction of 0.64, this gives a rough lower-bound estimate for a diameter of 40 nm (see ‘Numerical conversion between genomic and physical distances’ section). Heterogeneous structures of markedly similar size (roughly 12–40 nm) termed ‘clutches’ have been described in super-resolution imaging experiments^25–27^ and are correlated with histone tail methylation and acetylation^27^ and the presence of the H1 linker nucleosome^25,28^.

Together, although the model describes processes on the nanometre or kilobase scale, these processes determine the rate of increase in the global average DNA methylation level on the genome scale. Our theory explains why genomic regions that are regulated in different ways, such as active and repressive genomic regions, follow the same scaling form as that shown in Fig. 1d. A renormalization-group argument (Table 1 and Supplementary Information, section II.4) shows that the feedback between de novo methylation and local chromatin compaction statistically dominates over other processes, such as enzyme processivity^27^, cooperativity^18^ or DNMT3 oligomerization^6^. This is analogous to universality classes in the theory of critical phenomena. Taken together, our approach predicts that the origin of self-similar scaling during early embryonic development is an interplay between chemical modifications of the DNA and geometrical changes thereof via the formation of chromatin aggregates (Fig. 2f).

### Validation of model predictions

**Extended Data Fig. 2:**
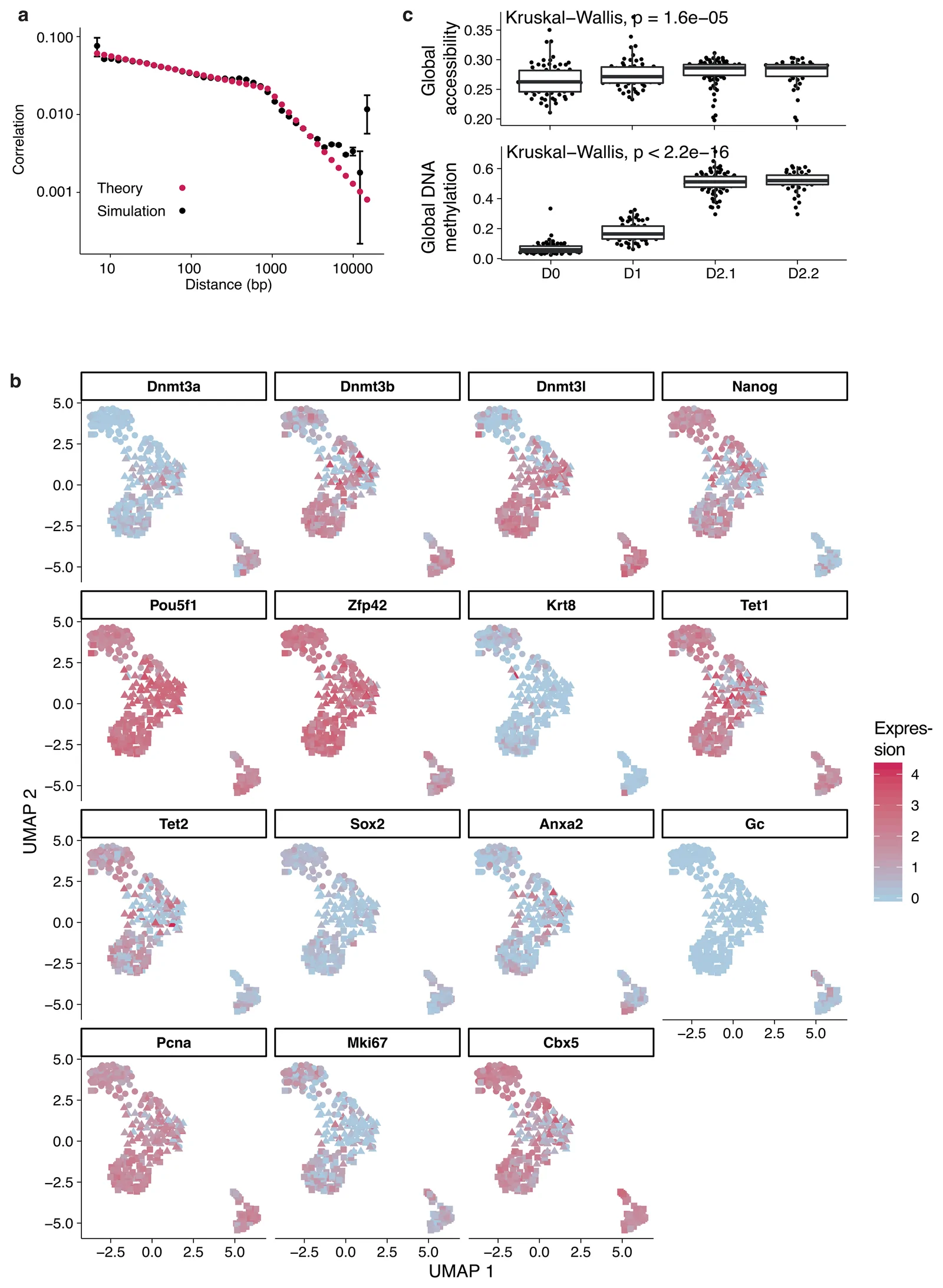
Analysis of scNMT-seq time course after 2i release. (**a**) Comparison of our analytical prediction with a stochastic simulation performed on a virtual DNA statistically resembling the distribution of CpGs in the mouse genome. Error bars indicate the standard deviation computed over 100 stochastic realizations of the master equation and over all pairs of base pairs separated by the same genomic distance. The centers of the dots represent averages over all realisations. (**b**) Average accessibility (top) and DNA methylation (bottom) over the time course of the 2i release scNMT-seq experiment. Each dot represents a cell, lower and upper hinges correspond to the first and third quartile and whiskers extend to 1.5 times the inter quartile range. The horizontal line is the median. p-values were obtained using the Kruskal-Wallis test. The p-value in the lower panel is smaller than the machine epsilon. n = 64 cells for D0, n = 62 for D1, n = 38 for D2.1, n = 44 for D2.2. (**c**) Log-normalised expression of a set of genes shown on a UMAP projection.

**Fig. 3:**
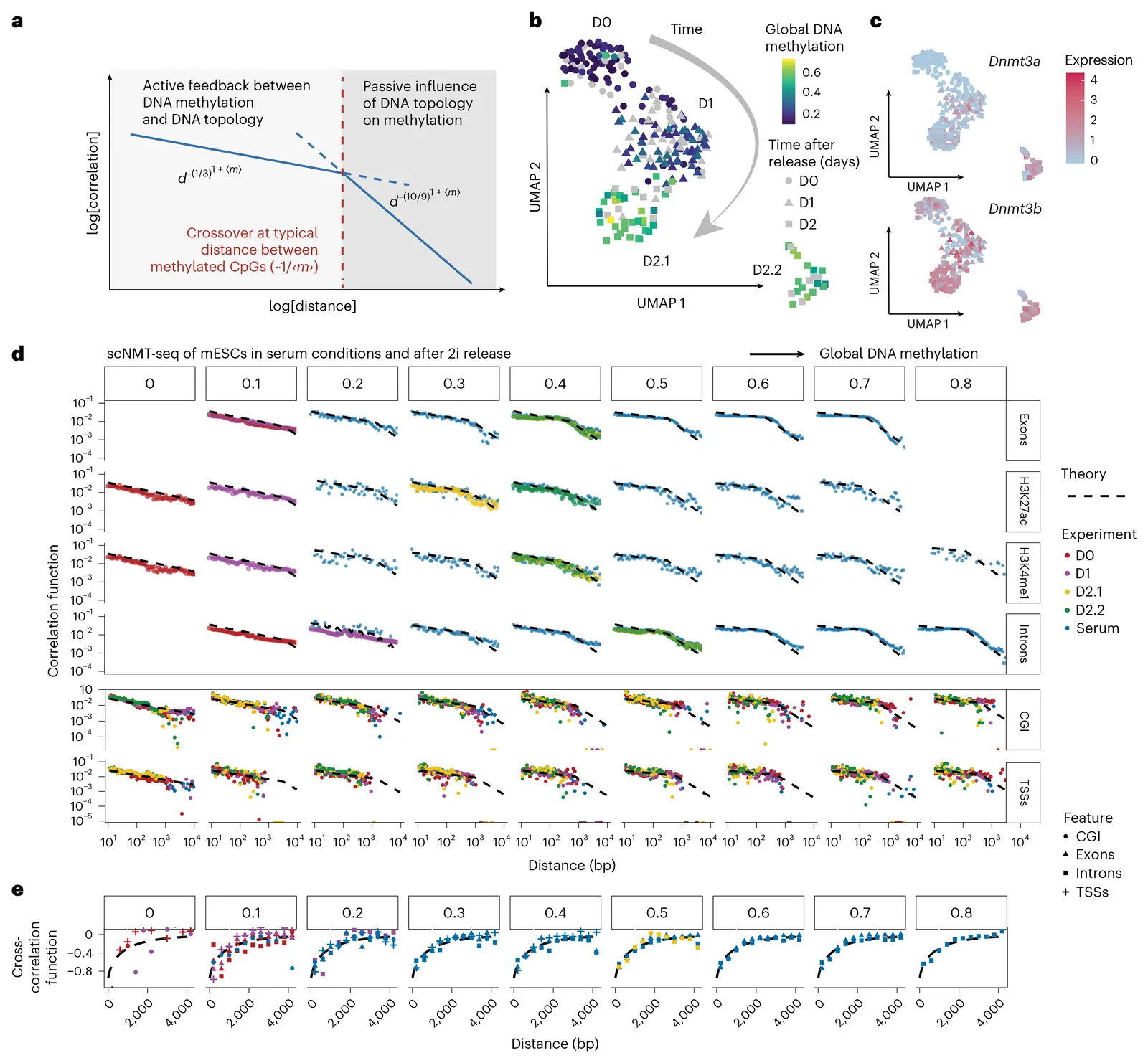
Genomic correlation functions. **a**, Graphical summary of the processes determining the shape of correlations in DNA methylation, as previously described^19^. **b**, Dimensionality-reduced representation (UMAP) of the scNMT-seq experiment of mESCs in the 2i release experiment. UMAP coordinates of cells (points) were calculated from transcriptomes; colours denote the average DNA methylation in each cell. A distinct cluster of cells present on day 2 (D2.2) showed signs of cellular stress (Methods), but did not show any differences in DNA methylation patterns. **c**, Same UMAP coordinates overlaid with the expression levels of *Dnmt3a*/*b* genes. **d**, Prediction of correlation functions of DNA methylation in cells with different levels of global DNA methylation and empirical correlation functions from scNMT-seq experiments after 2i release and in serum conditions. We computed the correlation functions for several genomic annotations (rows; see also Extended Data Table 1). CGI, CpG islands, that is, dense genomic clusters of CpGs. Deviations between theory and experiment stem from statistical uncertainty, analytical approximations (Extended Data Fig. 2a) and potentially from systematic variations in sequence context. **e**, Prediction of the cross-correlation between DNA methylation and accessibility and empirical cross-correlations, normalized by the prefactor estimated in Extended Data Fig. 3b, from scNMT-seq experiments after 2i release and in serum conditions. Shapes denote genomic annotations (Extended Data Table 1).

To experimentally verify these findings, we now challenge them by predicting experimental measurements not used for their inference, that is, the spatial arrangement of methylation marks along the DNA sequence, as summarized in the connected correlation function defined as ⟨*m*_*i*_*m*_*j*_⟩ − ⟨*m*_*i*_⟩⟨*m*_*j*_⟩, where *m*_*i*_ is the methylation state at genomic position *i* and the ensemble average ⟨…⟩ is taken over cells and comparable genomic loci (Fig. 3a, Extended Data Fig. 2a and ‘Correlation and cross-correlation functions’ section). The correlation function is resolved on the single-cell level by our scNMT-seq experiment (see ‘Processing of scNMT-seq 2i release data’ section). The quantification of gene expression in sequenced cells shows that two genes encoding enzymes that methylate DNA, namely, the de novo methyl transferases *Dnmt3b* and *Dnmt3l* (ref. 29), were expressed in most cells and a third one, *Dnmt3l*, was lowly expressed in a subset of cells 24 h after release from 2i conditions (Fig. 3b,c and Extended Data Fig. 2b). As expected from the expression of these genes, global DNA methylation increased monotonically throughout the time course (Fig. 2c). In a seeming contradiction to our prediction, on the global level, DNA accessibility increased slightly over time (Extended Data Fig. 2c), as discussed below.

Following ref. 19, we found that the connected correlation function decays in two spatial regimes separated by the characteristic distance between methylated CpGs. For short genomic distances (∣*i* − *j*∣ ≪ 1/⟨*m*⟩), active feedback between DNA methylation and chromatin conformation leads to a decay of correlations that follow the shape of the interaction kernel. In this regime, connected correlation functions scale as $C(|i-j|)\sim |i-j{|}^{-{\left(1/3\right)}^{\left(1+\langle m\rangle \right)}}$, where ∣*i* − *j*∣ is the distance between any two base pairs and ⟨*m*⟩ denotes the average methylation level. On larger length scales, correlation functions decay as $C(|i-j|)\approx |i-j{|}^{-{\left(10/9\right)}^{\left(1+\langle m\rangle \right)}}$ (Fig. 3a, Extended Data Fig. 2a and Supplementary Information, sections IV.1 and IV.2), reflecting a passive contribution of DNA methylation to chromatin compaction. At larger distances, however, the finite size of DNMT3-enriched domains introduces a natural cut-off: interactions no longer propagate freely but are averaged over entire condensates. This suppresses long-range contributions and modifies the exponent describing correlations at long distances. The position of the crossover between both regimes decreases with the average level of DNA methylation (Fig. 3a,d and Supplementary Information, section IV.3).

**Extended Data Fig. 3:**
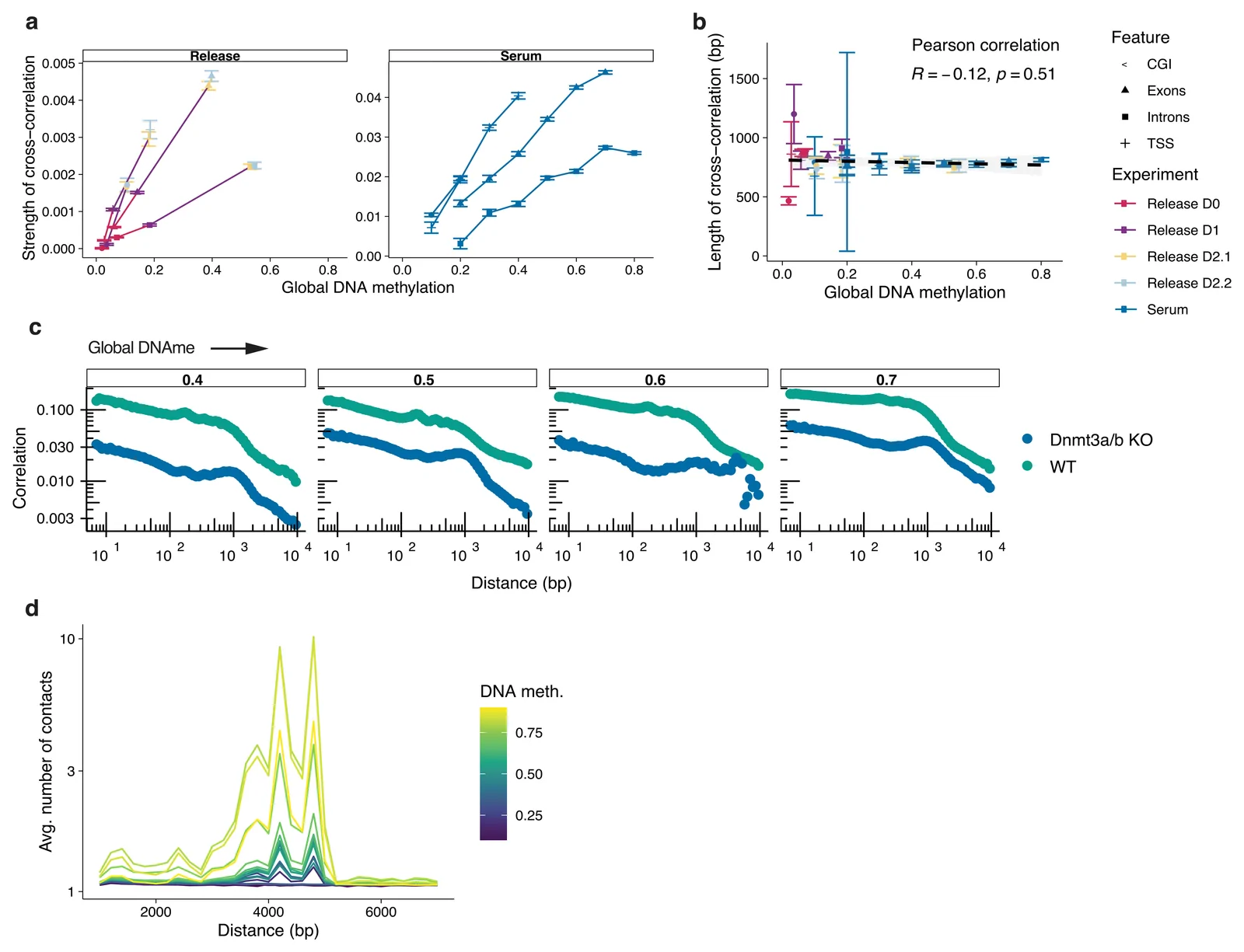
Interdependence of DNA methylation and chromatin structure. From the functional form of the cross-correlation function between DNA methylation (CG methylation) an DNA accessibility (GC methylation) obtained in Supplementary information V, cc(s) = Ae-s/lcc, we estimated (**a**) the strength, A (n = 64 cells for D0, n = 62 for D1, n = 38 for D2.1, n = 44 for D2.2), and (**b**) the length scale, lcc, of the cross-correlation between DNA methylation and accessibility using nonlinear least squares fitting. Error bars indicate standard errors. Dots/lines represent means. The p-value was obtained using a two-sided t-test on the Pearson correlation coefficient (n = 208 cells). The colour scale in (a) is the same as in (**b**). (**c**) Comparison of correlation functions of Dnmt3a/b knockout (KO) and wildtype (WT) mouse embryonic stem cells. Data was taken from and processed as in ref. 13. (**d**) Average number of contacts between DNA fragments of the DNA as a function of their one-dimensional distance, grouped by DNA methylation levels of the genomic windows.

Figure 3d shows that the empirical correlation function obtained from the scNMT-seq experiments is in excellent agreement with our theoretical results. Although the model does not have any free parameters, it quantitatively predicts the shape of correlation functions in a variety of different regions of the DNA (Fig. 3d), including gene bodies and CpG islands. The model also predicts the genomic arrangement of DNA methylation in steady-state serum cells in a range of global methylation levels (Fig. 3d) and the local association between DNA methylation and DNA accessibility (Supplementary Information, section V), quantified in a connected cross-correlation (Fig. 3e and Extended Data Fig. 3a,b). Our model is able to capture spatial-correlation functions for CpGs sites which are up to 10^4^ bp apart. However, corrections induced by DNA loops and contacts at distal loci are expected to change the functional form of the correlation functions beyond this length scale^30^. This corrections are not captured by our coarse-grained model and will require an explicitly account of the DNA in the three-dimensional space without our renormalization approach. Finally, since our model predicts that during de novo DNA methylation positive feedback mediated by chromatin compaction leads to correlated DNA methylation patterns (Fig. 1e and Fig. 3e), we expect that these correlations are reduced in the absence of DNMT3A/B enzymes. To test this prediction, we analysed scBS-seq data from *Dnmt3a*/*b* knockout mESCs cultured in serum conditions^13^. Indeed, *Dnmt3a/b* knockout mESCs consistently showed strongly reduced correlations in residual DNA methylation patterns compared with wild-type cells with comparable global average DNA methylation levels (Extended Data Fig. 3c).

### Super-resolution microscopy

**Fig. 4:**
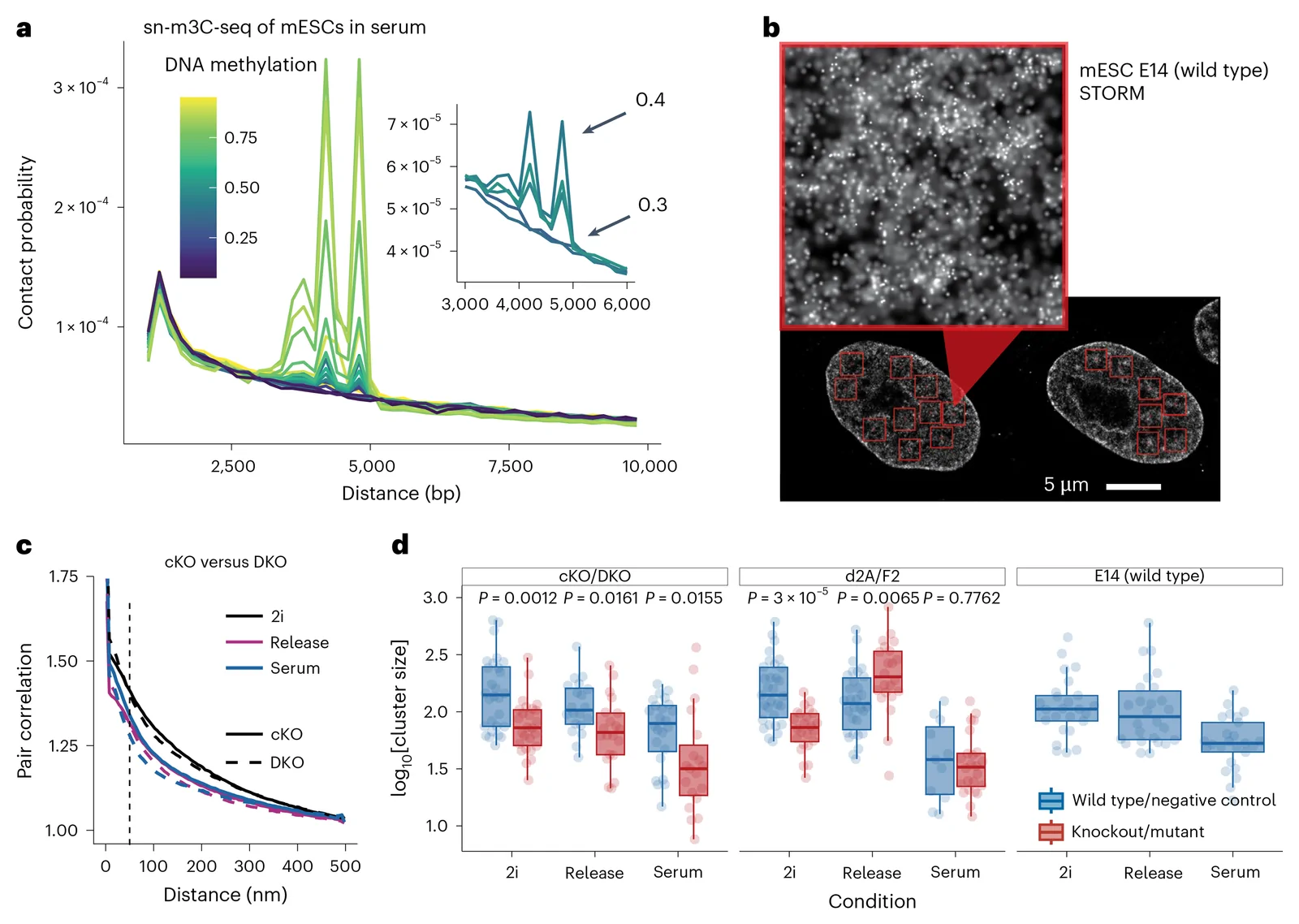
Nanoscale chromatin structures from sn-m3C-seq and STORM microscopy. **a**, Probability distribution of *cis* contacts for windows of 100-kb size, grouped by average DNA methylation level in sn-m3C-seq data in of mESCs in serum (Extended Data Fig. 3d). **b**, Super-resolution image from STORM microscopy of cells in cKO conditions. The size and intensity of each blob indicates the precision of localization. Inset: magnification of one of the areas selected for analysis (red squares). The red rectangles denote regions of interest used for downstream analysis. **c**, Pair-correlation function for localizations in *Dnmt3a*/*b* DKOs and their negative control (conditional knockouts). For visual clarity, the *y* axis was clipped at 1.75 (Extended Data Fig. 5 shows all the conditions). **d**, Box plots of cluster sizes of labelled histones for wild-type/control cell lines (E14 and cKO), *Dnmt3a*/*b* DKO and cell lines from ref. 49 (d2A/F2), where *Dnmt3a* and *Dnmt3b* are permanently deleted. The bottom and top hinges correspond to the first and third quartiles, respectively. The whiskers represent the range of the data up to 1.5 times the interquartile range. Dots represent averages of individual cells. *P* values were obtained using the Wilcoxon test. *n* = 30 cells for CKO/2i, d2A/release; *n* = 25 for cKO/release, CKO/serum, E14 serum, F12 serum; *n* = 32 for d2A/2i; *n* = 29 for DKO/2i; *n* = 26 for E14/2i, E14/release, d2A/serum; *n* = 27 for F12/2i; *n* = 22 for F12/release; *n* = 21 for DKO/serum. The larger apparent estimated size of clusters in 2i compared with serum conditions is a consequence of a looser localization of nucleosomes in 2i conditions (Extended Data Fig. 5a).

Our findings so far suggest that the dynamic feedback between DNA methylation and the formation of nanoscale chromatin structure underlies the emergence of scaling and self-similarity in physical space. This feedback involves the formation of higher-order chromatin structures on larger spatial scales with increasing levels of DNA methylation (Fig. 2e,f). We reasoned that such structures should be identifiable as an excess of midrange physical contacts between pairs of genomic loci in highly methylated regions, as measured in chromatin conformation capture experiments. We, therefore, analysed single-nucleus methyl-3C-sequencing (sn-m3C-seq) data of mESCs (see ‘Processing of sn-m3C-seq data’ section)^31^ and super-resolution microscopy data (see ‘Microscopy data’ section). From the sequencing data, we found an abrupt increase in midrange contacts between 3,000 bp and 5,000 bp (translating to a diameter of roughly 30–40 nm; see ‘Numerical conversion between genomic and physical distances’ section) for regions exceeding an average DNA methylation level of 40% (Fig. 4a and Extended Data Fig. 3d), in agreement with our prediction. The sizes of these structures are again consistent with our estimate and with those estimated from other super-resolution imaging studies^25–27^.

**Extended Data Fig. 4:**
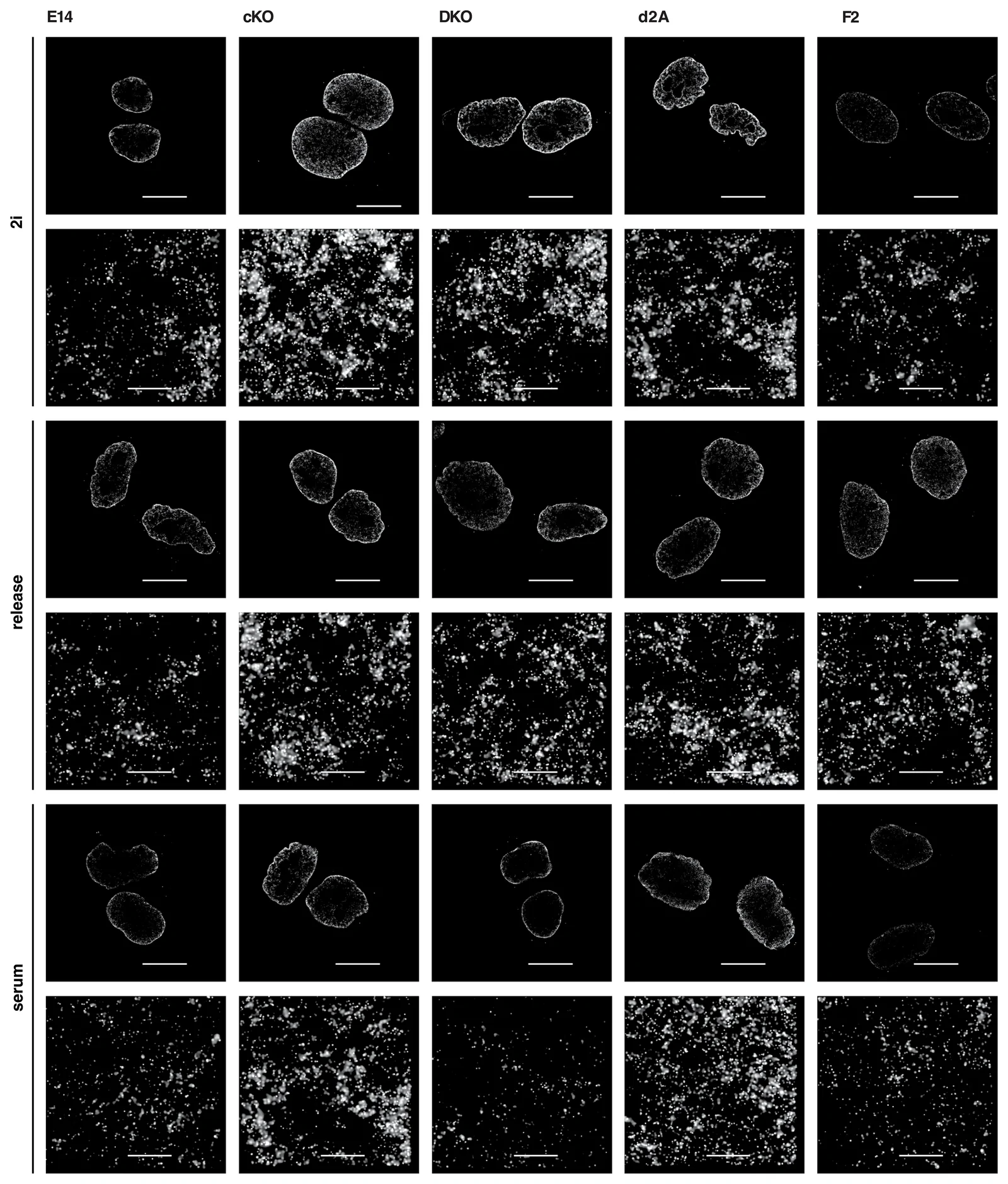
STORM images of embryonic stem cells in different culture conditions. Representative STORM microscopy images for all analysed conditions. For each condition and cell line, the top row shows full field images (scale bars: 10*μ*m and the bottom row an example of a region of interest used for downstream analysis (scale bars: 0.5*μ*m). The size and intensity of each blob indicates the precision of the localisation.

**Extended Data Fig. 5:**
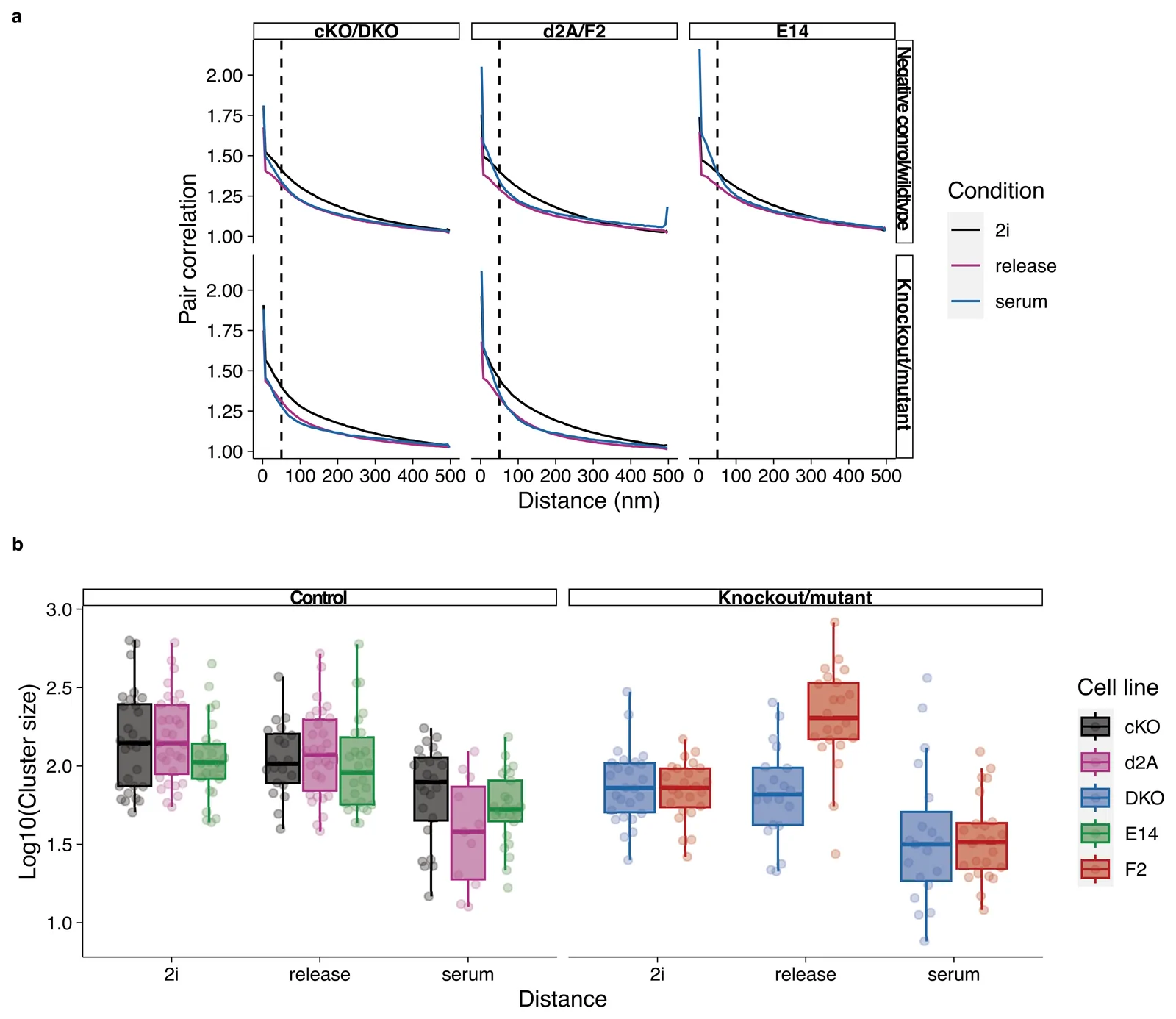
Statistical analysis of STORM localisations. (**a**) Pair correlation function of localisations in STORM microscopy images. (**b**) Variability across comparable cell lines. The lower and upper hinges correspond to the first and third quartiles, respectively. The whiskers represent the range of the data up to 1.5 times the inter quartile range. Dots represent average cluster sizes in individual cells. n = 30 for CKO/2i, d2A/release, n = 25 for cKO/release, CKO/serum, E14 serum, F12 serum, n = 32 for d2A/2i, n = 29 for DKO/2i, n = 26 for E14/2i, E14/release, d2A/serum, n = 27 for F12/2i, n = 22 for F12/release, n = 21 for DKO/serum.

To further test the dynamic feedback between DNA methylation and the formation of nanoscale chromatin structures, we used super-resolution microscopy (stochastic optical reconstruction microscopy (STORM); Fig. 4b and Extended Data Fig. 4) on mouse embryonic cells (see ‘Tissue culture’ section). Compared with the scNMT-seq and sn-m3C-seq technologies, microscopy gives a direct read-out of a subset of nucleosomes in physical space. We compared different cell lines in which *Dnmt3a* and *Dnmt3b* were knocked out (double knockout (DKO)) or permanently deleted (F2) to their respective negative controls (conditional knockout (cKO) and d2A). We further used wild-type mESCs (E14). *Dnmt3a*/*b* knockouts have been shown to have little immediate effects on transcriptomes and cell fate choices in vivo at embryonic day E8.5 (ref. 32) and in vitro for the cell line used in our experiment (Methods). We compared these cell lines in 2i conditions, in serum conditions and in cells released from 2i to serum conditions (release). We used a pan-histone antibody to label histones (see ‘Immunofluorescence’ section). Pair-correlation functions show that when *Dnmt3a*/*b* are inhibited, STORM localizations become less strongly correlated, indicating less strongly localized nucleosome clusters (Fig. 4c and Extended Data Fig. 5a). We identified clusters of labelled histones (see ‘STORM imaging and analysis’ section) and found that in wild-type or control conditions, these clusters are significantly larger than in *Dnmt3a*/*b* knockouts for all analysed cell lines (Fig. 4c,d and Extended Data Fig. 5b). This is in accordance with our theoretical predictions and with our observations (Fig. 4a).

### Identification of specific methylation correlations in pluripotency genes before their silencing

**Extended Data Fig. 6:**
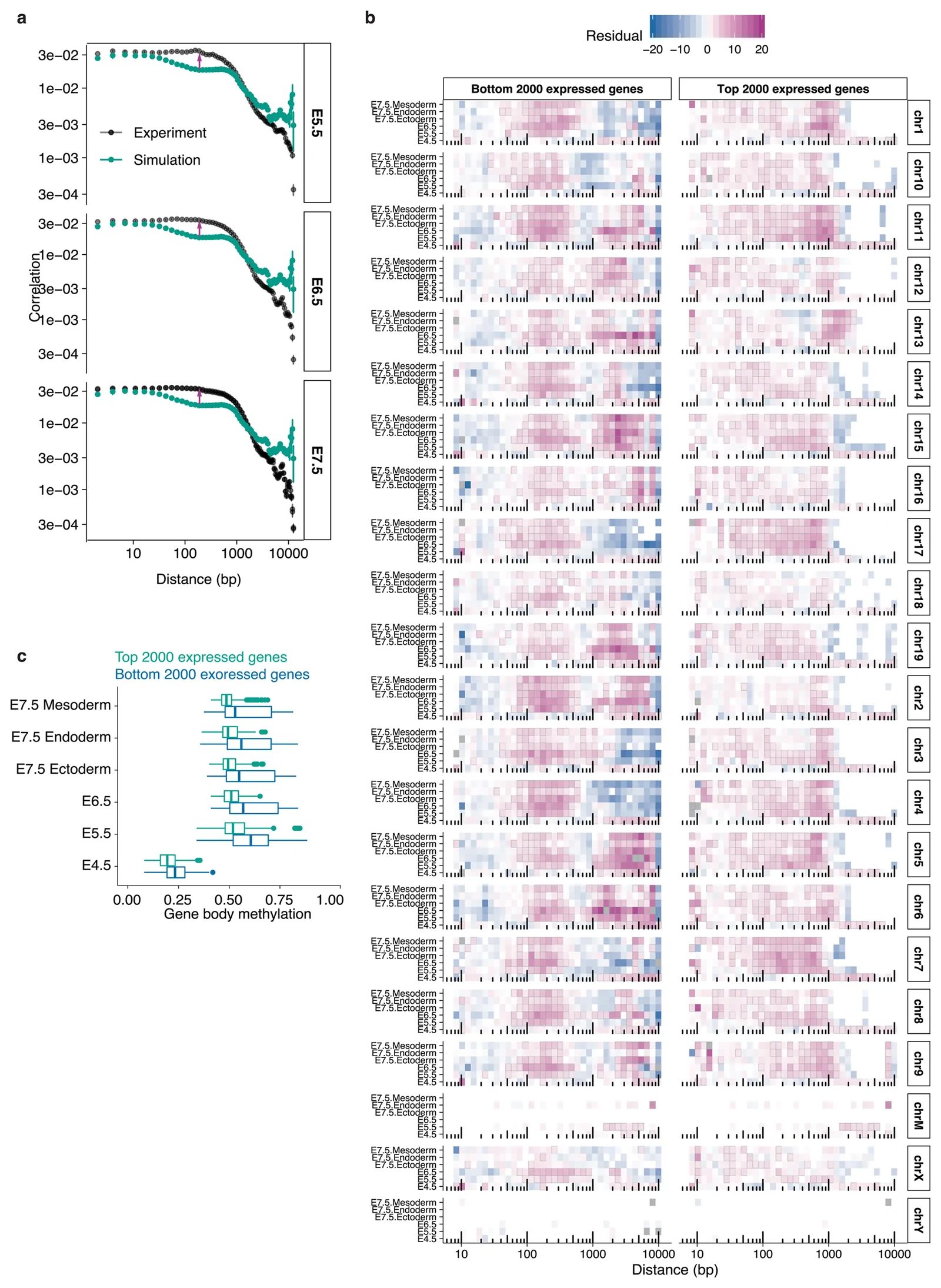
DNA methylation correlation functions in the mouse embryo. (**a**) Empirical and simulated correlation functions of DNA methylation for different stages of embryonic development. Error bars for the simulations indicate the standard deviation computed over 100 stochastic realizations of the master equation. Error bar for the experiments denotes average over all pairs of base pairs separated by the same genomic distance. The center of the dot is the average. Arrows indicate an enrichment of empirical correlations with respect to the model predictions. (**b**) Difference between simulated and empirical correlation function (residual) rescaled by the empirical standard error shown separately for each chromosome. Correlation functions were calculated for gene bodies corresponding to the bottom (left) and top (right) 2000 expressed genes. (**c**) Boxplots of average gene body methylation for both groups of genes (n = 2000 top and n = 2000 bottom expressed genes for each stage). The lower and upper hinges correspond to the first and third quartiles, respectively. The whiskers represent the range of the data up to 1.5 times the inter quartile range. The horizontal line indicates the median. Dots represent data points that deviate more than 1.5 times the inter quartile range from the median.

**Fig. 5:**
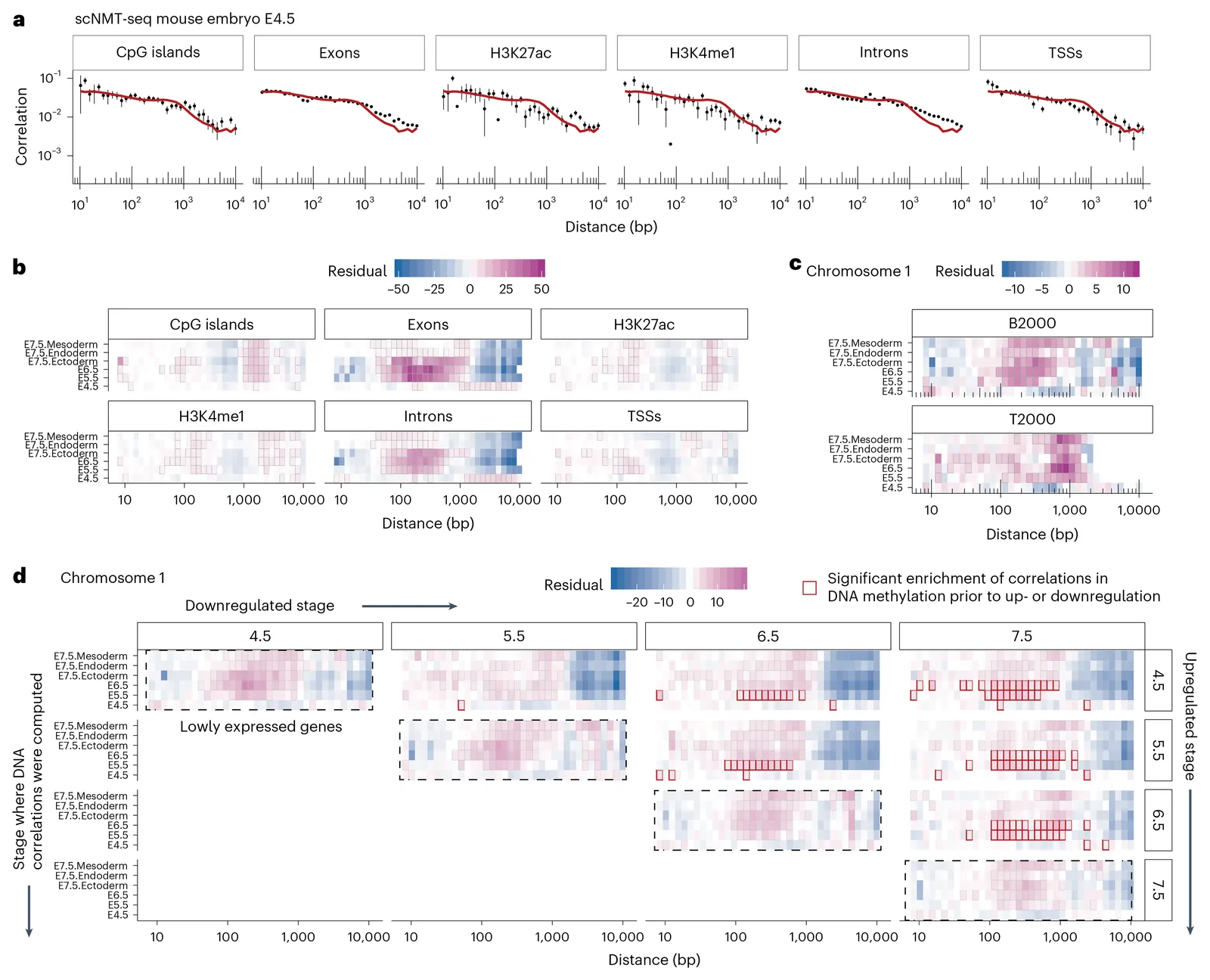
Correlated DNA methylation in the mouse embryo. **a**, Numerical prediction (red lines) and empirical correlation functions (black dots) from scNMT-seq data of mouse embryos at E4.5. Error bars denote s.e.m. Means (dots) and s.e.m. (error bars) were computed over all pairs of CpGs at a given distance apart (*n* = 59 cells). **b**–**d**, Heat maps showing differences between predicted and observed correlations in DNA methylation rescaled by the experimental standard error for a set of functional annotations (**b**), the gene bodies of the top and bottom 2,000 expressed genes (**c**) and for groups of genes that are differentially downregulated between pairs of embryonic stages (**d**). Pink regions signify an excess of correlation of the methylation state of pairs of CpGs at a given distance. Significant deviations are marked by black squares (*P* < 0.05, *t*-test) and significant deviations preceding changes in gene expression are marked by red squares.

By definition, scaling and self-similarity do not allow for encoding biological information on DNA with epigenetic marks. Therefore, we expect that when cells become primed for differentiation into more specialized cell types from E5.5 of gestation and carry lineage-dependent DNA methylation patterns^33^, scaling and self-similarity must break down. Quantifying statistical patterns of deviations from the biophysical model describing genome-wide DNA methylation dynamics (‘null model’) could identify genomic regions being specifically regulated by additional processes. To address this, we analysed scNMT-seq data from mESCs taken between E4.5 (exit from pluripotency) and E7.5 (early gastrulation)^33^. As expected, the model predicted the distribution of DNA methylation marks in pluripotent cells at E4.5 (Fig. 5a). During later stages of development (E5.5–E7.5), when cells undergo cell fate transition changes, we observed a systematic enrichment in correlations in DNA methylation on a scale between 100 and 1,000 bp (Extended Data Fig. 6a).

We found that the enrichment in correlated DNA methylation marks was specific to gene bodies, that is, introns and exons (Fig. 5b and Extended Data Fig. 6b), particularly to genes silenced between E5.5 and E7.5, but not active genes (Fig. 5c). Absolute levels of DNA methylation in active and silenced genes differed only slightly (Extended Data Fig. 6c) and, therefore, cannot fully explain these patterns.

**Extended Data Fig. 7:**
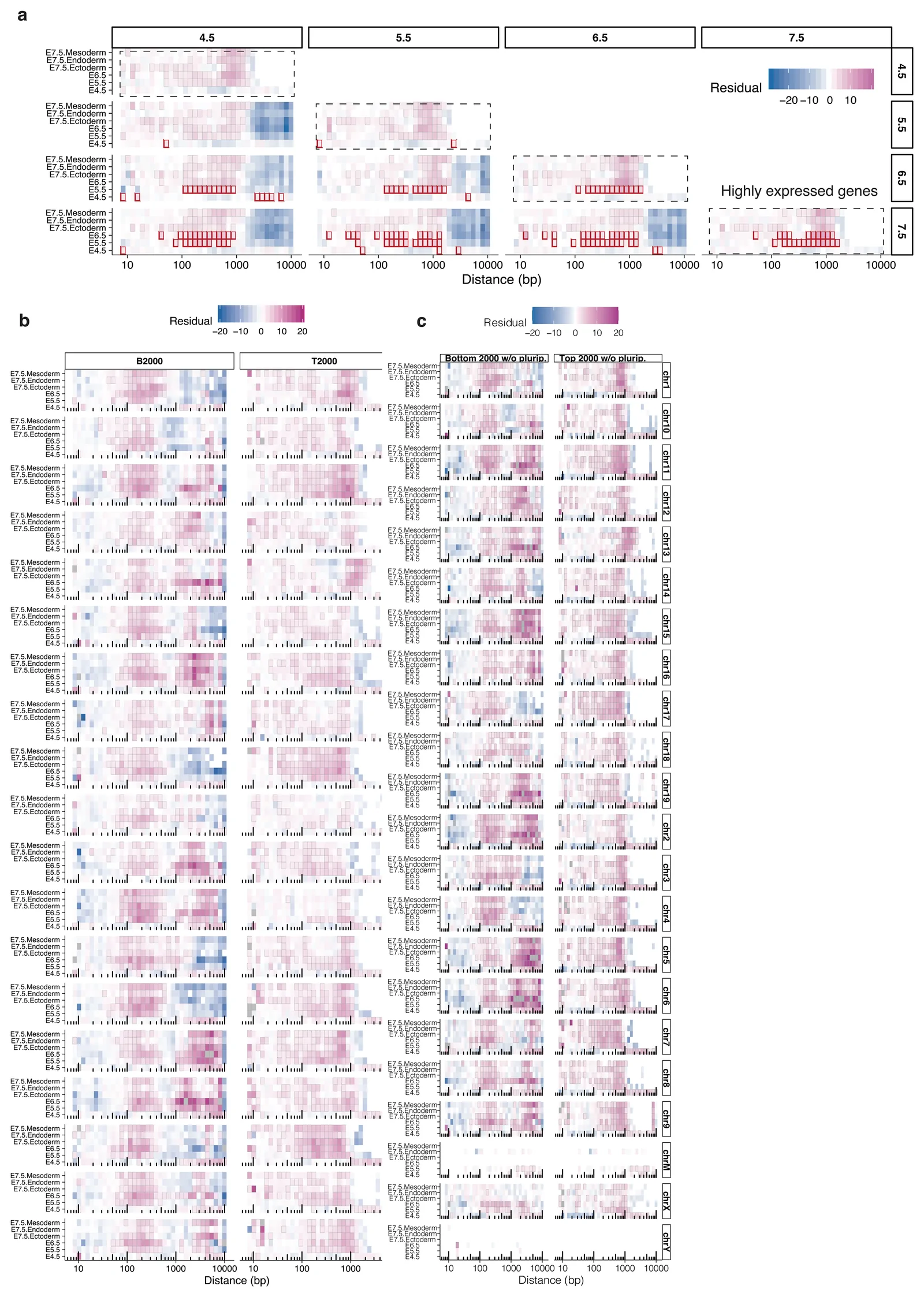
DNA methylation correlation patterns in gene bodies in the mouse embryo. (**a**) Heatmaps showing differences between predicted and observed correlations in DNAme rescaled by the experimental standard error for groups of genes that are differentially up regulated between pairs of embryonic stages, as in Fig. 5d. Significant deviations are marked by black squares (p<0.05, two-sided ttest) and significant deviations preceding changes in gene expression are marked by red squares. The same heatmaps are shown for (**b**) gene bodies of pluripotency genes, and (**c**) gene bodies of the top and bottom 2000 expressed genes excluding pluripotency genes.

We then asked whether such a pattern is a consequence of gene silencing between E5.5 and E7.5, or whether it temporally precedes the silencing of genes during differentiation. To this end, we determined differentially expressed genes between each pair of embryonic stages and for each set of genes, we calculated the enrichment or depletion in spatial correlations between DNA methylation marks in all stages and lineages. We found that for genes that are downregulated between a pair of embryonic stages, these changes in DNA methylation patterns emerge up to 2 days before changes in the transcriptome appear, suggesting that these marks could play an instructive role by priming the genes for silencing during differentiation (Fig. 5d). By contrast, we identified the DNA methylation pattern characteristic for active genes (Fig. 5c, bottom) only after genes had been activated, but not before (Extended Data Fig. 7a). We found that these patterns apply particularly to genes that regulate pluripotency (Extended Data Fig. 7b and Extended Data Table 1), but also to a set of silenced genes that are not annotated as pluripotency genes (Extended Data Fig. 7c). In the future, it may be possible to test these observations experimentally via epigenome editing.

## Discussion

The formation of the embryonic epigenome is a fundamental step in early development. It is regulated via complex biochemical networks that involve interactions between different kinds of histone modification, DNA methylation and enzyme that mediates these marks. Here we showed that the feedback between biochemical modifications of the DNA and the conformation of DNA in space leads to the scaling and self-similar behaviour of DNA methylation marks in time and sequence space. This shows that physical constraints have an important role in the emergence of the embryonic epigenetic landscape.

Understanding the origin of scaling requires tools that connect detailed molecular profiling of epigenetic states along the DNA sequence to emergent phenomena in space and time. Conventional tools from statistical genomics and machine learning lack a conceptual framework to describe and predict the dynamics of complex biological systems across vastly different spatial scales. By contrast, methods from non-equilibrium statistical physics, which are applied here in the context of single-cell genomics, provide a rigorous way to relate detailed sequencing measurements to spatiotemporal models and to make predictions about their consequences on larger scales in space and time.

Locally, the DNA compacts around methylated sites^34^ and DNMT3A/B preferentially binds to such compacted sites, leading to a positive feedback loop. This feedback loop leads to the emergence of condensates of methylated DNA (and non-compacted hypomethylated DNA) with a predicted typical size of 40 nm, which is strikingly similar to the structures (clutches) observed by high-resolution microscopy^25–27,35^. The enzymes DNMT3A and DNMT3B differ in several regulatory aspects between mouse and human, such as in a broader isoform repertoire of human DNMT3B and a stronger role of DNMT3L in mouse compared with human^36^. We expect that although such differences, like the differences between DNMT3A and DNMT3B in mouse (Extended Data Fig. 1c), probably modulate the kinetics and genomic specificity of methylation establishment, they are unexpected to alter the underlying feedback mechanism between methylation and chromatin geometry that gives rise to scaling and self-similarity.

The key predictions of our model were validated by scNMT-seq, sn-m3C-seq and super-resolution microscopy. Feedback between DNA methylation and compaction could be mediated by a biophysical mechanism such as a charge-density-driven phase transition in polyelectrolytes, but may also involve intermediary steps such as MECP2 or HP1, as well as possibly nucleosome remodelling^37–41^. Higher-order structures of several hundred nanometres in size have been shown to be independent of DNA methylation^42–45^. Phase separation mechanisms have been investigated in various contexts of cell biology, but are difficult to study in vivo. By using tools from statistical physics, our work shows a way in which phase separation phenomena can be studied based on linear DNA sequencing experiments in vivo.

Our work applies to scenarios involving large-scale de novo methylation. Beyond embryonic development, applications may also include focal de novo methylation processes if the genomic regions being methylated are much larger than the predicted size of the condensates, for example, in the establishment of germline imprints^46^, X-chromosome inactivation^47^ or the differentiation of red blood cells^48^.

## Methods

### 2i release experiments

#### 2i release cell culture

To model mouse development from the exit of naive pluripotency, we used an in vitro culture system, as previously described^9^. In this system, mESCs are grown and then released from serum-free media conditions supplemented with two chemical inhibitors (2i), namely, MEK1/2 and GSK3α/β. mESCs cultured in this study were from the E14 cell line derived previously (RRID: CVCL_C320)^50^. mESCs were cultured on tissue culture plastic precoated with 0.1% gelatine in H_2_O. Cells were cultured in a humidified incubator at 37 °C in 5% CO_2_ and 20% O_2_ and were passaged when approaching confluence (every 2 days). All cultures were subject to routine mycoplasma testing using the MycoAlert testing kit (Lonza) and always tested negative. mESCs were cultured in 2i + leukaemia inhibitory factor (LIF) in N2B27 media, composed of DMEM/F12 (Life Technologies, 12634010) and Neurobasal (Life Technologies, 21103049) in a volume/volume (v/v) ratio of 1:1, 0.1 mM of 2-mercaptoethanol, 2 mM of l-glutamine, 1:200 v/v of N2 (Life Technologies, 17502048) and 1:100 v/v of B27 supplement (Life Technologies, 17504044), supplemented with 3 μM of CHIR99021, 1 μM of PD0325901 and 20 ng ml^−1^ of LIF (all obtained from the Department of Biochemistry, University of Cambridge). For the induction of serum conditions in the 2i release experiment, cells were rinsed with PBS before the culture media were changed to serum + LIF medium consisting of DMEM (Life Technologies, 10566-016), containing 15% fetal bovine serum (Gibco, 10270106), 1× non-essential amino acids (Life Technologies, 11140050), 0.1 mM of 2-mercaptoethanol (Life Technologies, 31350-010) and 2 mM of l-glutamine (Life Technologies, 25030-024), supplemented with 20 ng ml^−1^ of LIF (Department of Biochemistry, University of Cambridge).

This tissue culture system has been shown to recapitulate key transcriptional and epigenetic changes during early development^9^. Specifically, the transition of mESC from 2i to serum conditions represents the progression from naive pre-implantation inner cell mass (E3.5–E4.5) to the primed post-implantation epiblast (E4.5–E5.5). Both epigenetic and transcriptional dynamics are largely maintained in the in vitro experiment^9^. In the 2i condition, small-molecule inhibitors (inhibitors of MEK and GSK3) maintain the cells in a ‘naive ground state’ of pluripotency, by the inhibition of ERK1/2 and GSK3β signalling^10,11,51^. Cells uniformly express naive pluripotency factors such as *Nanog, Klf4* and *Rex1*. The DNA has very low levels of methylation, similar to the epigenetic reset seen in the early embryo. Culturing cells in serum (usually with LIF) creates ‘primed’ cells for differentiation, much like the post-implantation epiblast just before gastrulation. On release to serum, the expression of naive markers (*Rex1* and *Klf4*) drops, whereas the expression of primed or ‘formative’ markers (*Fgf5, Otx2* and *Pou3f1*) increases. The cells undergo a massive wave of de novo DNA methylation (upregulation of *Dnmt3a*/*b*). This mirrors the global genome hypermethylation that occurs in the epiblast shortly after implantation.

#### 2i release whole-genome BS-seq

In the 2i release experiment, cells were lysed by removing media from culture dishes and adding 200 μl of RLT plus buffer (QIAGEN) supplemented with 0.5 mM of 2-mercaptoethanol. In the first experiment, triplicate samples were collected at 31 time points from 0 h to 56.5 h. Samples taken in the first 8.5 h after release were previously published^13^, and further processing of all samples was performed as described before. BS-seq libraries were prepared from the total nucleic acid using the bulk-cell post-bisulfite adaptor tagging method, as previously described^52^. In brief, bisulfite conversion and purification was carried out using the EZ Methylation Direct MagPrep kit (Zymo), following the manufacturers’ instructions but with half volumes. Bisulfite-converted DNA was eluted from Mag-Beads directly into 39 μl of the first strand synthesis reaction mastermix (1x Blue Buffer (Enzymatics), 0.4 mM of dNTP mix (Roche), 0.4 μM of 6NF preamp oligo (IDT)), then heated to 65 °C for 3 min and cooled on ice. Subsequently, 50 U of klenow exo– (Enzymatics) was added and the mixture was incubated on a thermocycler at 37 °C for 30 min after slowly ramping from 4 °C. Reactions were diluted to 100 μl and 20 U of exonuclease I (NEB) added and incubated at 37 °C before purification using a 0.8:1 ratio of AMPure XP beads. Purified products were resuspended in 50 μl of second strand mastermix (1x Blue Buffer (Enzymatics), 0.4 mM of dNTP mix (Roche), 0.4 μM of 6NR adaptor 2 oligo (IDT)) and then heated to 98 °C for 2 min and cooled on ice. Subsequently, 50 U of klenow exo– (Enzymatics) was added and the mixture was incubated on a thermocycler at 37 °C for 90 min after slowly ramping from 4 °C. Second strand products were purified using a 0.8:1 ratio of AMPure XP beads and resuspended in 50 μl of PCR mastermix (1x KAPA HiFi Readymix, 0.2 μM of PE1.0 primer and 0.2 μM of iTAG index primer) and amplified with nine cycles. The final libraries were purified using a 0.8:1 volumetric ratio of AMPure XP beads before pooling and sequencing. All libraries were prepared in parallel with the pre-PCR purification steps carried out using a Bravo Workstation pipetting robot (Agilent Technologies). Here 9–12 libraries were sequenced as a multiplex on one Illumina HiSeq 2000 lane using 125-bp paired-end read length.

#### 2i release scNMT-seq

Here, 0 h, 24 h and 48 h after the induction of 2i release, the cells were dissociated into single cells using Accutase before flow sorting (BD Influx) into a 96-well plate containing 2.5 μl of methylase reaction buffer (1x M.CviPI Reaction buffer (NEB), 2 U of M.CviPI (NEB), 160 μM of *S*-adenosylmethionine (NEB), 1 U μl^−1^ of RNasein (Promega), 0.1% of IGEPAL CA-630 (Sigma)). Samples were incubated for 15 min at 37 °C to methylate-accessible chromatin before the reaction was stopped with the addition of RLT plus buffer (QIAGEN) and samples frozen down and stored at –80 °C before processing. All downstream library preparation steps were performed as previously described in ref. 14.

All sequencing was carried out on HiSeq instrument. BS-seq libraries were sequenced in 96-plex pools using 125-bp paired-end reads. RNA-sequencing (RNA-seq) libraries were pooled as 96-plex or 192-plex pools and sequenced using 75-bp paired-end reads.

### Quantification and statistical analysis

#### Processing of whole-genome BS-seq data

Whole-genome BS-seq data were processed as previously described^13^. Raw sequence reads were trimmed to remove both poor-quality calls and adaptors using Trim Galore (v. 0.4.1; www.bioinformatics.babraham.ac.uk/projects/trim_galore/; Cutadapt (v. 1.8.1), parameter: –paired)^53^. Trimmed reads were first aligned to the mouse genome in the paired-end mode to be able to use overlapping parts of the reads only once as the unmapped singleton reads are written; in the second step, the remaining singleton reads were aligned in the single-end mode. Alignments were carried out with Bismark (v. 0.14.4)^54^ with the following set of parameters: (1) paired-end mode: –pbat; (2) single-end mode for Read 1: –pbat; (3) single-end mode for Read 2: defaults. Reads were then deduplicated with deduplicate_bismark, selecting a random alignment for position that were covered more than once. CpG methylation calls were extracted from the deduplicated mapping output ignoring the first 6 bp of each read (corresponding to the 6*N* random priming oligos) using the Bismark methylation extractor (v. 0.14.4) with the following parameters: (1) paired-end mode: –ignore 6 –ignore_r2 6; (2) single-end mode: –ignore 6. SeqMonk (v. 0.32) was used to compute the methylation rates and coverage in annotated genomic regions. To quality check the BS-seq data, pairwise Pearson correlation coefficients were calculated using methylation levels averaged over 10-kb tiles. Replicates within the same time point were, on average, more highly correlated than between time points (*r* = 0.885 versus 0.866). For subsequent analyses, replicates were merged.

#### Quantification of DNA methylation dynamics

For Fig. 1d, we calculated the average DNA methylation levels for a given set of genomic regions defined by their functional annotation and average CpG density using the ‘Bisulfite methylation over feature’ pipeline in SeqMonk. To be able to identify the functional form of average methylation over time, only feature sets that had more than 1,500 reads genome wide at a given time point are shown. Averages over genomic regions were weighted by the average number of reads per CpG. To collapse the time series onto a scaling form, we made a scaling ansatz of the form ⟨*m*⟩ = *a* + *bt*^5/2^ and determined *a* and *b* using a nonlinear least squares estimate, as implemented in the R function nls with default parameters. With this, the rescaled time *τ* was defined as *τ* = *tb*^2/5^. The exponent was estimated using nonlinear least squares. To verify the robustness of the exponent with respect to log transformation of both axes, we estimated the exponent for different values of an offset parameter *c*, such that the rescaled average DNA methylation reads ⟨*μ*⟩ = *c* + *τ*^*γ*^ and all values of the time course are positive. We found that under these transformations, the estimation of the exponent was robust.

#### Processing of scNMT-seq 2i release data

##### BS-seq

Alignments of the single-cell BS-seq were performed using Bismark^54^ as well as subsequent CpG methylation and GpC accessibility calling^14^. Cells with more than 10^5.5^ reads, less than 15% CHH methylation and a mapping efficiency larger than 10% were kept for downstream analysis. As previously described^52^, average DNA methylation in a given genomic interval was calculated as *m* = (*p* + 1)/(*p* + *n* + 2), where *p* and *n* signify the number of positive or negative reads in a given genomic interval, respectively.

##### RNA-seq

The single-cell RNA-seq alignments were performed using Hisat 2 (57), as described previously^14^. Here, 226 cells with mitochondrial RNA of <0.15%, >200,000 reads and >2,000 detected genes were kept for downstream analysis. Reads were log normalized using the LogNormalize function of the Seurat package (v. 3.2.0) with standard parameters. For dimensionality reduction, the top 1,000 most highly variable genes were selected and a principal component analysis with default parameters of the Seurat package was performed. Uniform manifold approximation and projection (UMAP) was performed on the 15 principal components with the highest variance and with a minimum distance of 0.2. Clustering was performed using the FindClusters function in Seurat with the default parameters. Although no distinct cell types are expected in this experiment, a group of cells (cluster D2.2) deviates in their transcriptomic profiles from the remainder of the cells at the same time point. Marker gene analysis using the FindMarkers function in Seurat did not show any lineage-related differences, but genes encoding several ribosomal proteins and indicators of oxidative stress, such as *Mt1, Ftl1, Sod1* and *Prdx6*, potentially indicating higher cellular stress levels in these cells. Since these cells passed quality control and there is no reason to believe that this stress changes DNA methylation marks on the timescale of this experiment, we did not remove cluster D2.2 from our analysis but instead represent these cells separately in Fig. 3d,e.

#### Processing of sn-m3C-seq data

As previously described^31^, we retained cells with more than 5,000 *cis* contacts at distances longer than 10,000 bp and more than 100,000 covered CpGs. We tiled the genome into windows of 100 kb and, for each tile, calculated the average DNA methylation and *cis*-contact histograms with respect to the genomic distance. We then pooled these histograms for genomic windows of similar DNA methylation levels and normalized by the total number of *cis* contacts. Although contacts are expected to be technically enriched in guanine–cytosine-rich regions, which are typically associated with low DNA methylation levels, we observe an opposite effect in Fig. 3f. This suggests a biological rather than technical origin of the increasing number of *cis* contacts with DNA methylation level.

##### Processing of scNMT-seq data of mESCs (serum)

Data were processed as previously described^14^.

##### Processing of scBS-seq data of Dnmt3a/b knockout mESCs (serum)

Data were processed as previously described^13^.

##### Processing of scNMT-seq embryo BS-seq data

Data were processed as previously described^33^. Genome-wide correlation and cross-correlation functions were computed by dividing samples with respect to the stage (E4.5, E5.5, E6.5) and lineage (E7.5 mesoderm, E7.5 endoderm, E7.5 ectoderm).

##### Processing of scNMT-seq embryo RNA-seq data

Cells that had a percentage of mitochondrial RNA of <0.15%, nCount_RNA of >1 × 10^5^ and more than 2,500 genes with at least one read were kept for downstream analysis. Normalization was performed using the function LogNormalize from the Seurat package (v. 3.2.9). The least and most highly expressed genes were determined based on their log-normalized expression value. Differentially expressed genes between pairs of stages were determined using a *t*-test. To ensure that the statistical sample size was identical for each comparison, the top 2,000 genes based on *P* value were selected for further analysis. This number was chosen to achieve a balance between the biological significance of selected genes and the sample size necessary to calculate correlation functions. Correlation functions for a given set of genes were computed by first obtaining the coordinates of the corresponding gene bodies using biomart (v. 2.44.1), then computing the correlation functions for each gene and finally averaging over all the genes in a given stage or lineage. To compare the predictions made by our method to the embryo data, we used stochastic simulations of the inferred model taking into account the genomic distribution of CpG sites in the mouse genome. Differences between theory and experiment were rescaled by the experimental standard error of the correlation function at a given genomic distance. Differences were considered significant if *P* < 0.05 using a *t*-test.

#### Numerical conversion between genomic and physical distances

To convert sizes of compact chromatin structures with radius *r* to genomic distances in base pairs *n*, and vice versa, we, in a rough approximation, equated the spherical volume in physical space, 4/3π*r*^3^ and the volume occupied by *N* nucleosomes: $4/3\text{π}r_{\text{n}}^{3}N/p_{\text{f}}$. Here, *r*_n_ ≈ 5.5 nm is the radius of a single nucleosome and *p*_f_ = 0.64 is the random packing fraction. Genomic distances were obtained by solving for *N* and using that the distance across a pair of nucleosomes corresponds to roughly 200 bp in sequence space. Genomic and spatial distances were rounded to 1,000 bp or 10 nm, respectively, to reflect that these such conversions represent rough estimates.

### Correlation and cross-correlation functions

Connected correlation functions for a given distance *d* were defined as *C*(*d*) = ⟨*m*_*i*_*m*_*i*+*d*_⟩ − ⟨*m*_*i*_⟩⟨*m*_*i*+*d*_⟩, where *m*_*i*_ and *m*_*i*+*d*_ are the methylation states of a CpG at position *i* and *i* + *j*, respectively. The average ⟨…⟩ is performed over all pairs of CpGs that are a distance *j* apart and over all the samples within spefici genomic regions. To compute the (cross-) correlation functions, we sought to group statistically similar samples. To this end, we grouped cells with similar global levels of DNA methylation and average correlations in a given annotation over these cells. For promoters and CpG islands that show bimodal average DNA methylation levels or where methylation levels are less strictly correlated to global DNA methylation in a cell, in a given cell, we averaged over all regions with similar DNA methylation levels and then averaged over all cells. For the scNMT-seq embryo data, the samples were grouped by embryonic stage and lineage since within these groups, the variance in global DNA methylation levels is low compared with serum conditions. Analogously, connected cross-correlation functions were defined as *C*_*ma*_(*d*) = ⟨*m*_*i*_*a*_*i*+*d*_⟩ − ⟨*m*_*i*_⟩⟨*a*_*i*+*d*_⟩, where *a*_(*i*+*d*)_ is the accessibility (GpC methylation) state at position *i* + *d*. The average ⟨…⟩ was performed in the same way as the correlation functions. All correlation and cross-correlation functions, except those shown in Extended Data Fig. 3a, were normalized such that the integral over the correlation function is equal to 1. To predict the cross-correlation function, we estimated the length scale of local chromatin compaction using the nls function with the port algorithm in R.

### Microscopy data

#### Tissue culture

mESCs were cultured in N2B27 2i + LIF as well as serum + LIF conditions, as described above. Cells were maintained on tissue culture plastic precoated with 0.1% gelatine in H_2_O, in a humidified incubator at 37 °C in 5% CO_2_ and 20% O_2_ and were passaged when approaching confluence (every 2 days). The N2B27 2i + LIF media consisted of DMEM/ F12 (Life Technologies, 12634010) and Neurobasal (Life Technologies, 21103049) in v/v ratio of 1:1, 0.1 mM of 2-mercaptoethanol, 2 mM of l-glutamine, 1:200 v/v of N2 (Life Technologies, 17502048) and 1:100 v/v of B27 supplement (Life Technologies, 17504044), supplemented with 3 μM of CHIR99021, 1 μM of PD0325901 and 20 ng ml^−1^ of LIF (all from Department of Biochemistry, University of Cambridge). The serum + LIF media consisted of DMEM (Life Technologies, 10566-016), containing 15% fetal bovine serum (Gibco, 10270106), 1× non-essential amino acids (Life Technologies, 11140050), 0.1 mM of 2-mercaptoethanol (Life Technologies, 31350-010) and 2 mM of l-glutamine (Life Technologies, 25030-024), supplemented with 20 ng ml^−1^ of LIF (Department of Biochemistry, University of Cambridge).

One day before the induction for the 2i release experiment, cells cultured in N2B27 2i + LIF were plated on gelatinized plates in N2B27 2i + LIF conditions to prepare for the ‘release’ condition. In parallel, cells were cultured on gelatinized plates in N2B27 2i + LIF and serum + LIF to establish 2i and serum conditions along with release. The following day, the media were changed to serum + LIF, following a PBS wash for the release condition. For 2i and serum conditions, the media were also changed but remained the same for each condition. Then, 48 h post-2i release cells were trypsinised (TrypLE, Gibco Thermo Fisher Scientific, 12604013) and plated in ibidi glass slides (μ-Slide 4 Well Ph+, 80446) coated by vitronectin (Vitronectin XF number 07180) and were allowed to attach for 8 h. Then, 200,000 cells were plated per well of the four-well slides, to allow for optimal density for imaging. Then, 56 h post-2i release, cells were washed with PBS and fixed with 2% paraformaldehyde for 30 min at room temperature. Slides were then washed further with PBS and stored at 4 °C with PBS with 0.05% Tween-20 and 0.05% sodium azide added.

To test the effect of *Dnmt3a*/*b* knockouts on the transcriptome, we analysed single-cell RNA-seq data from *Dnmt3a*/*b* DKO mESCs^55^. The DKO cell lines used in this experiment are the same as we used for our microscopy experiments. We computed differentially expressed genes between wild type and DKO using the FindMarkers function in Seurat and performed gene set enrichment analysis using g:Profiler. We did not find any enrichment in gene categories related to cell fate or epigenetic remodelling. This suggests that in vitro, *Dnmt3a*/*b* DKOs do not lead to large-scale epigenetic or cell fate changes beyond the intended effect of the knockouts.

#### Immunofluorescence

Cells were permeabilized using 0.5% Triton X-100 in PBS for 1 h at room temperature. Cells were then washed using PBT (PBS with 0.05% Tween-20) and blocked with 1% bovine serum albumin in PBT for 1 h at room temperature. Cells were incubated overnight at 4 °C with an anti-histone antibody (MAB3422, Sigma-Aldrich) at a 1:100 concentration. The anti-histone antibody, clone H11-4, MAB3422 is a specific mouse monoclonal antibody (clone H11-4) that targets pan-histone proteins, recognizing all core histones (H1, H2A, H2B, H3, H4) with similar sensitivity. We used this antibody to label the chromatin across the nucleus without biasing towards specific chromatin states. The following day, cells were washed in 1% bovine serum albumin in PBT for 1 h at room temperature. Cells were incubated for 1 h at room temperature in the dark with the secondary antibody (donkey anti-mouse 647, Thermo Fisher/Invitrogen A31571) at a 1:1,000 concentration, followed by a PBT wash. Cells were then stained with 5 μg ml^−1^ of DAPI for 15 min and washed with PBT. Slides were stored at 4 °C with phosphate buffer.

### STORM imaging and analysis

STORM imaging was performed using a Nikon N-STORM imaging system, comprising Nikon Ti-2 microscope, Nikon ×100 1.49 Apo TIRF objective, Andor iXon 897 EM-CCD camera and Nikon LU-NV-J laser combiner. The system was operated using Nikon NIS-Elements software (v. 5.42.06). Immediately before imaging, samples were immersed in a STORM imaging buffer comprising 10 mM of cysteamine (MEA), 50 mM of sodium sulphite and 10% glycerol in 1× PBS. Samples were illuminated using 647-nm laser light with HiLo illumination using 1% laser power, before increasing the laser power to the maximum and imaging for a total of 20,000 frames, with the camera exposure time set to ‘1 frame’ to maximize the frame rate. Focal drift was minimized by engaging the Nikon PFS system; lateral drift was corrected post-acquisition using the drift correction function in NIS-Elements software. Localization analysis was performed using Nikon NIS-Elements software in regions of interest that did not overlap with the increased signal in the nuclear periphery as well as regions of the nucleus in which localization was not detected. The initial spot localization was done using a Gaussian peak fit, with default parameters for two-dimensional STORM data analysis. For the presentation of localizations (Fig. 4 and Extended Data Fig. 4), the size and brightness of a localization depend on its accuracy in the following way: the precision is equal to $\left(1/\sqrt{N})(I/2A\right)$, where *N* is the number of photons detected, *λ* is the emission wavelength and *A* is the numerical aperture of the objective lens. The full-width at half-maximum value of the point spread function function multiplied by $1/\sqrt{N}$ is the diameter of the circle. Detailed information about the number of localizations, regions of interest and cells analysed in each condition are provided in Supplementary Table 1.

To calculate the cluster sizes, we used the DBSCAN algorithm^56^ as implemented in the R DBSCAN package with parameters *ϵ* = 0.5 and minPts = 4, applied separately on each region of interest. We then calculated the average cluster size for each cell. Although the cluster size naturally scaled with the choice of *ϵ*, the difference between wild-type and knockout conditions was unaffected by this choice. The DBSCAN algorithm identified a larger average cluster size in 2i conditions compared with serum conditions, which is unexpected. The larger clusters in 2i conditions inferred with our methods are, however, consistent across all three cell lines. We, therefore, reasoned that the larger apparent cluster size in 2i conditions must be a result of the statistical nature of the STORM method and the DBSCAN algorithm used for cluster assignment. To test this, we calculated pair-correlation functions of all detected nucleosomes (Extended Data Fig. 5a) separately for each region of interest using the pcf function of the R package spatstat. explore. These pair-correlation functions show that nucleosomes are clustered in all conditions (decay of the correlation function). The statistical structure of clusters in both conditions is, however, different: although clusters in the serum condition are strongly localized (high correlations at short distances), nucleosomes in 2i conditions are comparatively correlated over longer distances (higher correlations beyond approximately 50 nm).

Extended Data Fig. 5b shows variability across cell lines. We find general consistency across replicates. An exception to this is the different behaviour of the F2 and DKO cell lines in the release experiment. This is not unexpected, because both cell lines have biologically very different means for the inhibition of DNMT3 (knockout versus mutant).

## Extended Data

**Extended Data Table 1:** Sources of genomic annotations

Description	Reference
Primed ESC H3K27ac ChlP-seq	Creyghton et al. (2010) *Proceedings of the National Academy of Sciences* 107, 21931-21936
Primed ESC H3K27me3 ChlP-seq	Creyghton et al. (2010) *Proceedings of the National Academy of Sciences* 107, 21931-21936
Primed ESC H3K4me3 ChlP-seq	Creyghton et al. (2010) *Proceedings of the National Academy of Sciences* 107, 21931-21936
Primed ESC p300 ChlP-seq	Creyghton et al. (2010) *Proceedings of the National Academy of Sciences* 107, 21931-21936
CpG Islands	Illingworth et al. (2010) *PLoS Genetics* 6, e1001134
Primed ESC low methylated regions (LMRs)	Stadler et al. (2011) *Nature* 480,490-495
Mouse embryo scNMT -seq	Clark et al. (2018) *Nature Communications* 9, 781
Primed ESC sn-m3C-seq	Li et al. (2019) *Nat. Methods,* 1-3
List of pluripotency genes	Müller (2008) *Nature* 455(7211), 401-405; Gerrard et al. (2016) *eLife* 5:e15657
DNMT3A2 and DNMT3B1 ChlP-seq	Baubec et al. (2015) *Nature* 520,243-247
Heterochromatin and euchromatin	McLaughlin et al. (2019) *Cell Reports 2d,* 1974-1985

Some data (CL phenotype, lesion duration and patient-reported outcomes) were collected only from the 19 participants enrolled in the cohort study. One person from the cohort study was missing on day 90 follow-up.

## Supplementary Material

**Supplementary information** The online version contains supplementary material available at https://doi.org/10.1038/s41567-026-03263-x.

Supplemental Information

## Figures and Tables

**Table 1:** Typical enzyme–substrate kinetics along with their respective average enzyme occupancy as a function of time and the spatial two-point connected correlation functions

Description	Interaction kernel, *K(i, j)*	First moment, ⟨*m*⟩	Correlation function, ⟨*m_i_m_j_*⟩ − ⟨*m_i_*⟩⟨*m_j_*⟩
Non-local interactions (involving topological changes in the DNA)	∝|*i* − *j*|^−λ^, λ < 1	$\propto t^{\frac{1}{1-\lambda }}$	$\begin{array}{l} |i-j{|}^{-\lambda^{1+(m)}},|i-j|\ll 1/\langle m\rangle \\ |i-j{|}^{-{\left[\frac{2}{3}(2-\lambda )\right]}^{1+(m)}},|i-j|\gg 1/\langle m\rangle \end{array}$
Oligomerization with rate *a*	*aδ* *_i,i±1_*	*at* ^2^	e^−*a*|*i*−*j*|^
Independent binding	*aδ_ij_*	*at* ^2^	*δ*(|*i* − *j*|)
Processivity with rate *a*	No interaction kernel	*at* ^3/2^	e^−*a*|*i*−*j*|^

## Data Availability

All sequencing datasets reported in this paper are available on Gene Expression Omnibus (GEO) under accession GSE166226. STORM localization data are available on Zenodo (https://doi.org/10.5281/zenodo.18965309)^57^. Raw images are available upon request.

## Code availability

Code for computing the correlation functions and STORM analysis are available via GitHub at https://github.com/srulands/inference_of_spatio-temporal_processes.

## References

[1] Macaulay IC, Voet T (2014). Single cell genomics: advances and future perspectives. PLoS Genet.

[2] Smith ZD, Hetzel S, Meissner A (2025). DNA methylation in mammalian development and disease. Nat Rev Genet.

[3] Klutstein M, Nejman D, Greenfield R, Cedar H (2016). DNA methylation in cancer and aging. Cancer Res.

[4] Smith ZD, Meissner A (2013). DNA methylation: roles in mammalian development. Nat Rev Genet.

[5] Lee HJ, Hore TA, Reik W (2014). Reprogramming the methylome: erasing memory and creating diversity. Cell Stem Cell.

[6] Jurkowska RZ, Jurkowski TP, Jeltsch A (2011). Structure and function of mammalian DNA methyltransferases. ChemBioChem.

[7] Auclair G, Guibert S, Bender A, Weber M (2014). Ontogeny of CpG island methylation and specificity of DNMT3 methyltransferases during embryonic development in the mouse. Genome Biol.

[8] Zinn-Justin J (2021). Quantum Field Theory and Critical Phenomena.

[9] Kalkan T (2017). Tracking the embryonic stem cell transition from ground state pluripotency. Development.

[10] Ficz G (2013). Fgf signaling inhibition in ESCs drives rapid genome-wide demethylation to the epigenetic ground state of pluripotency. Cell Stem Cell.

[11] Habibi E (2013). Whole-genome bisulfite sequencing of two distinct interconvertible DNA methylomes of mouse embryonic stem cells. Cell Stem Cell.

[12] Leitch HG (2013). Naive pluripotency is associated with global DNA hypomethylation. Nat Struct Mol Biol.

[13] Rulands S (2018). Genome-scale oscillations in DNA methylation during exit from pluripotency. Cell Syst.

[14] Clark SJ (2018). scNMT-seq enables joint profiling of chromatin accessibility DNA methylation and transcription in single cells. Nat Commun.

[15] Baubec T (2015). Genomic profiling of DNA methyltransferases reveals a role for DNMT3B in genic methylation. Nature.

[16] Krapivsky PL, Redner S, Ben-Naim E (2010). A Kinetic View of Statistical Physics.

[17] Sneppen K, Dodd IB (2016). Nucleosome dynamics and maintenance of epigenetic states of CpG islands. Phys Rev E.

[18] Rajavelu A, Jurkowska RZ, Fritz JR, Jeltsch A (2012). Function and disruption of DNA methyltransferase 3a cooperative DNA binding and nucleoprotein filament formation. Nucleid Acids Res.

[19] Olmeda F, Rulands S (2024). Field theory of enzyme-substrate systems with restricted long-range interactions. Phys Rev E.

[20] Jimenez-Useche I (2014). DNA methylation effects on tetra-nucleosome compaction and aggregation. Biophys J.

[21] Ramaswamy S, Toner J, Prost J (2000). Nonequilibrium fluctuations, traveling waves, and instabilities in active membranes. Phys Rev Lett.

[22] Weber CA, Zwicker D, Jülicher F, Lee CF (2019). Physics of active emulsions. Rep Progr Phys.

[23] Hilbert L (2021). Transcription organizes euchromatin via microphase separation. Nat Commun.

[24] Bauermann J, Bartolucci G, Weber CA, Jülicher F (2025). Theory of reversed ripening in active phase separating systems. Phys Rev Lett.

[25] Ricci MA, Manzo C, García-Parajo MF, Lakadamyali M, Cosma MP (2015). Chromatin fibers are formed by heterogeneous groups of nucleosomes in vivo. Cell.

[26] Ou HD (2017). ChromEMT: visualizing 3D chromatin structure and compaction in interphase and mitotic cells. Science.

[27] Xu J (2018). Super-resolution imaging of higher-order chromatin structures at different epigenomic states in single mammalian cells. Cell Rep.

[28] Vinayak V (2025). Polymer model integrates imaging and sequencing to reveal how nanoscale heterochromatin domains influence gene expression. Nat Commun.

[29] Jia D, Jurkowska RZ, Zhang X, Jeltsch A, Cheng X (2007). Structure of Dnmt3a bound to Dnmt3L suggests a model for de novo DNA methylation. Nature.

[30] Lieberman-Aiden E (2009). Comprehensive mapping of long-range interactions reveals folding principles of the human genome. Science.

[31] Li G, Liu Y, Zhang Y, Kubo N, Yu M (2019). Joint profiling of DNA methylation and chromatin architecture in single cells. Nat Methods.

[32] Clark SJ (2022). Single-cell multi-omics profiling links dynamic DNA methylation to cell fate decisions during mouse early organogenesis. Genome Biol.

[33] Argelaguet R (2019). Multi-omics profiling of mouse gastrulation at single-cell resolution. Nature.

[34] Monteagudo-Sánchez A, Noordermeer D, Greenberg MV (2024). The impact of DNA methylation on CTCF-mediated 3D genome organization. Nat Struct Mol Biol.

[35] Kant A (2024). Active transcription and epigenetic reactions synergistically regulate meso-scale genomic organization. Nat Commun.

[36] Hanna CW, Demond H, Kelsey G (2018). Epigenetic regulation in development: is the mouse a good model for the human?. Hum Reprod Update.

[37] Holm C (2004). Polyelectrolytes with Defined Molecular Architecture II.

[38] Rajavelu A (2018). Chromatin-dependent allosteric regulation of DNMT3A activity by MECP2. Nucleic Acids Res.

[39] Thorn GJ (2022). DNA sequence-dependent formation of heterochromatin nanodomains. Nat Commun.

[40] Krietenstein N, Rando OJ (2020). Mesoscale organization of the chromatin fiber. Curr Opin Genet Dev.

[41] Ortega-Alarcon D (2024). Extending Mecp2 interactome: canonical nucleosomal histones interact with Mecp2. Nucleic Acids Res.

[42] Hassan-Zadeh V, Rugg-Gunn P, Bazett-Jones DP (2017). DNA methylation is dispensable for changes in global chromatin architecture but required for chromocentre formation in early stem cell differentiation. Chromosoma.

[43] Miron E (2020). Chromatin arranges in chains of mesoscale domains with nanoscale functional topography independent of cohesin. Sci Adv.

[44] Barth R, Bystricky K, Shaban HA (2020). Coupling chromatin structure and dynamics by live super-resolution imaging. Sci Adv.

[45] Pombo A, Dillon N (2015). Three-dimensional genome architecture: players and mechanisms. Nat Rev Mol Cell Biol.

[46] Smallwood SA (2011). Dynamic CpG island methylation landscape in oocytes and preimplantation embryos. Nat Genet.

[47] Payer B, Lee JT (2008). X chromosome dosage compensation: how mammals keep the balance. Annu Rev Genet.

[48] Challen GA (2012). Dnmt3a is essential for hematopoietic stem cell differentiation. Nat Genet.

[49] Li MA (2017). A lncRNA fine tunes the dynamics of a cell state transition involving *Lin28, let-7* and *de novo* DNA methylation. eLife.

