# Supplementary material for: Scaling and self-similarity in the formation of the embryonic epigenome
Source: Nat Phys. Author manuscript; Available in PMC 2026 Oct 19. (PMC13271883; doi:10.1038/s41567-026-03263-x)
Supplement: Supplementary file 1 [file EMS214161-supplement-Supplemental_Information.pdf]

nature physics

Supplementary information https://doi.org/10.1038/s41567-026-03263-x

Scaling and self-similarity in the formation of the embryonic epigenome

In the format provided by the authors and unedited

Supplementary Information

Fabrizio Olmeda and Steffen Rulands

In this Supplementary Information, we provide detailed calculations that support the conclusions presented in the main text. The document is organized into two parts: an initial summary section that provides a concise overview of the theoretical results and which is accessible to readers without knowledge of the theoretical techniques used to derive the main conclusions. In the subsequent sections we provide the full mathematical details used to obtain these conclusions, including the model formulation, analytical derivations, and numerical analyses.

Overview over the key arguments presented in this supplemental theory

In this section, we summarize the main arguments and conclusions derived from the detailed analytical calculations presented later. It provides the conceptual framework for the results, while the full technical developments are deferred to Sections I–VI and Reference [1].

Inference of the interaction kernel from sequencing data

To begin, we outline the key steps for the inference of the interaction kernel from the time evolution of the first moment of the BS-seq experiment in Fig. 1d of the main text. To this end, we begin with a general mathematical ansatz for the stochastic dynamics of DNMT3 enzyme binding. This ansatz comprises generic enzyme kinetics: 1) binding and unbinding of enzymes to the DNA; 2) Bound enzymes can methylate the DNA. This ansatz also comprises *a priori* unknown interactions between binding events. Mathematically, these interaction are represented by an interaction kernel $J_{i,j}$ giving the rate of binding at site i given the binding state of position j.

To infer the interaction kernel, we then consider a one-dimensional lattice, where each site is described by whether it is occupied by a DNMT3 enzyme and whether it is methylated. We then describe the full stochastic dynamics of the time evolution of the probability $P(\mathbf{D}, \mathbf{m}, t)$ of observing a specific DNA methylation profile $\mathbf{m}$ and enzyme binding profile $\mathbf{D}$ at time t. $P(\mathbf{D}, \mathbf{m}, t)$ assigns statistical weights to all possible DNA methylation config-

urations along the genome. Mathematically, this time evolution is given by the master equation (10) . Because, given an enzyme is bound, DNA methylation is a linear process we can summarize enzyme binding and methylation of the DNA into one process without changing any of the results.

The mathematical strategy then works as follows: by solving the master equation for the probability $P(\mathbf{D}, \mathbf{m}, t)$ we can compare our predictions to the data and thereby infer the interaction kernel $J_{i,j}$. Obtaining the solution of the master equation is only possible approximately using stochastic simulations. Numerical methods do, however, not allow for solving the inverse problem of finding the kernel $J_{i,j}$. We therefore use analytical methods to compute certain properties of $P(\mathbf{D}, \mathbf{m}, t)$, namely the first moment (the average methylation over time) and the second moment (the spatial distribution of DNA methylation marks encoded in correlation functions). To this end, we make use of a formal analogy between the master equation and the Schroedinger equation in quantum mechanics. This allows us to use a second-quantisation formulation of the stochastic system and derive a path-integral representation of it time evolution. The use of second-quantisations and path-integral methods enable solving the inverse problem of finding $J_{i,j}$ and are key for obtaining the analytical results in this work.

We here use the first moment to infer the in interaction kernel and use the second moment to test the inferred model. As the master equation describes the probability of observing any given methylation and enzyme profile in

time, we can derive the average DNA methylation level at site i, $\langle m_i(t)\rangle$ as,

$$\langle m_i(t)\rangle = \sum_{\mathbf{D},\mathbf{m}} m_i \, P(\mathbf{D}, \mathbf{m}, t)\,. \tag{1}$$

To obtain this quantity, we perform two steps: first, we assume that the binding rate decreases with the distance to the nearest bound site with a power law described by an exponent λ. We also take a continuum limit, in which the discrete distances between between CpG sites are neglected in favour of a description of the density of bound enzymes in terms of a continuous field $\phi(s,t)$ (see below for a precise definition of this field). With this, the time evolution of DNMT3 occupancy $\phi(s,t)$ and methylation density $m(s,t)$ is given by a pair of coupled partial differential equations,

$$\begin{aligned} \frac{\partial \phi(s)}{\partial \tilde{t}} &= \phi(s)^{\lambda} + D\,\phi(s)^{\lambda-3}\,\partial_s^2 \phi(s)\,, \\ \frac{\partial m(s)}{\partial \tilde{t}} &= k(s)\,\phi(s)\,. \end{aligned} \tag{2}$$

The parameter $k(s)$ denotes the methylation rate, which can depend on the specific genomic region and the density of CpG sites.

The coupled partial differential equations can be solved analytically in the limit that spatial variations are weak (see Ref. [1] for details). The solution of Eq. (2) for the average DNA methylation is, $\langle m(t)\rangle \propto t^{1+1/(1-\lambda)}$. Comparing this results to the experimental observation where the exponent is $5/2$ we obtain that, $1 + 1/(1-\lambda) = 5/2 \to \lambda = 1/3$. This means that the enzyme binding rate at a given CpG site decreases with the distance d to the nearest bound enzymes according to a power law $d^{-\lambda}$. Specifically, if an enzyme

binds at a given site i, with the closest bound enzymes located at CpG sites $i-L$ and $i+R$, respectively, the corresponding binding rate is,

$$\frac{1}{|L|^{1/3}} + \frac{1}{|R|^{1/3}}\,. \tag{3}$$

At this stage, the interaction kernel is a mathematical result with no immediate biological or physical interpretation. To understand the biological meaning of the inferred interaction kernel, we consider how the total binding in a given genomic region centered around position i scales with its size l. To this end, we integrate the kernel over such a region and we find that the cumulative binding rate scales as $l^{2/3}$. The $l^{2/3}$-dependence is reminiscent of the surface-to-volume ratio of any (non-fractal) three-dimensional object, suggesting that the fraction of accessible sites for enzyme binding is proportional to the surface of a region of compacted chromatin. Hence, this scaling signifies a potential feedback mechanism between DNA methylation and local chromatin compaction: in this picture, increased methylation locally reduces accessibility, which in turn modulates further enzyme binding.

Transformation from sequence to physical space

To explicitly connect DNA methylation to chromatin conformation, in Section III we mathematically derive differential equations describing the time evolution of DNA methylation and chromatin conformation in the three-dimensional, physical space of the cell nucleus. To this end, we develop a geometrical, field-theoretical renormalization group framework.

The kernel derived above signifies that *de novo* methylation events locally

induce chromatin contraction, thereby generating a flux of DNA methylation toward the newly compacted region. This physical picture motivates a transition from the one-dimensional sequence space (CpG coordinate s) to a one-dimensional projection of the three-dimensional physical space (coordinate x). In this mathematical representation, physical space is represented by volumes, each described by the local methylation concentration. Assuming isotropy, this new space can be seen as a one-dimensional projection which is statistically equivalent to a full three-dimensional space. The local chromatin confirmation is described by a local, dynamic metric, which relates the size of a volume in physical space to the (constant) volume of a CpG site in sequence space.

In this representation, the stochastic dynamics of enzyme occupancy, DNA methylation and chromatin conformation in the form of a local and dynamic metric is again described by a master equation, Eq. (47). To quantify how local variations in methylation affect chromatin conformation and vice versa, we developed a geometrical renormalization group scheme. This method is conceptually similar to elasticity problems in membranes or filaments, where the local mechanical state depends on molecular concentrations [2]. However, unlike those systems, where the mechanical forces are known or imposed, here we do not enforce any specific mechanical constraint. Rather, we compute chromatin condensation self-consistently from the behavior of the DNMT3 interaction kernel itself.

To this end, we consider an unmethylated DNA segment. If a new binding event locally induces chromatin compaction, a segment i of initial length Δx_i

in physical space will contract according to,

$$\delta\Delta x_i = \Delta x_i - g_i(\Delta x_i)\,. \tag{4}$$

where Δx_i is a length element in volume i in physical space and g_i describes the unknown metric representing the interplay between methylation and conformational chromatin changes. g_i serves as the basis for the derivation of the full dynamics in the physical coordinate x. The mathematical challenge lies on the property that local changes in the chromatin conformation give rise to coordinate changes in all other part of the system and thereby to a large number of methylation fluxes, which is impossible to keep track of analytically. The idea behind our geometrical renormalization group approach is to rescale space and renormalize concentrations in such a way, that these fluxes vanish.

The analytical details are given in Section III. As a result of this approach, we obtain the stochastic time evolution of enzyme and methylation concentrations in physical space. Taking the continuum limit in space and performing a mean-field approximation, we show that in this new projected physical space the time evolution of enzyme concentration follows a partial differential equation of the form,

$$\partial_t\phi(x,t) = \phi(x,t)^\lambda + \phi(x,t)^{\lambda-3}\,\partial_x^2\phi(x,t) - r\,\phi(x,t)^\lambda\,\partial_x^2\phi(x,t)\,. \tag{5}$$

The additional, anti-diffusive term $-r\,\phi(x,t)^\lambda\,\partial_x^2\phi(x,t)$ counteracts the diffusive term $\phi(x,t)^{\lambda-3}\,\partial_x^2\phi(x,t)$. Intuitively, because both terms scale differently

with the enzyme density ϕ, there is a threshold value in the enzyme of methylation density above which the anti-diffusive term dominates, leading to the formation of condensates via a phase separation mechanism. By expanding this equation around the homogeneous state (linear stability analysis), we show that depending on the value of the parameter r, this equation may exhibit a instability giving rise to patterns with a finite wavelength. The systematic modulation of the enzyme or methylation density in physical space represent chromatin condensates.

From numerical simulations and parameter estimation (Methods section of the main text), we find that this instability emerges when the average methylation level exceeds approximately 50%, and that the resulting condensates have a characteristic length scale of approximately 5,000 base pairs.

Spatial correlation functions

In this section we summarize our analytical predictions of the spatial distribution of DNA methylation marks. We obtain these correlation function from the stochastic description of the model as described in Eq. (10). We present mathematical details in Section IV and V. The mathematical steps follow the calculations we presented in Ref. [1]. Briefly, we make use of a second-quantisation formulation of the stochastic systems, based on which we develop a coherend-state path integral description of the stochastic dynamics. Intuitively, in this description the probability that the system transits from one state at a given time to any other state at a later time is given by a sum over all possible paths that connect these states. These paths are

weighted by an action, similar to quantum field theory. We may therefore use powerful mathematical tools from quantum field theory, such as perturbation theory, Feynman diagrams or renromalisation group theory to obtain correlation functions.

The application of renormalization group theory is complicated by the fact that the interaction kernel naturally defines a length scale set by the inverse of the average methylation level, $1/\langle m \rangle$. Therefore, we cannot expect spatial structures to be self-similar, as assumed in renormalization group theory. We therefore calculated the correlation function in two different spatial regimes which are each dominated by distinct physical processes. At short genomic distances, the spatial structure of DNA methylation is dominated by the active feedback between methylation and chromatin compaction. In this regime, the connected correlation function decays according to a power-law with an exponent of $-1/3$,

$$\langle m(s,t)m(s',t)\rangle_c \sim |s-s'|^{-(1/3)}\,. \tag{6}$$

At larger separations, we need to consider higher-order interactions between DNMT3 binding events such. To this end, we employ a renormalization group technique [1] and find that correlations still decay algebraically but with a different exponent,

$$\langle m(s,t)m(s',t)\rangle_c \sim |s-s'|^{-(10/9)}\,. \tag{7}$$

Although these exponents are already in good agreement with experimental

observations, they are derived from a one-dimensional theory, whereas DNA dynamics occurs in three dimensions.

To incorporate these effects, we propose that the effective dimensionality of the system depends on the mean methylation level, decreasing as $\langle m \rangle$ increases. Intuitively, it can be understood in terms of the relationship between DNA methylation and chromatin conformation. An increase in methylation leads to chromatin condensation, and in the idealistic case a fully methylated chromatin would imply a DNA which is highly compacted, effectively decreasing its dimensionality.

The average DNA methylation does not change with respect to the dimensionality of the system, but the correlation functions does. In particular the correlation function are modified,

$$C(s-s') = \begin{cases} |s-s'|^{-\left(\frac{1}{3}\right)^{1+\langle m \rangle}}, & \text{for } |s-s'| < 350/\langle m \rangle \,, \\ |s-s'|^{-\left(\frac{10}{9}\right)^{1+\langle m \rangle}}, & \text{for } |s-s'| > 350/\langle m \rangle \,, \end{cases} \tag{8}$$

where the crossover between regimes occurs at the inverse of the mean methylation level, $1/\langle m \rangle$, and the numerical prefactor 350 is obtained from dimensional arguments. The analytical form of these connected correlation functions shows excellent quantitative agreement with experimental data.

Finally, we determine the shape of the cross-correlation function between DNA methylation and chromatin accessibility $\mathbf{a}$, as measured in our scNMT-seq experiments. Assuming that chromatin accessibility is regulated locally by DNA methylation, and neglecting higher-order contributions, we obtain

that the cross-correlation function obtains a stretched exponential shape,

$$\langle m_i a_j \rangle = \alpha \langle m \rangle \exp\left(-K_a |i-j|^{2/3}\right) . \tag{9}$$

which again matches the experimental observations. The exponent 2/3, which enters the exponential form, is a consequence of the power-law correlations of DNA methylation marks, which decay on small distances with an exponent of $-1/3$. The prefactor K_a in the exponent is proportional to the typical inverse length scale of condensates. The amplitude factor α gives the overall degree of compaction. Because the prefactor α depends linear on average DNA methylation levels, this cross-correlation function can be used to predict chromatin compaction from knowledge of DNA methylation profiles.

Contents

I Ansatz for enzyme–DNA kinetics **14**

II Inference of *de novo* DNA methylation kinetics in sequence space **17**

II.1 Coherent-state path integral formulation of the master equation 17

II.2 Semiclassical limit of the field theory 21

II.3 Inference of the interaction kernel 22

II.4 Irrelevance of processive DNA methylation on large spatial scales 24

III From sequence space to physical space **26**

III.1 Derivation of the field theory in physical space 26

III.1.1 A heuristic motivation 26

III.1.2 Rigorous derivation of the dynamics in physical space . 28

III.2 Formation of condensates in physical space 34

III.3 Order of magnitude estimate of condensate sizes 35

IV Derivation of the correlation function **36**

IV.1 An instructive example in physical space 36

IV.2 Renormalization group analysis of the full non-local kinetics in sequence space 40

IV.2.1 Short distance regime 41

IV.2.2 Long distance regime 46

IV.3 One Loop correction 48

V Derivation of cross-correlation functions **49**

VI Stochastic simulations **53**

Introduction

The Supplementary Information is organized as follows. In Section I, we define a general ansatz for the stochastic dynamics that governs the binding kinetics of interacting enzymes. In Section II, we construct a coherent-state path integral representation and infer the corresponding interaction kernel from the sequencing data. In Section III, we demonstrate how an effective description in physical space can be derived from the field theory formulated in sequence space. Sections IV and V apply renormalization group theory to obtain analytical expressions for spatial correlation and cross-correlation functions. In Section V, we discuss alternative forms of the interaction kernel, and in Section VI, we present details of the numerical simulations.

I Ansatz for enzyme–DNA kinetics

To infer the kinetics of *de novo* methylation from experimental sequencing data, we begin with a general mathematical ansatz for DNMT3 enzyme kinetics. To this end, we first model the DNA as a one-dimensional lattice in which each site represents a base pair. Because only a subset of sites are cytosines in a CpG context (and thus able to be methylated), we adopt an equivalent but more efficient description in which each lattice site corresponds to a CpG. The genomic distances between CpGs will then later be encoded in a position-dependent interaction parameter. At each site, DNMT3 enzymes can bind to the DNA and, once bound, methylate that site. In Section II we show that other processes, such as independent unbinding events and po-

tential demethylation, do not affect the predictions derived below and are therefore omitted from the coarse-grained description.

The emergence of a non-trivial power law in the average DNA methylation levels (Fig. 1, main text) and the power-law decay of methylation correlations indicate that *de novo* methylation is a collective process involving interactions among DNMT3 enzymes across extended genomic domains. We therefore assume that the binding rate of DNMT3 at position i depends on the presence of bound enzymes in its vicinity. Specifically, if the nearest bound sites are j_1 and j_2, we write the binding rate as $J_{i,j_1} + J_{i,j_2}$, where the two terms quantify the contributions from the left and right bound neighbours, respectively. In the absence of heterogeneity, $J_{i,j}$ depends only on the distance between sites, $J_{i,j} = J(|j-i|)$. Interactions restricted to the closest occupied sites on the left and right effectively bound the range of J have been previously studied at the mean-field level in [3]. The time evolution of the probability of a given DNMT3 binding and DNA methylation configuration, $P(\mathbf{D}, \mathbf{m}, t)$, follows a master equation,

$$\begin{aligned}\frac{\partial P(\mathbf{D},\mathbf{m},t)}{\partial t} =& \sum_{i=1}^{N} \sum_{l=i+1}^{N} \left(\prod_{j=1}^{l-1} J_{i,i+l}\, \bar{D}_{i+j} \right) D_l \big[D_i P(\bar{\mathbf{D}}_i, \mathbf{m}, t) - \bar{D}_i P(\mathbf{D}, \mathbf{m}, t) \big] \\ &+ \text{l.n.n.} \\ &+ \text{methylation and unbinding processes}\,,\end{aligned} \tag{10}$$

where $\mathbf{D}$ and $\mathbf{m}$ are binary vectors for DNMT3 occupancy and DNA methylation, respectively (e.g., $D_i = 1$ if site i is occupied, $D_i = 0$ otherwise). The vector $\bar{\mathbf{D}}_i$ is obtained from $\mathbf{D}$ by replacing D_i with $1 - D_i$, and $\bar{D}_i = 1 - D_i$.

Here N denotes the number of lattice sites. The interaction with the left nearest neighbours has the same structure as the term shown and is abbreviated by "l.n.n." In the context of *de novo* methylation, the nonlinear nature of DNMT3 binding is supported by experimental evidence [4]. Since linear and uncorrelated processes do not contribute, we can derive the marginal distribution,

$$P(\mathbf{D}, t) = \sum_{\mathbf{m}} P(\mathbf{D}|\mathbf{m}, t)\, P(\mathbf{m}, t)\,. \tag{11}$$

By construction of the ansatz, $P(\mathbf{D}|\mathbf{m}, t) = P(\mathbf{D}, t)$, so that the master equation for the marginal distribution retains the same structure as above,

$$\frac{\partial P(\mathbf{D}, t)}{\partial t} = \sum_{i=1}^{N} \sum_{l=i+1}^{N} J_{i,i+l} \left(\prod_{j=1}^{l-1} \bar{D}_{i+j} \right) D_l \big[D_i P(\bar{\mathbf{D}}_i, t) - \bar{D}_i P(\mathbf{D}, t) \big] + \text{l.n.n.}\,. \tag{12}$$

Fragmentation analogy. As a side remark, the dynamics can be equivalently formulated in terms of the creation of *non-methylated domains*. In this analogy, a binding event between two already occupied sites corresponds to a *fragmentation* that splits a domain into two shorter intervals. Restricting attention to the first moment, our ansatz can be recast as the fragmentation equation,

$$\frac{\partial c(x, t)}{\partial t} = \int_0^\infty \mathrm{d}y\, J(x, y - x)\, c(y, t) - c(x, t) \int_0^x \mathrm{d}y\, J(y, x - y)\,, \tag{13}$$

where $c(x, t)$ denotes the number of domains of length x at time t. For a kernel $J(x, y) = 1/x^\lambda + 1/y^\lambda$ and moments $M_\alpha = \int \mathrm{d}x\, c(x, t)\, x^\alpha$, one obtains

$M_{\alpha+\lambda+1} \sim t^{-(\alpha+\lambda)/(\lambda+1)}$. The average occupancy (the zeroth moment) thus scales as $D \sim t^{1/(\lambda+1)}$. In what follows, we provide a rigorous derivation of this result and of higher-order statistics (e.g., correlation functions) using field-theoretical methods that go beyond mean-field arguments.

II Inference of *de novo* DNA methylation kinetics in sequence space

Having defined a general ansatz for the kinetics of enzyme binding, we now infer the functional form of the interaction kernel directly from the sequencing data. To this end, we employ a coherent-state path integral formulation of the master equation (10). By taking the semiclassical limit, we compare the theoretical predictions for the first moment with experimental measurements, and we further analyze the effects of additional processes that may influence the dynamics of *de novo* DNA methylation, such as the processivity of DNMT3. Although the interaction kernel is inferred from the time evolution of the first moment, the same approach can be equivalently applied to static measurements of the correlation function.

II.1 Coherent-state path integral formulation of the master equation

We now construct a path-integral representation of the master equation that allows us to compute the first- and higher-order moments of the dynamics. Before proceeding, we introduce a Fock space in which the probability

distribution is expressed as a state vector,

$$|P(t)\rangle = \sum_{\mathbf{D}} P(\mathbf{D}, t)\, a_1^{\dagger D_1} \dots a_N^{\dagger D_N} |0\rangle \,. \tag{14}$$

Here, $a_i^\dagger$ and a_i denote the creation and annihilation operators, respectively, associated with enzyme binding at the site i, and D_i represents the number of enzymes bound at that site. These operators act on the local occupation number basis $|D_i\rangle$ as,

$$\begin{aligned} a_i^\dagger |D_i\rangle &= |D_i + 1\rangle \,, \\ a_i |D_i\rangle &= D_i |D_i - 1\rangle \,, \end{aligned} \tag{15}$$

and satisfy the standard commutation relation $[a_i, a_i^\dagger] = 1$.

Using this notation, the master equation can be formally rewritten in operator form as,

$$\partial_t |P(t)\rangle = -H|P(t)\rangle \,, \tag{16}$$

where the generator H is,

$$H = -\sum_{i=1}^{N} \sum_{l=1}^{N-i} \left(\prod_{j=1}^{l-1} J_{i,i+l} (1 - a_{i+j}^\dagger a_{i+j}) \right) a_{i+l}^\dagger a_{i+l} \left[a_i^\dagger \hat{\delta}_{D_{i,0}} - \hat{\delta}_{D_{i,0}} \right] + \text{l.n.n.} \,. \tag{17}$$

The operator $\hat{\delta}_{D_{i,0}}$ enforces a single-occupancy constraint: it equals 1 if no enzyme is bound at the site i and 0 otherwise. The expectation value of an arbitrary observable $A(\mathbf{D}, t)$ can be calculated as [5],

$$\langle A(\mathbf{D}, t)\rangle = \sum_{\mathbf{D}} A(\mathbf{D})\, P(\mathbf{D}, t) \,, \tag{18}$$

which can equivalently be expressed in operator form as

$$\langle A(\mathbf{D},t)\rangle = \langle 0| \prod_i e^{a_i} A(\mathbf{D})|P(t)\rangle \,. \tag{19}$$

Here, we have introduced the coherent-state basis $\langle 0|e^{a}$, which is a left eigenstate of the creation operator $a^\dagger$,

$$\langle 0|e^{a} a^\dagger = \sum_{n=1}^{\infty} \frac{\langle 0|}{n!} a^n a^\dagger = \langle 0|e^{a} \,. \tag{20}$$

Within this coherent-state representation, we can write the identity as

$$1 = \int \mathrm{d}\phi\, \mathrm{d}\hat{\phi}\, e^{-\hat{\phi}\phi}\, e^{\phi a^\dagger} |0\rangle\langle 0| e^{\hat{\phi} a} \,, \tag{21}$$

which will be used in the following to construct the field-theoretical representation of the master equation. To account for the single-occupancy constraint, we need to determine how the $\hat{\delta}$ operators act on the coherent states. Following the rules in [6], the expectation values of these operators are,

$$\begin{aligned}
\langle \phi| a^\dagger \hat{\delta}_{\hat{n},m} |\phi\rangle &= \frac{1}{m!}\, \hat{\phi}\, (\hat{\phi}\phi)^m \, e^{-\phi\hat{\phi}} \,, \\
\langle \phi| a \hat{\delta}_{\hat{n},m} |\phi\rangle &= \frac{1}{(m-1)!}\, \phi\, (\hat{\phi}\phi)^{m-1} \, e^{-\phi\hat{\phi}} \,, \\
\langle \phi| \hat{\delta}_{\hat{n},m} |\phi\rangle &= \frac{1}{m!}\, (\hat{\phi}\phi)^m \, e^{-\phi\hat{\phi}} \,.
\end{aligned} \tag{22}$$

Finally, the formal solution of the master equation can be written compactly as,

$$|P(t)\rangle = e^{-Ht} |P(0)\rangle \,, \tag{23}$$

where $|P(0)\rangle$ represents the initial probability distribution of enzyme-binding configurations at time $t = 0$. Following Ref. [1], any observable, such as the average DNMT3 occupancy or the mean DNA methylation level, can be expressed as,

$$A(\mathbf{D}) = \int \mathcal{D}[\phi]\,\mathcal{D}[\hat{\phi}]\,A(\boldsymbol{\phi}, \hat{\boldsymbol{\phi}} = 1)\,e^{-S[\hat{\phi},\phi]}\,, \tag{24}$$

with the action,

$$S[\hat{\phi}, \phi] = -\sum_i \phi_i(t_f) + \int_0^{t_f} \mathrm{d}t \sum_i \left[\hat{\phi}_i(t)\,\partial_t\phi_i(t) + H_i[\hat{\phi}, \phi]\right]\,, \tag{25}$$

and the generator H_i,

$$H_i[\hat{\phi}, \phi] = (1 - \hat{\phi}_i)e^{-\hat{\phi}_i\phi_i}\left[\sum_{l=1}^{N-i}\prod_{j=1}^{l-1} J_{i,i+l}(l)\,\hat{\phi}_{i+l}\phi_{i+l}(1 - \phi_{i+j}) + \text{l.n.n.}\right]. \tag{26}$$

To compute correlation functions, we first define the generating functional of correlations, $Z[\mathbf{h}, \phi, \bar{\phi}]$,

$$Z[\mathbf{h}, \phi, \bar{\phi}] = \int \mathcal{D}[\mathbf{h}, \phi, \bar{\phi}]\,e^{-S[\phi,\bar{\phi}] + \int_0^y \mathrm{d}s \int_0^{t_f} \mathrm{d}t\,\left[h(s,t)\phi(s,t) + \bar{h}(s,t)\bar{\phi}(s,t)\right]}. \tag{27}$$

Expectation values of products of observables, such as correlation functions, can then be obtained as functional derivatives with respect to the auxiliary

fields h and $\tilde{h}$. As an example correlation functions are derived as,

$$\langle \phi(s,t)\phi(y,t')\rangle = \left.\frac{\delta^2 Z[\mathbf{h},\phi,\bar{\phi}]}{\delta h(s,t)\,\delta h(y,t')}\right|_{\mathbf{h}=0}. \tag{28}$$

II.2 Semiclassical limit of the field theory

We now infer the functional form of the interaction kernel for enzyme binding from the semiclassical solution of the field theory. To this end, we consider a general class of non-local kernels of the form $J_{i,i+l} = 1/l^{\lambda}$. Analogous calculations can be performed for local or exponentially decaying kernels. As a first step, we rewrite the Hamiltonian (26) in the continuum limit. We first make use of Riemann integration, such that the spatial sum $\sum_i \Delta s$ is replaced by $\int \mathrm{d}s$. The generator H in Eq. (26) becomes

$$H[\hat{\phi},\phi] = J(1-\hat{\phi}(s))e^{-\phi\hat{\phi}}\left[\int_0^{N-s}\mathrm{d}y\,\frac{\hat{\phi}(s+y)\phi(s+y)}{y^{\lambda}}e^{-\int_0^y \mathrm{d}z\,\hat{\phi}(s+z)\phi(s+z)}\right.$$
$$\left. + \int_0^{s}\mathrm{d}y\,\frac{\hat{\phi}(s-y)\phi(s-y)}{y^{\lambda}}e^{-\int_0^y \mathrm{d}z\,\hat{\phi}(s-z)\phi(s-z)}\right], \tag{29}$$

where we have specified the binding kernel for a generic long-range process as $J_{i,i\pm}(l) = \frac{J}{l^{\lambda}}$ and assumed uniform binding rates throughout the genome. Expanding the exponential terms to first order, and retaining terms up to second order in $\frac{\hat{\phi}(s\pm y)\phi(s\pm y)}{y^{\lambda}}$, while extending the integration limits to infinity,

yields

$$H[\hat{\phi}, \phi] = J\,\Gamma(1-\lambda)(1-\hat{\phi})e^{-\phi\hat{\phi}}\left[2(\hat{\phi}\phi)^{\lambda} + (\phi\hat{\phi})^{\lambda-3}(2-3\lambda+\lambda^2)\frac{\partial^2(\phi\hat{\phi})}{\partial s^2}\right]. \tag{30}$$

Path integral over the action is dominated by the minimum of the action. We therefore employ a saddle-point approximation and minimize the action, $\frac{\delta S}{\delta\hat{\phi}(x)}|_{\hat{\phi}(x)=1} = 0$, enforcing probability conservation by setting $\hat{\phi}(x) = 1$ [5]. This yields a partial differential equation that governs the time evolution of the DNMT3 binding field $\phi(s,t)$,

$$\frac{\partial\phi(s)}{\partial\tilde{t}} = \phi(s)^{\lambda} + D\,\phi(s)^{\lambda-3}\,\partial_s^2\phi(s) + \eta(s,t)\,, \tag{31}$$

where $\tilde{t} = 2Jt\,\Gamma(1-\lambda)$ and $D = \frac{(2-3\lambda+\lambda^2)}{2} > 0$.

In the hard-boson path-integral representation used here, Eq. (29), the exponential term $\exp\left[-\int_0^y \mathrm{d}z\,\hat{\phi}(s+z)\phi(s+z)\right]$ can be approximated, for slowly varying fields, by $e^{-y\langle\hat{\phi}(s)\phi(s)\rangle}$, where $\langle\hat{\phi}(s)\phi(s)\rangle$ denotes the average product of the fields. This approximation introduce an effective exponential cutoff in the interaction range, such that correlations decay beyond a characteristic length scale $\sim y^{-\lambda}$. The interpretation of the exponential term as a screening term, justifies the expansion used above and the extension of the integration limit.

II.3 Inference of the interaction kernel

To infer the interaction kernel $J_{i,i+l}$, we compute first- and higher-order moments of the local methylation density $m(s,t)$, which, in the semiclassical

limit, follows from $\partial_t m(s,t) = k\,\phi(s,t)$. Once we compute these moments for a general class of interaction kernels, we can match the resulting theoretical predictions with experimental data to infer the functional form of interaction kernel between enzyme binding events. Here, we focus on the time evolution of the first moment of the global DNA methylation level, $m(t) = \frac{1}{N}\sum_{s=1}^{N} m(s,t)$. Predictions for higher order moments will be used later to validate our results. An equally feasible approach would be to infer the interaction kernel directly from higher moments, such as the correlation functions in Eq. (80), and to use predictions for the time evolution of the first moment to verify the inference. To begin, we sum Eq. (31) over all sites to obtain a differential equation for $\phi(t) = \frac{1}{N}\sum_{s=1}^{N}\phi(s,t)$, which is solved by,

$$\phi(t) = t^{1/(1-\lambda)}\,. \tag{32}$$

Taken together, the average level of DNA methylation increases according to,

$$m(t) = m(t=0) + k\,\frac{1-\lambda}{2-\lambda}\,t^{1+1/(1-\lambda)}\,. \tag{33}$$

Therefore, to match the experimentally observed exponent of 5/2, we find that $\lambda = 1/3$, and the inferred interaction kernel is,

$$J_{i,i+l} = \frac{1}{l^{1/3}}\,. \tag{34}$$

This means that the total binding rate at position i is given by $1/l_L^{1/3} + 1/l_R^{1/3}$, where l_L and l_R denote the distances to the left and right nearest bound enzymes, respectively. The total binding rate in a region of size l is then

given by the sum,

$$\sum_{s=1}^{l-1} \left[\frac{1}{s^{1/3}} + \frac{1}{(l-s)^{1/3}} \right] , \tag{35}$$

which scales with the size of the genomic regions as $l^{2/3}$.

II.4 Irrelevance of processive DNA methylation on large spatial scales

In this section, we explain the origin of scaling as shown in Fig. 1D of the main text. To this end, we show that on large spatial scales kinetic details do not influence the statistical properties of our mode. We here derive a renormalization argument for the case of enzyme processivity. Analogous arguments hold for other processes. As shown in Ref. [7], in a simpler version of our model, there exists a continuous phase transition to an absorbing state for vanishing values of the enzyme unbinding rate, u, and for $\lambda > 1$. In our case, where $\lambda = 1/3$, classical renormalisation group arguments cannot be applied. We can, however, still investigate fluctuations around a steady-state solution defined by a balance between binding and unbinding events, a mathematical assumption required to avoid a trivial configuration in which all sites are occupied. A term describing the unbinding of enzymes is of the form $H_u = u \sum_{i=1}^{N} (1 - a_i) \hat{\delta}_{D_i,1}$, which results in a term inside the action, $u\, \phi(\hat{\phi} - 1) e^{-\hat{\phi}\phi}$. From Eq. (30), dimensional analysis in momentum $[k]$ and time $[\tau]$ yields,

$$[\hat{\phi}] = [k]^0 [\tau]^0, \qquad [\phi] = [k]^d [\tau]^0, \qquad [S] = [k]^0 [\tau]^0, \qquad [u] = [k]^0 [\tau]^{-1} . \tag{36}$$

Note that we choose $\hat{\phi}$ to be dimensionless. Moreover, the term accounting for exclusion, $e^{-\phi\hat{\phi}}$, whose exponent must be dimensionless, has been implicitly written as $e^{-v\phi\hat{\phi}}$ following [8], where $v = [k]^{-1}$ is the discretization of the lattice.

In spatial dimension d, we obtain from Eq. (30) a system of scaling relations,

$$[k]^{d(\lambda-3)}[k]^2[J][\tau] = [k]^0[\tau]^0\,, \qquad [k]^{-d}[k]^{d\lambda}[J][\tau] = [k]^0[\tau]^0\,, \tag{37}$$

with solutions,

$$[J] = [\tau]^{-1}[k]^{-d(\lambda-3)+2}\,, \qquad [J] = [\tau]^{-1}[k]^{d(1-\lambda)}\,, \tag{38}$$

which are self-consistent only for $d = 1$, yielding $[J] = [k]^{1-\lambda}[\tau]^{-1}$. This scaling arises because the diffusion constant is not an independent parameter, but depends on the local binding rate. We now show that the processivity of DNMT3 enzymes becomes irrelevant under renormalisation. The term representing processivity in the action has the form $D_0\,\hat{\phi}\,\partial_s^2\phi$, with scaling dimensions $[D_0] = [k]^{-2}[\tau]^{-1}$. This term must be compared with the effective diffusion term characterized by $[J] = [k]^{1-\lambda}[\tau]^{-1}$. Processivity is therefore irrelevant for $\lambda < 3$, which is satisfied in our case.

Although this argument is strictly valid in regimes where the binding and unbinding of DNMT3 enzymes are approximately balanced, we do not expect processivity to play a dominant role in determining the scaling behavior.

III From sequence space to physical space

Having defined the kinetics of *de novo* methylation in the one-dimensional sequence space, in this section, we derive an effective theory for the collective dynamics in the three-dimensional space. As in our theory, DNA methylation events do not introduce additional spatial correlations (Section I),in this section we will use the terms DNMT3 binding profile and DNA methylation profile interchangeably.

III.1 Derivation of the field theory in physical space

Before presenting a rigorous derivation of the effective time evolution in physical space, we first give a heuristic explanation for the structure of the resulting partial differential equation.

III.1.1 A heuristic motivation

Starting from the Langevin equation (31) in the low-noise limit, we make an ansatz to study the flux of methylated CpGs in physical space, with coordinate x. To begin, we rewrite (31) in terms of a field $h(x,t)$ defined as the perturbation around a global homogeneous average $\phi_0(t)$, $\phi(x,t) = \phi_0(t) + h(x,t)$, such that, to second order in $h(x,t)$, the corresponding Langevin equation is simplified to,

$$\frac{\partial h(x,t)}{\partial t} = b\, h(x,t) + c^- \, h(x,t)^2 + D_0\, \partial_x^2 h(x,t) + D_1^- \, h(x,t)\, \partial_x^2 h(x,t)\,. \quad (39)$$

Here b, c^-, D_0, and D_1^- are constants from the Taylor expansion of Eq. (31), with $b = \lambda\phi_0^{\lambda-1}$, $c^- = \frac{\lambda(\lambda-1)}{2}\phi_0^{\lambda-2}$, $D_0 = D\,\phi_0^{\lambda-3}$, and $D_1^- = D(\lambda-3)\phi_0^{\lambda-4}$.

In physical space, changes in DNA topology due to *de novo* methylation kinetics lead to an additional flux term J that must conserve the total DNA methylation,

$$\frac{\partial h}{\partial t} = b\,h(x,t) + c^-\,h(x,t)^2 + D_0\,\partial_x^2 h(x,t) + D_1^-\,h(x,t)\,\partial_x^2 h(x,t) - \partial_x J\,. \quad (40)$$

This flux can only depend on powers of $h(x,t)$ and its spatial derivatives. Purely algebraic terms in $h(x,t)$ would correspond to active transport of methylated sites in the nucleus, which we neglect. To lowest order, the flux therefore takes the form,

$$J = D_c\,\partial_x h(x,t) + \xi(x,t)\,, \quad (41)$$

where ξ is Gaussian white noise with $\langle\xi\rangle = 0$ and correlation $\langle\xi(x,t)\,\xi(x',t')\rangle = 2\Gamma\,\delta(t-t')\,\delta(x-x')$. Taken together, we find,

$$\frac{\partial h}{\partial t} = b\,h(x,t) + c^-\,h(x,t)^2 + (D_0 - D_c)\,\partial_x^2 h(x,t) + D_1^-\,h(x,t)\,\partial_x^2 h(x,t) - \partial_x \xi(x,t)\,. \quad (42)$$

Therefore, according to this heuristic argument, the dynamics in physical space is described by the Edwards–Wilkinson (EW) equation with additional nonlinear terms, which are irrelevant under renormalisation. In the following, we will derive rigorously the flux terms and the noise correlations.

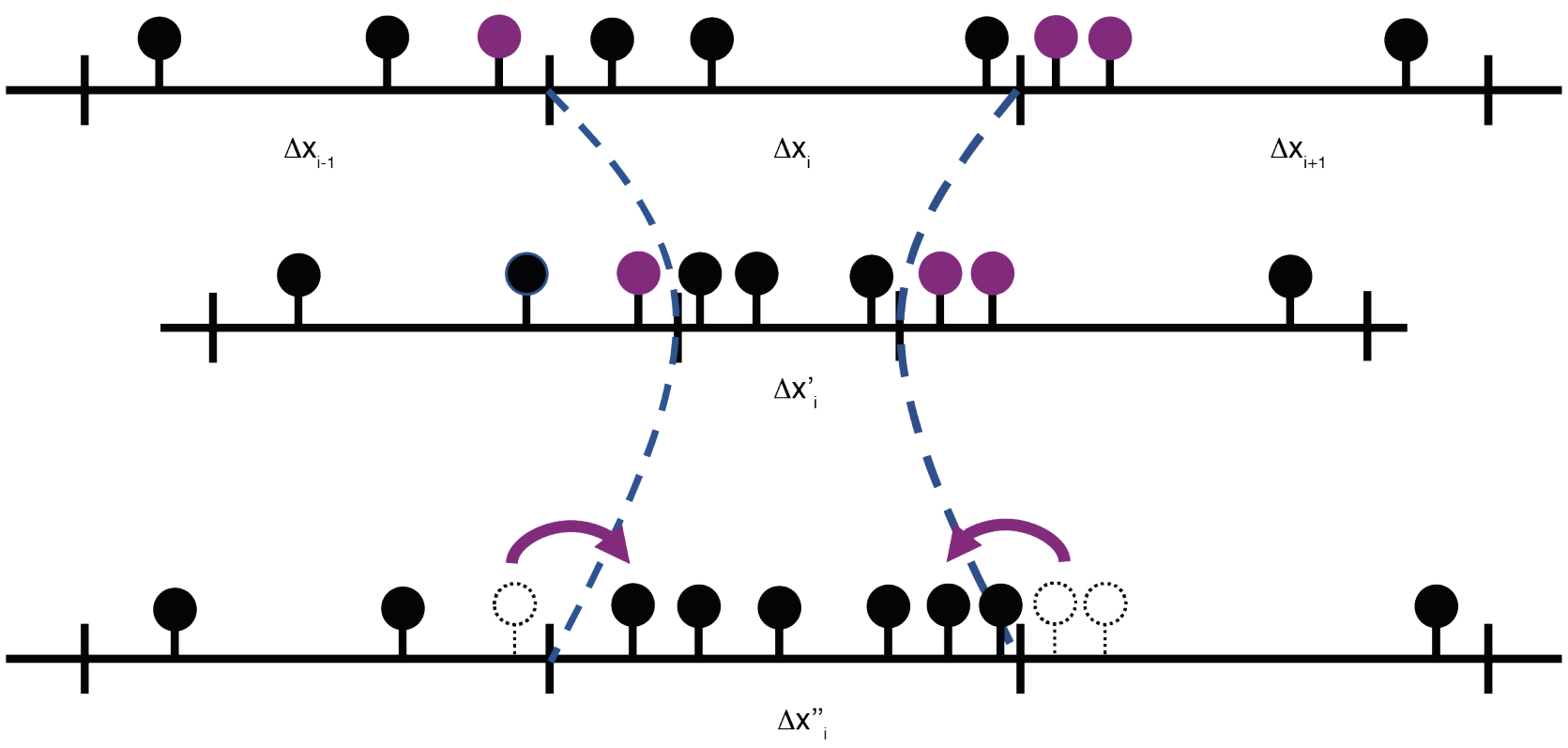

Figure S8: Geometrical renormalization group scheme for DNA compaction after methylation of CpG sites (purple circles). The method consists of two steps: first, after a methylation event the chromatin gets contracted (blue dashed line). We then renormalise space such that the total length of the system remain invariant (blue dashed line). With this procedure methylated sites of neighboring domains (purple circles) effectively lead to a flux into the contracted domain in physical space (purple arrows).

III.1.2 Rigorous derivation of the dynamics in physical space

To systematically derive the dynamics in physical space from the inferred kinetics in sequence space, we start from the partial differential equation describing the spatio-temporal dynamics in sequence space in the semiclassical limit, Eq. (31). To begin, we determine how small length elements in physical space (i.e., a projected one-dimensional space) evolve in time for a given position in sequence space. Based on this, we calculate the effective local fluxes in DNA methylation density in physical space and employ a geometric real-space renormalisation scheme to absorb directed, non-local fluxes. Specifically, we seek to define a function g_i that describes the evolution of length elements in a properly defined physical space with respect to changes

in DNA methylation density,

$$\delta\Delta x_i = \Delta x_i - g_i(\Delta x_i)\,, \tag{43}$$

with initial condition $\Delta x^0 = \Delta s$. Here, Δs and Δx denote length elements in sequence and physical space, respectively. Let $\Delta\xi_i$ be the length of a discrete element in physical space. A DNA methylation event (coarse-grained) causes a local compaction of the DNA. A length element after a binding event is contracted by,

$$\Delta\xi_i' = \Delta\xi_i^{1/3}\,. \tag{44}$$

In the absence of demethylation, continuous *de novo* methylation will therefore continuously compact the DNA locally, so that the total length of the DNA in physical space, $\Lambda = \sum_i \Delta\xi_i$, decreases over time. This induces a flux with a velocity that locally depends on the entire concentration field to the left and right of a given position. To avoid such non-local complications, we define a dynamical and geometrical real-space renormalisation scheme with renormalised length elements Δx_i such that the total length of the DNA, $L = \sum_i \Delta x_i$, remains constant in time. To achieve this, for a given *de novo* methylation event, Δx_i is first contracted according to $\Delta x_i' = \Delta x_i^{1/3}$ and then rescaled by $\Delta x_i'' = b\,\Delta x_i'$, with a rescaling factor $b > 1$ given by,

$$b = \frac{\Delta x_i + \Delta x_{i+1} + \Delta x_{i-1}}{\Delta x_i' + \Delta x_{i+1}' + \Delta x_{i-1}'}\,. \tag{45}$$

After renormalising back so that the total length is unchanged, we find that there is an effective flux of methylated sites which arises (Fig. S8) in the

vicinity of the contracted domain. We then obtain an update scheme for the concentration ρ_i of methylated sites at position i, during each DNA methylation event. Specifically, the updated concentration at a given position, ρ_i', is given by contributions from the original concentrations and symmetric fluxes from the adjacent left and right length elements,

$$\rho_i'' = \rho_i + (\rho_{i+1} + \rho_{i-1})\,\frac{\Delta x - b\Delta x^{1/3}}{2\Delta x}\,. \tag{46}$$

We now consider the joint probability $P(\boldsymbol{\rho}, t)$ of finding a given concentration profile $\boldsymbol{\rho}$ at time t. On time scales much larger than those associated with microscopic DNA methylation and compaction events, we define the rate of *de novo* methylation in a given length element i as $W(\rho_i) = \rho_i^\lambda$ with $\lambda = 1/3$. In this limit, the time evolution of $P(\boldsymbol{\rho}, t)$ is governed by a master equation for the redistribution of DNA methylation marks in physical space,

$$\begin{aligned}\partial_t P(\boldsymbol{\rho}, t) \;=\; & \sum_i \Big[W(\rho_i - r\rho_{i-1})\,P(\rho_i - r\rho_{i-1},\, \rho_{i-1} + r\rho_{i-1},\, \boldsymbol{\rho},\, t)\Big] \\ & + \sum_i \Big[W(\rho_i - r\rho_{i+1})\,P(\rho_i - r\rho_{i+1},\, \rho_{i+1} + r\rho_{i+1},\, \boldsymbol{\rho},\, t)\Big] \\ & - 2\sum_i W(\rho_i)\,P(\boldsymbol{\rho}, t)\,,\end{aligned} \tag{47}$$

where $r = (\Delta x - b\Delta x^{1/3})\big/(2\Delta x)$ is a dimensionless parameter that describes the effective flux of DNA methylation in physical space as a result of a methylation event. Here, $P(\boldsymbol{\rho}, t)$ is the probability of a given density profile at time t, and we use the notation in which, e.g., $P(\rho_{i+1} - 1, \boldsymbol{\rho}, t)$ denotes the probability of profile $\boldsymbol{\rho}$ under the condition that at position $i + 1$ the density

equals $\rho_{i+1} - 1$. The term $W(\rho_i)$ accounts for the DNA methylation rate, $W(\rho_i) = \rho_i^\lambda$. This master equation can be rewritten in terms of lowering and raising operators as,

$$\partial_t P(\boldsymbol{\rho}, t) = \sum_i \Big[L_i^{-r\rho_{i-1}} L_{i-1}^{r\rho_{i-1}} + L_i^{-r\rho_{i+1}} L_{i+1}^{r\rho_{i+1}} - 2 \Big] W(\rho_i)\, P(\boldsymbol{\rho}, t)\,, \qquad (48)$$

where the operators $L^{\pm\rho_i}$ act on functions to their right as $L^{\pm\rho_i} f(m_i) = f(m_i \pm \rho_i)$. The operator $L^{-\rho_i}$ can be written as $L_i^{-r\rho_{i-1}} = e^{-r\rho_{i-1}\partial_{\rho_i}}$ and expanded as $L_i^{-r\rho_{i-1}} = 1 - r\rho_{i-1}\partial_{\rho_i} + \mathcal{O}(r^2)$.

We now proceed with a linear-noise approximation of the master equation by splitting the observable ρ_i into deterministic and stochastic components,

$$\rho_i = N\,\phi_i + \sqrt{N}\,\eta_i\,. \qquad (49)$$

Here, N is the system size. In this form, the operators become $L_i^{-r\rho_{i-1}} = 1 - rN^{-1/2}\rho_{i-1}\,\partial_{\eta_i} + \mathcal{O}(N^{-1})$. The terms on the right-hand side of the master equation are, to lowest order in N ($\mathcal{O}(1/\sqrt{N})$),

$$\sum_i r \Big[\phi_{i-1}(-\partial_{\eta_i} + \partial_{\eta_{i-1}}) + \phi_{i+1}(-\partial_{\eta_i} + \partial_{\eta_{i+1}}) \Big] W(\phi)\, \Pi(\eta)\,. \qquad (50)$$

As fluctuations in ρ are given by fluctuations in η, we have $dP(\rho) = d\Pi(\eta)$, i.e., the probability distribution of the entire process is determined solely by its stochastic part. We now take a continuum approximation such that $\partial_{\eta_{i\pm1}} \approx \partial_{\eta(x)} \pm a_0\,\partial_x\partial_{\eta(x)}$, and likewise for $\phi_{i\pm1}$, where a_0 is the lattice spacing.

After these steps, we obtain for the right-hand side of the master equation,

$$\int dx\; r\, a_0^3\, \partial_x \phi(x,t)\, \partial_x \left[W(\phi(x))\, \frac{\delta \Pi(\eta)}{\delta \eta} \right] . \tag{51}$$

Applying the same steps to the left-hand side of the master equation we obtain to the same order in N,

$$\partial_t P(\rho, t) = \partial_t \Pi - \sqrt{N}\, a_0 \int dx\, \frac{d\phi(x,t)}{dt}\, \frac{\delta \Pi}{\delta \eta} . \tag{52}$$

Integrating by parts on the right-hand side and matching equal orders on both sides of the expanded master equation, we arrive at a partial differential equation describing the time evolution of the density field $\phi(x,t)$,

$$\partial_t \phi(x,t) = -r\, a_0^2\, W\big(\phi(x,t)\big)\, \partial_x^2 \phi(x,t) . \tag{53}$$

Equation (53) describes the flux of DNA methylation density in physical space that is the result of local, methylation-dependent DNA compaction. In general, the procedure described above modifies the functional form of long-range interactions once projected to physical space. However, at the mean-field level, such interactions yield a local term and a diffusion term with potentially different nonlinear dependencies on $\phi(x,t)$. Following the sequence-space calculation in Section I, the exponents controlling these non-linearities are not independent. Because the first moment (a genome-scale average) must coincide in sequence and physical space, the local term, and hence the diffusive term, must have the same functional form in both spaces. Therefore, in physical space, the time evolution of $\phi(x,t)$ is given by the com-

bination of the terms present in sequence space and the additional term (53) describing topology-induced flux. Substituting $W(\phi(x)) = \phi(x)^\lambda$, we obtain the partial differential equation for $\phi(x,t)$ in the renormalised physical space,

$$\partial_t \phi(x,t) = \phi(x,t)^\lambda + \phi(x,t)^{\lambda-3}\,\partial_x^2 \phi(x,t) - r\,\phi(x,t)^\lambda\,\partial_x^2 \phi(x,t)\,. \tag{54}$$

Including the next order in the system size expansion yields a noise term which, due to methylation conservation in the renormalisation procedure, is entirely conservative,

$$\begin{aligned}\partial_t \phi(x,t) = \phi(x,t)^\lambda + \phi(x,t)^{\lambda-3}\,\partial_x^2 \phi(x,t) - r\,\phi(x,t)^\lambda\,\partial_x^2 \phi(x,t) \\ +\ \eta(x,t)\ +\ \partial_x\Big[g\big(\phi(x,t)\big)\,\xi(x,t)\Big] + \dots\,.\end{aligned} \tag{55}$$

The noise terms have correlations $\langle \xi(x,t)\,\xi(x',t')\rangle = 2\Gamma_C\,\delta(t-t')\,\delta(x-x')$ and $\langle \eta(x,t)\,\eta(x',t')\rangle = 2\Gamma_{NC}\,f\big(\phi(x,t)\big)\,\delta(t-t')\,\delta(x-x')$, where the latter arises from terms proportional to $\hat{\phi}(x,t)^2$ in the field-theoretical description (29) [5]. As we focus on perturbations around dynamical homogeneous solutions, the specific dependencies in $g(\phi)$ and $f(\phi)$ are not required in the remainder of our analysis. The partial differential equation for $\phi(x,t)$ is structurally similar to Eq. (31) but contains a new term $-r\,\phi(x,t)^\lambda\,\partial_x^2\phi(x,t)$, which is anti-diffusive by counteracting the diffusion term. We expect, that as ϕ increases, this contribution can dominate diffusion and lead to the formation of highly methylated regions in physical space. Although higher-order spatial-derivative terms have not been written explicitly, they must be present. In the next section, we investigate how such terms influence the emergence of

methylation condensates. Taking into account perturbations of the previous equation, $\phi(x,t) = \phi_0 + h(x,t)$, we recover the heuristic form derived earlier in Eq. (42).

III.2 Formation of condensates in physical space

Eq. (55) highlights the emergence of new physical phenomena compared to DNA methylation dynamics in sequence space. In this section, we will systematically investigate whether spatial structures emerge in physical space. To this end, we study when a homogeneous field in physical space becomes linearly unstable upon perturbations, which would imply the emergence of a characteristic length scale resembling DNA methylation condensates. To this end we take into account the next highest order term in ϕ, $\epsilon\partial_x^4\phi$, which describes restoring forces counteracting DNA compaction at a finite length scale. Condensation occurs if a spatial perturbation, $\delta\phi$, of a homogeneous solution, ϕ_0, is unstable. Following standard procedures [9] we linearised Eq. (54) and made a general ansatz for the time evolution of the field $\phi(x,t)$ upon perturbation with wave vector k,

$$\phi(x,t) = \phi_0 + e^{\omega t} e^{ikx} \delta\phi \,. \tag{56}$$

The homogeneous state is unstable if $\omega > 0$. We obtain a dispersion relation that relates the rate of growth of the instability to the wavelength of the perturbation of the form

$$\omega(k) = \lambda\phi_0^{\lambda-1} - \left(\phi_0^{\lambda-3} - r\phi_0^{\lambda}\right) k^2 - \epsilon\phi_0^{\lambda} k^4 \,, \tag{57}$$

which is depicted in Fig. S1 for a fixed value of r and varying values of ϕ_0. Clusters of methylated DNA can form if the maximum value of this function is greater than zero for finite values of k. This latter condition is ensured by the second derivative being negative. We find that the homogeneous state becomes unstable if $\phi_0 > r^{-\frac{1}{3}}$. The strongest growing mode at the point of the instability is $k = \sqrt{r\phi_0^3 - 1}\Big/\left(\sqrt{6\epsilon}\phi_0^{3/2}\right)$, which gives an indication of the expected typical length scale of the resulting pattern. In summary, we expect an instability to arise in the form of finite size methylation condensates if the average DNA methylation concentration, ϕ_0, exceeds a threshold given by $r^{-\frac{1}{3}}$.

III.3 Order of magnitude estimate of condensate sizes

As the precise values of r and ϵ are unknown, the linear stability analysis cannot be used to identify the length scale of the predicted methylation condensates. To get an order of magnitude estimate of these condensates, we therefore resort to dimensional analysis. There are three length scales involved in the formation of methylation condensates corresponding to the parameters that determine the dispersion relation in the previous section:

1. The typical distance between methylated CpGs, l_{5mC}. For an average CpG density of roughly 1% and an average DNAme level of 50% we estimate that $l_{5mC} \approx 500\,\text{bp}$.

2. The typical length scale over which the DNAme locally affects DNA compaction, l_c. From the cross-correlation function between DNAme and accessibility, we find$l_c \approx 1000\,\text{bp}$, cf. Fig. S3B (Main text). We

expect that this length scale positively affects the size of condensates.

3. A length scale describing the restoring force that counteracts DNA compaction, l_r. We expect this to be of the same order of magnitude as the persistence length of the DNA, $l_r \approx 100\,\mathrm{bp}$, and to negatively affect the size of condensates.

Together, in order to create another length scale reflecting the typical size of condensates from these three length scales, we find $l \approx l_{5mC} l_c / l_r$ which is approximately equal to $5000 bp$.

IV Derivation of the correlation function

In this section, we give details on the calculation of the correlation function. To illustrate the idea behind the renormalisation group procedure, we will first, for didactic reasons, calculate the correlation function of a simplified model in physical space, Eq. (42). In a second step, we will then turn to the non-local dynamics in sequence space. The (connected) correlation function for the enzyme binding profile are defines as, $\langle \phi(x,t)\phi(x',t)\rangle - \langle \phi(x,t)\rangle\langle \phi(x',t)\rangle$, and, since the step involving the actual chemical modification of the DNA does not introduce further spatial correlations, it is equal to the correlation function of the DNA methylation profile.

IV.1 An instructive example in physical space

To compute scaling exponents of the correlation function, we begin by performing renormalization of Eq. (42). The idea of the renormalisation group

in momentum space is as follows: First, we transform the equation to be renormalised to Fourier space,

$$h(k,\omega) = G_0(k,\omega)\left[\xi(k,\omega) + \int_{\omega'}\int_q W(k,q)h(q,\omega)h(k-q),\omega-\omega')\right], \quad (58)$$

with $h(\mathbf{k},\omega) = \int d\mathbf{x} \int dt h(\mathbf{x},t)\, e^{i\mathbf{k}x} e^{i\omega t}$, vertex $W(k,q) = D_1^-(k-q)^2$, propagator $G_0(k) = \left(i\omega + \tilde{D}_0 k^2\right)^{-1}$ and $\tilde{D}_0 = D_0 - D_c$. The integral over momenta k must be taken in $(0,\Lambda)$ (Brillouine zone), where Λ is the inverse of the lattice spacing (1 base pair). First, we divide the interval $k \in (0,\Lambda)$ into two different intervals, $k \in (0,\Lambda e^{-l}))$ and $k \in (\Lambda e^{-l},\Lambda)$, with $l \ll 1$. The reason for this is that integrals arising from the self-consistency equation (58) are often divergent as $k \to 0$ below the critical dimension. In the following, we will discuss Wilson's momentum-shell approach to deal with this problem of infrared divergences. The first step is to integrate out short wavelengths (high momenta) on the momentum shell $k \in (\Lambda e^{-l},\Lambda)$, leaving us with an integral over $(0,\Lambda e^{-l})$. In a second step, we rescale momenta by $k \to ke^{-l}$ to make length scales comparable, which again implies a corresponding rescaling of other dimensional quantities in order to leave Eq. (42) invariant. Defining $b = e^l$, the rescaling step gives

$$t = b^z t',\ x = bx',\ \rho = b^\chi \rho'\,. \quad (59)$$

In order to perform the integration on the momentum shell we perform standard renormalisation group procedures [10] by perturbatively solving Eq. (58) for $h(x,t)$ and quantities derived from it, such as the propagator, corre-

lator and vertex. These perturbative solutions can be represented in terms of Feynman diagrams as in Fig. S9. Every vertex represents an integral over momenta, the sum of which must be equal to zero. After evaluating these diagrams we get the following flows for the parameters (other parameters become irrelevant for the exponents under renormalisation, e.g. terms in higher order spatial derivatives),

$$\begin{aligned}
\partial_l \tilde{D}_0 &= [z-2]\, D_0 \\
\partial_l D_1^- &= [z+\chi-2]\, D_1^- \\
\partial_l \Gamma_C &= [z-2\chi-d-2]\, \Gamma_C\,,
\end{aligned} \tag{60}$$

All quantities are computed in the limit of long time scales, $\omega \to 0$, and in the following, we will drop the explicit dependence on ω.

In the hydrodynamic limit, $k \to 0$ all integrals over the momentum shell are equal to 0 leading to an exact exponent identity in all spatial dimensions. In particular, the correlator, $C_0(k) = 2\Gamma_c |G_0(k)|^2$, scales as k^2 due to the conservative nature of the noise. The correlator gets a contribution to the one-loop order of k^4, as one vertex scales like $W \sim k^2$ (Fig. S9). The same correction applies to the vertex renormalization. More interestingly, the propagator does not get renormalized, meaning that mean field theory is exact at any dimension [12]. We then have $z = 2$ and substituting this in the renormalisation flow for the the correlator we obtain $\chi = -\frac{d}{2}$. Given that $\langle \rho(x,t)\rho(x',t)\rangle = |x-x'|^{2\chi} F(x/t^z)$, we find that the equal time two-point connected correlation function should scale as $c(x,x') \sim |x-x'|^{-1}$ for $d = 1$.

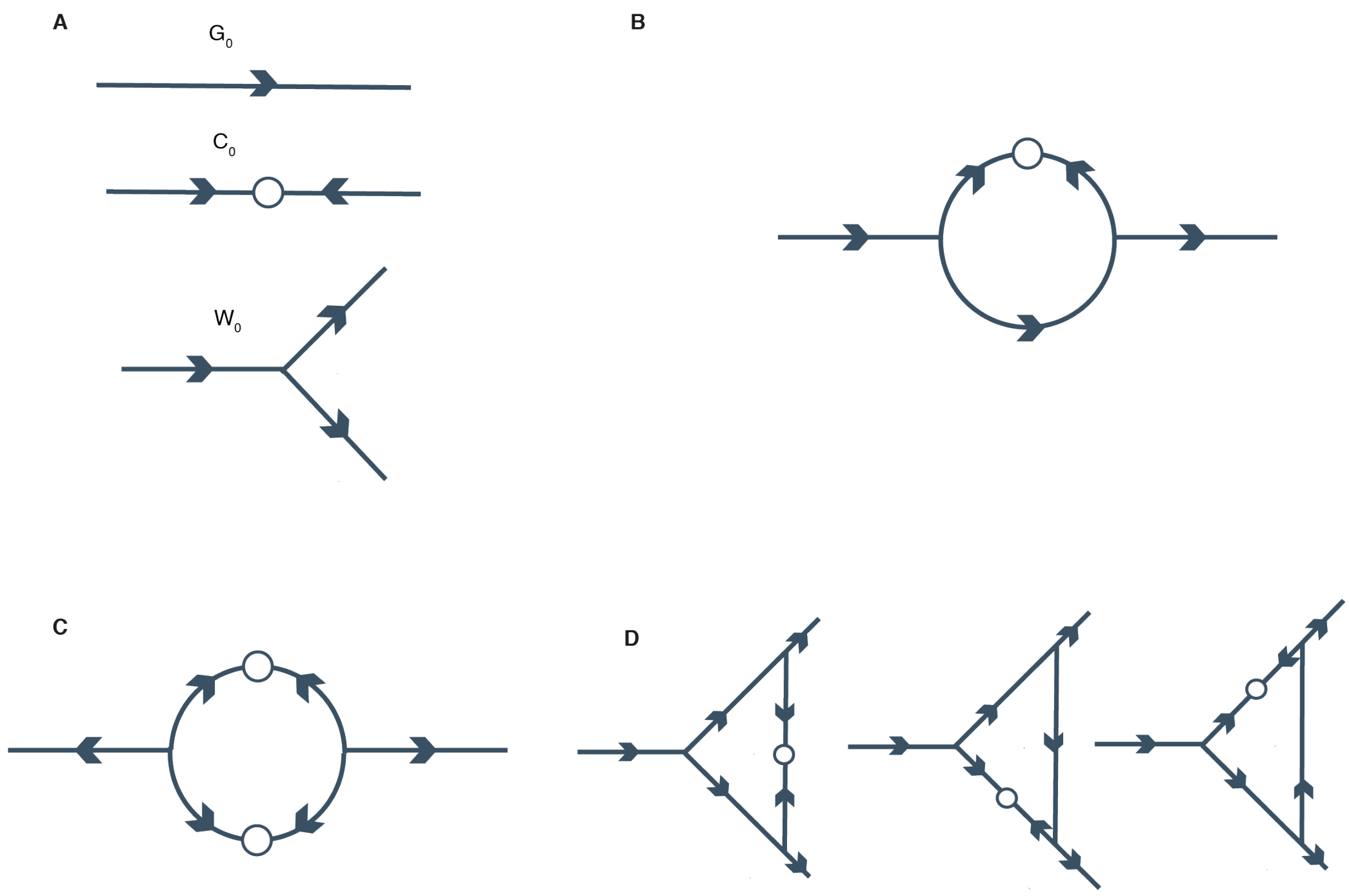

Figure S9: (A) Graphical representation for the bare propagator (G_0), correlator (C_0) and vertex (W_0) which are the basic building blocks for the renormalization group calculations.. (B, C, D): one loop corrections to the propagator, correlator and vertex respectively. The corrections are equivalent to the KPZ equation [11] and are computed as in [1, 10].

IV.2 Renormalization group analysis of the full non-local kinetics in sequence space

To compute correlation functions and scaling exponents in sequence space we might try to obtain them directly from the field theory. However, such an approach is not feasible and gives exponents that are not in agreement with our numerical simulations (calculations not shown here). The reasons for this are two-fold: First, the field theory is not massless and can be massless only without *de novo* DNA methylation. A theory where the mass term is always non zero is not scale invariant and so we will not be able to find critical exponents as observed experimentally and in the simulation. In order to overcome this problem we may try to do a change of variables in the action Eq.(29), making a Hopf-Cole transformation around a dynamical mean field solution, such that in that reference frame the theory is massless. Even though the theory will be renormalizable, the exponent does not match our stochastic simulations (calculation not shown here). One reason for the failure of such an approach is that we would need to consider perturbations of any order in the field theory and a one-loop calculation will not be sufficient. The second and main reason of the failure is that, after expanding around a base state integrals of the form (29) cannot be approximated without losing the length scale, $1/\langle\phi\rangle$, associated with the effective cutoff of interactions. The key insight to calculate the correlation function is that Eq. (29) gives rise to two spatial regimes in sequence space: for short distances interactions are long-range following a power law decay with exponent $1/3$ while for distances much larger than $1/\langle\phi\rangle$ interactions decay exponentially and are effectively

local. In the following, we will therefore derive the correlation function separately for these two regimes using renormalisation group methods, and we will confirm these results with numerical simulations.

IV.2.1 Short distance regime

The following calculations can be found in [1], here we give the connection between the theory and the physics of DNA methylation in sequence and physical space. We start by noticing that the local non-linearities are irrelevant under renormalization for conservative noise in the action (26). Importantly, conservative noise is present not only in physical space, but also in sequence space. This is due to the fact that noise can generally be derived from the field theory (29) by identifying terms that are proportional to $\hat{\phi}^2$. In the expansion of the action these terms are both non-conserative, i.e. proportional to $\hat{\phi}^2$ and conservative, i.e. proportional to $\hat{\phi}^2\partial_s^2\phi$. In order to develop a method capable to describe the short-distance regime we consider the action (29) and, after taking the semiclassical approximation, we expand it to first order in ϕ, and ∂_s,

$$\partial_t\phi(s,t) = \int_0^s dy\,\phi(y)|s-y|^{-\lambda}e^{-\int_{z=0}^{s-y} dz\,\phi(z)} + \int_0^s dy\,\partial_y\phi(y)|s-y|^{1-\lambda}e^{-\int_{z=0}^{s-y} dz\,\phi(z)}\,, \tag{61}$$

where s is the position in sequence space. For the sake of brevity, we omitted the noise terms and integrals of the same form describing interactions with the right nearest bound site. The interaction kernel Eq. (29) has the form $|s-y|^{-\lambda}e^{-\int_{z=0}^{s-y} dz\,\phi(z)}$, with $\lambda = 1/3$ in the case of *de novo* DNA methylation. By considering a perturbation $h(s,t)$ around the mean field solution, $\phi_0(t)$,

i.e. $\phi(s,t) = \phi_0(t) + h(s,t)$, Eq. (61) can be expressed to first order as

$$\begin{aligned}\partial_t \phi_0(t) + \partial_t h(s,t) = e^{-\phi_0(t)} \int_0^s dy\, \phi_0(t)|s-y|^{-\lambda} \left[1 - \int_{z=0}^{s-y} dz\, h(z)\right] + \\ e^{-\phi_0(t)} \int_0^s dy\, h(y)|s-y|^{-\lambda} \left[1 - \int_{z=0}^{s-y} dz\, h(z)\right] + \text{h.o.}\,.\end{aligned} \tag{62}$$

The first two terms on the right hand side cancel with the first one on the left hand side, which is the dynamical mean field solution. Together, we find

$$\partial_t h(s,t) = e^{-\phi_0(t)} \int_0^s dy h(y)|s-y|^{-\lambda} \left[1 - \int_{z=0}^{s-y} dz\, h(z)\right] + \text{h.o.}\,. \tag{63}$$

After a change of variables, $w = z + y$, we obtain

$$\begin{aligned}\partial_t h(s,t) =& e^{-\phi_0(t)} \int_0^s dy\, h(y)|s-y|^{-\lambda} \\ &- e^{-\phi_0(t)} \int_0^s dy \int_y^s dw\, h(y)|s-y|^{-\lambda} h(w-y) + \xi(s,t)\,.\end{aligned} \tag{64}$$

In this expression we recognize the convolution of a fractional integral of a function and the function itself. As discussed in the beginning of this section, the noise, $\xi(s,t)$, for the perturbation $h(s,t)$ has the same form that we derived in the previous section. Specifically, the equation contains both conservative and non conservative terms, $\langle \xi(s,t)\xi(s',t')\rangle = \delta(t-t')(2\Gamma_{NC} - 2\Gamma_C \partial_s^2)\delta(s-s')$. Γ_C and Γ_{NC} are the noise strengths for conservative and non conservative noise, respectively. As a side remark, while in the case of only conservative noise the non-local and non-linear term becomes relevant under renormalisation below a critical dimension $d_c = 2(2-\lambda)$. If we consider

only the non conservative noise term, the critical dimension of the system is $d_c = 2(3-\lambda)$. The non linear term is a fractional integral which general form is,

$$I^{\alpha} f = \frac{1}{\Gamma(\alpha)} \int (x-y)^{\alpha-1} f(y) \tag{65}$$

where $\Gamma(\alpha)$ is the gamma function. Upon identifying $\alpha - 1 = -\lambda$ in Fourier space the value of this integral scales as $q^{\lambda-1}$.

In order to regularise the theory we introduce an auxiliary process. The lowest order spatial derivative consistent with the symmetries of the theory is $\partial_s^2 \phi$. Taken together, taking into account interactions with the right nearest bound site we obtain in Fourier space

$$\partial_t h(q,t) = \left(e^{-\phi_0(t)} q^{\lambda-1} - q^2\right) h(q,t) - q^{\lambda-1} e^{-\phi_0(t)} h(q,t)^2 + \xi(q,t)\,. \tag{66}$$

From the dynamical mean field solution of the first moment, Eq. (32), we know that $e^{-\phi_0(t)} = e^{\left[-t^{1/(1-\lambda)}\right]}$. In the frequency domain, we then obtain for small times, $t \to 0$ or $\omega \to \infty$,

$$i\omega h(q,\omega) = (q^{\lambda-1} - q^2) h(q,\omega) - q^{\lambda-1} h(q,\omega)^2 + \xi(q,\omega)\,. \tag{67}$$

As a side remark, the inverse free propagator is $G_0^{-1} = i\omega + q^2 - q^{\lambda-1}$, which is defined based on the linear part of Eq. (67),

$$\left(i\omega + q^2 - q^{\lambda-1}\right) h(q,\omega) = \xi(q,\omega)\,. \tag{68}$$

and the correlator reads

$$C_0 = \left(2\Gamma_{NC} + 2\Gamma_C q^2\right) |G_0|^2 \,. \tag{69}$$

Continuing with our analysis, the general solution of Eq. (67) obtains the form

$$h(q,\omega) = \frac{q^{1-\lambda}}{2J}\left(-q^2 + q^{1-\lambda} + \sqrt{4q^{1-\lambda}\xi + (q^2 - q^{1-\lambda}) + i\omega} - i\omega\right) \,. \tag{70}$$

In Fourier space, the two-point correlation functions are defined as $\langle h(q,\omega)h(q',\omega')\rangle$. In the short time and large wavelength limit we keep the lowest order in q. Back Fourier transforming to real space and setting $t = t'$ we find that

$$\langle h(s,t)h(s',t)\rangle = \left[2\left(|s-s'|^2\Gamma_C + \Gamma_{NC}\lambda(1+\lambda)\right)|s-s'|^{-2-\lambda}\cos(\pi\lambda)/2\Gamma(\lambda)\right] \,, \tag{71}$$

where $\Gamma(\lambda)$ is the gamma function. We therefore obtain for the scaling of the correlation function $\langle h(s,t)h(s',t)\rangle \sim |s-s'|^{-\lambda}$ with $\lambda = 1/3$ in the case of de novo DNA methylation.

We expect that higher order corrections to this result will depend on the parameters of the model, in particular on average enzyme occupancy, ϕ_0. In order to understand this point, we may reason that the previous derivation is exact at low values of the average occupancy, ϕ_0, whilst for larger values of ϕ_0 we expect higher order corrections to become relevant. As explained above, in the context of spatial correlations, we use the terms enzyme occupancy and DNA methylation synonymously.

To understand such higher order corrections it is convenient to temporar-

ily consider the correlation function in non-renormalised physical space, and then transform back to sequence space. We begin by noting that in sequence space the correlation function decays as $|s - s'|^{-1/3}$ for vanishing values of average form, local DNA methylation. In non-renormalised physical space for vanishing average DNA methylation the correlation function must scale in the same way as in sequence space, i.e. $\sim |\xi - \xi'|^{-1/3}$. We now consider the effect of n DNA methylation events. According to Eq. (44), this leads to a contraction $\Delta\xi' = \Delta\xi^{-(1/3)^n}$. Going back to sequence space, where the length elements scale as $\Delta s \sim \Delta\xi^3$, we obtain that the correlation function decays as $|s - s'|^{-(1/3)^{n+1}}$. n is a monotonically increasing function of the local average DNA methylation level and vanishes for $\phi_0 \to 0$. Expanding to first order, we obtain approximately $n \approx \alpha\phi_0 + \ldots$ where α is a parameter that we determined to be approximately equal to 1 numerically. Correlation functions then scale as $\langle h(s,t)h(s',t)\rangle \sim |s - s'|^{-(1/3)^{1+\phi_0}}$.

From the bare propagator and correlator in Eq (69) we find that in the short wavelength regime diffusion takes over and in case of conservative noise we expect correlation functions to be described by other exponents. By dimensional argument as in the previous section it is possible to realize that there is another value for the critical exponent, $\chi = -(1 + d + \lambda)/3$, which follows from taking into account higher order non linearities and it will be the correct exponent for the long tail of the correlation functions. In the following, we are going to prove this simple scaling argument with renormalization group methods.

IV.2.2 Long distance regime

In order to calculate the exponents for the long distance regime we begin with Eq. (61) which, as above, is regularised by including the lowest order spatial derivative that is agreement with the model symmetries. After linearisation,

$$\begin{aligned}\partial_t h(s) =& \partial_s^2 h(s) + \int_0^s dy h(y)|s-y|^{-\lambda} + \\ & - \int_0^s dy h(y)|s-y|^{1-\lambda}\left[h(s) - (s-y)\frac{1}{2}\partial_s h(s)\right] + \xi(s,t)\,.\end{aligned} \tag{72}$$

where we used $\int_a^b ds\, f(s) \approx (b-a)(f(a)+f(b))/2$.

Before proceeding with renormalisation, we have to take into account other possible non-linearities. We started by considering the linear order in ϕ and we obtained an equation (Eq. (72)) involving quadratic terms, ϕ^2, due to the expansion of the integral. We must therefore come back to the field theory Eq. (29) and keep quadratic terms as well. The only quadratic term in the field theory is

$$\left(1-\hat{\phi}(s)\right)\left(-\phi(s)\hat{\phi(s)}\right)\left[\int_0^s \mathrm{d}y \frac{\hat{\phi}(s-y)\phi(s-y)}{y^\lambda} e^{-\int_{z=0}^{y} \mathrm{d}z \hat{\phi}(s-z)\phi(s-z)}\right]. \tag{73}$$

After functional minimization, it has an opposite sign, such that both terms cancel out. This is not surprising because the symmetry of the system would not allow a term that breaks space reversal symmetry $s \to -s$. Together, we find

$$\partial_t h(s) = \partial_s^2 h(s) + \int_0^s dy h(y)|s-y|^{-\lambda} + \frac{1}{2}\int_0^s dy h(y)|s-y|^{2-\lambda}\partial_s h(s) + \xi(s,t)\,. \tag{74}$$

Considering interactions between right and left nearest neighbour, the advective terms cancel out. Including the next highest order terms we obtain,

$$\partial_t h(s) = \partial_s^2 h(s) + \int_0^x dy h(y)|s-y|^{-\lambda} + \frac{1}{2}\partial_s^2 h(s)\int_0^s dy h(y)|s-y|^{2-\lambda} + \xi(s)\,. \tag{75}$$

The previous calculation can be generalized to any spatial dimension, extending the previous equation with a spatial coordinate in vectorial form, $\mathbf{s}$. In Fourier space the previous equation is written in a more compact form useful for renormalisation group calculations,

$$G_0(\mathbf{q})^{-1} h(\mathbf{q},\omega) = \xi(\mathbf{q},\omega) - \nu \int_{\mathbf{k}} W(\mathbf{q},\mathbf{k}) h(\mathbf{q},\omega) h(\mathbf{k}-\mathbf{q},\omega)\,, \tag{76}$$

where $h(\mathbf{q},\omega) = \int d\mathbf{s} \int dt h\,(\mathbf{s},t)\, e^{i\mathbf{q}s} e^{i\omega t}$, $G_0^{-1} = \left(i\omega + D_0\mathbf{q}^2 + J|\mathbf{q}|^{-\lambda}\right)$. We reintroduced the dimensional parameters from the adimensional Eq. (76) because we are interested in how they scale under renormalization. In Eq. (76) we defined the vertex, which accounts for the non linear part, as

$$W(\mathbf{q},\mathbf{k}) = \frac{1}{2}\left[\frac{\mathbf{q}(\mathbf{k}-\mathbf{q})}{|\mathbf{k}-\mathbf{q}|^{3-\lambda}} + \frac{(\mathbf{k}-\mathbf{q})\mathbf{q}}{|\mathbf{q}|^{3-\lambda}}\right]\,. \tag{77}$$

In the limit $\mathbf{k} \to 0$ (hydrodynamic limit) it scales as $\mathbf{k}$, which implies non renormalization of the vertex function. The renormalization group (RG) flow

follows,

$$
\begin{aligned}
\partial_l D_0 &= [z - 2 + A_{D_0}]\, D_0\,, \\
\partial_l \nu &= [z + \chi - 2 + (3 - \lambda)]\, \nu\,, \\
\partial_l \Gamma_C &= [z - 2\chi - d - 2 + A_{\Gamma_C}]\, \Gamma_C\,, \\
\partial_l \Gamma_{NC} &= [z - 2\chi - d + A_{\Gamma_{NC}}]\, \Gamma_{NC}\,.
\end{aligned}
\tag{78}
$$

IV.3 One Loop correction

After performing standard renormalization group procedures as outlined in the previous section, with the same diagrams as in Fig. S9, we recover the RG flow for the parameters,

$$
\begin{aligned}
\partial_l D_0 &= \left[z - 2 - \frac{K_d \nu^2}{d D_0^3}\left[(d-2)\,\Gamma_{NC} + (d-3)\,\Gamma_C\right]\right] D_0\,, \\
\partial_l \nu &= [z + \chi - 2 + (3 - \lambda)]\, \nu\,, \\
\partial_l \Gamma_C &= \left[z - 2\chi - d - 2 - \frac{K_d \nu^2}{2 d D_0^3 \Gamma_C}(1+d)(\Gamma_{NC} + \Gamma_C)^2\right] \Gamma_C\,, \\
\partial_l \Gamma_{NC} &= [z - 2\chi - d]\, \Gamma_{NC}\,,
\end{aligned}
\tag{79}
$$

where $K_d = S_d/(2\pi)^d$ and S_d is the area of a d dimensional sphere.

From the non renormalization of the non conserved noise and of the couplings we get the exact exponent identities $\chi = (-1 - d + \lambda)/3$ and $z = (-2 + d + 2\lambda)/3$. In $d = 1$ and for long distances, correlations decay with an exponent $2\chi = -10/9$. By performing the same procedure as for the short tail, we can estimate the lowest order correction given by finite values of local average DNA methylation. In this case correlation functions for the long tail would scale as $|s - s'|^{-(10/9)^{1+\langle m \rangle}}$.

Taken together, we find that the correlation function in sequence space decays in two algebraic regimes,

$$C(s-s') = \begin{cases} |s-s'|^{-\left(\frac{1}{3}\right)^{1+\langle m\rangle}}, & \text{for } |s-s| \ll 1/\langle m\rangle\,, \\ |s-s'|^{-\left(\frac{10}{9}\right)^{1+\langle m\rangle}}, & \text{for } |s-s| \gg 1/\langle m\rangle\,. \end{cases} \tag{80}$$

The cross-over between these regimes stems from an effective exponential cut-off of the long-range interactions. The position of the cross-over scales with the only length scale in the system, the typical distance between neighboring methylated CpGs, $1/m$, and, intuitively, separates a regime dominated by active feedback between DNA methylation and topology and a regime characterised by passive, conservative fluctuations. The numerical prefactor of proportionality between the position of the crossover and $1/\langle m\rangle$ depends on the statistics of distances between neighboring CpGs in base pairs. We determined this prefactor using stochastic simulations of the disordered system representing the actual CpG positions in the mouse genome (see below) and found that the position of the crossover is approximately equal to $350/\langle m\rangle$.

V Derivation of cross-correlation functions

To derive the cross-correlation between DNA methylation we begin with the master equation (12) and introduce a complementary binary vector $\mathbf{a}$, $a_i \in \{0,1\}$, which describes whether a site i is accessible ($a_i = 1$) or not ($a_i = 0$). We then couple this vector to the DNA methylation dynamics

in the simplest form compatible with our model interpretation in physical space. We begin by considering the expectation value of the product $m_i a_j$ and for notational simplicity assume $i < j$,

$$\langle m_i a_j \rangle = P(m_i = 1, a_j = 1) = P(a_j = 1 | m_i = 1) P(m_i = 1) \,. \tag{81}$$

$P(a_j = 1 | m_i = 1)$ cannot be computed directly as it implicitly depends on other values of $\mathbf{a}$ and $\mathbf{m}$. To proceed, we therefore in a first step "integrate in" the random variable describing accessibility at position a_{j-1},

$$\langle m_i a_j \rangle = \sum_{a_{j-1}} P(a_j | a_{j-1}, m_i) P(a_{j-1} | m_i) P(m_i) \,. \tag{82}$$

Applying this step for the second factor we find

$$\langle m_i a_j \rangle = \sum_{a_{j-1}, a_{j-2}} P(a_j = 1 | a_{j-1}, m_i = 1) \tag{83}$$

$$P(a_{j-1} | a_{j-2}, m_i) P(a_{j-2} | m_i = 1) P(m_i = 1) \,. \tag{84}$$

Repeating these steps $|j - i|$ times, for the sake of simplicity writing $P(m_i = 1)$ as $P(m_i)$), we obtain

$$\langle m_i a_j \rangle = \sum_{a_k, i \leq k < j} P(a_j | a_{j-1}, m_i) P(a_{j-1} | a_{j-2}, m_i) \cdot \ldots \cdot P(a_i | m_i) P(m_i) \,. \tag{85}$$

We are still not in a position to give physical expression for the conditional probabilities as they have an unknown dependence on m_i. For simplicity, we for now assume that the mechanical coupling between base pairs is much

stronger than the coupling of DNA methylation marks,

$$\langle m_i m_{i+1} \rangle \ll \langle a_i a_{i+1} \rangle \,. \tag{86}$$

Therefore, $P(a_j|a_{j-1}, m_i) \approx P(a_j|a_{j-1})$ for $i \neq j$. We will elaborate on this assumption in more detail below.

In the model, the probability of binding decays according to Eq. (34). The physical interpretation of this kernel was that it reflects the binding probability of enzymes a site being compacted. Therefore, in our model, the probability that a site is compacted decays with the distance to the nearest methylated site to the power of $-1/3$.Therefore, in order to reflect this kernel, the conditional probability $P(a_j|a_{j-1})$ should decay as $P(a_j|a_{j-1}) \propto a_{j-1}(1 - K_a/k^\lambda)$ (where a_{j-1} plays the role of a delta function). Taken together, we then obtain

$$\langle m_i a_j \rangle = P(m_i) P(a_i|m_i) \prod_{k=1}^{|j-i|} \left(1 - \frac{K_a}{k^\lambda}\right) \,. \tag{87}$$

In our derivation we implicitly assumed that the conditional probabilities $P(a_j|a_{j-1})$ do not explicitly depend on DNA methylation values at positions other than i. In principle, in order to obtain simple expressions for the conditional probabilities we would have had to not only sum over intermediary positions in the accessibility vector, $\{a_k\}$, but also over all positions in the DNA methylation vector $\{m_k\}$, ultimately giving a sum over exponentially weighted paths between m_i and a_j (Fig. S10). In the limit, where the mechanical coupling K_a is much stronger than the coupling between DNA methylation events this sum over exponentials is dominated by the path with the highest contribution of K_a, the orange path in Fig. S10.

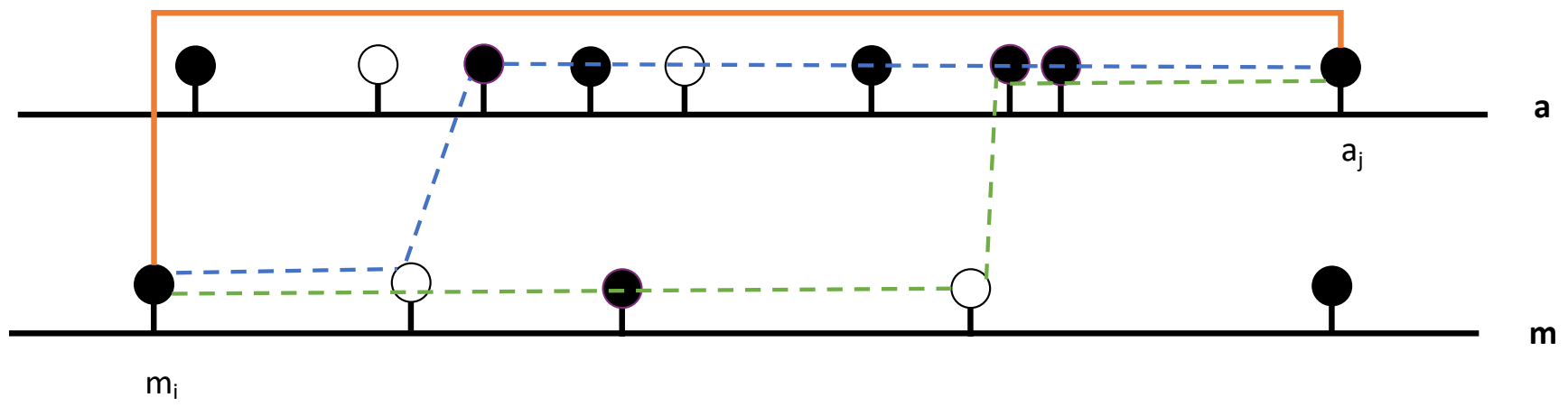

Figure S10: Possible interactions for the epigenetic state in i (m_i in this example) to the one in j (a_j), where m and a are CG and GC methylation respectively. We highlight in orange the dominant contribution to the cross-correlations for the state i. The dashed blue and green line represent different sub-leading contributions.

With $P(m_i) = \langle m_i \rangle$ and defining the local coupling between DNA methylation and accessibility $\alpha = P(a_i|m_i)$ we finally obtain

$$\langle m_i a_j \rangle = \alpha \langle m \rangle \exp\left(-K_a |i-j|^{2/3}\right) . \tag{88}$$

In summary, we find that the strength of cross-correlation is linearly proportional to the average DNA methylation level. The prefactor $\alpha\langle m \rangle$ is thus a measure of the local compaction of the chromatin. Within our approximations this is linear with respect to the DNA methylation. By contrast, the length scale of the decay, $K_a^{-3/2}$, is independent of average DNA methylation. Both results, as well as the functional form of the cross-correlation function, are in good agreement with the experimental data, Fig. 3e of the main text.

Results for different interaction kernels

Relying on field theory and geometric arguments, our procedure can be straight forwardly applied to different classes of interaction kernels. We here

gave detailed calculations for the most difficult scenario involving non-local interactions. The same steps can be applied to general enzyme-DNA kinetics involving short-range interactions (such as processivity or oligomerization) or only local non-linearities (cooperativity). As the corresponding calculations involve subsets of the steps outlined here, we do not present them here and refer for the solutions for the first moment and correlation functions to Table 1 in the main text.

VI Stochastic simulations

To test the validity of our analytical results and their dependence on disorder in CpG positions, we performed extensive stochastic simulations. These simulations were performed by integration of the master equation Eq.(10) using Gillespie's algorithm [13]. For the reasons outlined above, linear and uncorrelated processes do not influence the exponents. Therefore, we do not considered unbinding and demethylation processes. We used dimensionless units where the binding and methylation rates are set to $(J, k_m) = (1, 1)$, respectively. We performed simulations on a one dimensional lattice, where for the distribution of distances between neighboring CpGs was sampled from a distribution resembling the empirical distribution taken from chromosome 1 of the mouse genome. For all simulations, we set the lattice size to 10^6. The simulation of Eq. (55) (Fig. 2F of the main text) is performed with the package xmds2 [14], which performs a pseudospectral integration of the stochastic PDE.

References

1. Olmeda, F. & Rulands, S. Field theory of enzyme-substrate systems with restricted long-range interactions. *Phys. Rev. E* **110,** 024404 (2 2024).

2. Ramaswamy, S., Toner, J. & Prost, J. Nonequilibrium fluctuations, traveling waves, and instabilities in active membranes. *Physical review letters* **84,** 3494 (2000).

3. Hinrichsen, H. Non-equilibrium phase transitions with long-range interactions. *Journal of Statistical Mechanics: Theory and Experiment* **2007,** P07006–P07006 (2007).

4. Rajavelu, A., Jurkowska, R. Z., Fritz, J. & Jeltsch, A. Function and disruption of DNA Methyltransferase 3a cooperative DNA binding and nucleoprotein filament formation. *Nucleic Acids Research* **40,** 569–580 (2011).

5. Täuber, U. C. *Critical Dynamics: A Field Theory Approach to Equilibrium and Non-Equilibrium Scaling Behavior* (Cambridge University Press, 2014).

6. Wijland, F. v. Field theory for reaction-diffusion processes with hard-core particles. *Phys. Rev. E* **63,** 022101 (2 2001).

7. Ginelli, F., Hinrichsen, H., Livi, R., Mukamel, D. & Torcini, A. Contact processes with long range interactions. *Journal of Statistical Mechanics: Theory and Experiment* **2006,** P08008–P08008 (2006).

8. Janssen, H.-K., van Wijland, F., Deloubrière, O. & Täuber, U. C. Pair contact process with diffusion: Failure of master equation field theory. *Phys. Rev. E* **70,** 056114 (5 2004).

9. Cross, M. C. & Hohenberg, P. C. Pattern formation outside of equilibrium. *Rev. Mod. Phys.* **65,** 851–1112 (3 1993).

10. Medina, E., Hwa, T., Kardar, M. & Zhang, Y.-C. Burgers equation with correlated noise: Renormalization-group analysis and applications to directed polymers and interface growth. *Phys. Rev. A* **39,** 3053–3075 (6 1989).

11. Kardar, M., Parisi, G. & Zhang, Y.-C. Dynamic scaling of growing interfaces. *Physical Review Letters* **56,** 889 (1986).

12. Barabási, A. L. & Stanley, H. E. *Fractal Concepts in Surface Growth* (Cambridge University Press, 1995).

13. Gillespie, D. T. A general method for numerically simulating the stochastic time evolution of coupled chemical reactions. *Journal of Computational Physics* **22,** 403–434 (1976).

14. Dennis, G., Hope, J. & Johnsson, M. XMDS2: fast, scalable simulation of coupled stochastic partial differential equations. English. *Computer Physics Communications* **184,** 201–208 (2013).
