# Supplementary material for: Scaling and self-similarity in the formation of the embryonic epigenome
Source: Nat Phys. 2026 Apr 29;22(6):931–40. doi: 10.1038/s41567-026-03263-x (PMC13271883; doi:10.1038/s41567-026-03263-x)
Supplement: Supplementary file 2 — Peer Review File [file 41567_2026_3263_MOESM2_ESM.pdf]

nature portfolio

Peer Review File

Scaling and self-similarity in the formation of the embryonic epigenome

Corresponding Author: Professor Steffen Rulands

Version 0:

Reviewer comments:

Reviewer #1

(Remarks to the Author)
This is an interesting paper that investigates the establishment of DNA methylation features on chromatin using mouse embryonic cells. The authors report that DNA methylation follows a power law with an exponent of 5/2 and exhibits self-similar correlations. Notably, they also observed that changes in methylation precede changes in gene expression. However, the manuscript is difficult to follow, and there are several major concerns that should be addressed to improve clarity and enhance its appeal to the broader scientific community.
Major concerns:
1. The tissue culture section is inadequately presented lacking details of cells, essential quality assurance, or cell identify verification. Moreover, the selection of the in vitro culture model is in adequately described. It remains largely unclear whether and how the selected in vitro culture system may mimic the in vivo developmental process hampering the significance of the work. The rigor and transparency of the current method section does not meet the standard of publication in top tier journals; and must be significantly revised.
2. It is impressive that the de novo methylation trend can be scaled with a power law as shown in Fig. 1d. However, limited details were provided in terms of how each genetic regions were identified. Moreover, it is imperative for the authors to provide a detailed explanation of the scale factor of 5/2 as shown in Fig. 1d. Instead, the paper refers to the 'Methods' section, but the reviewer did not find a discussion that satisfactorily explained the selection of this scaling factor.
3. For the first part of the paper, the authors refer to de novo DNA methyltransferase as DNMT3 (e.g., Fig.2). Instead, DNMT3A and 3B both act as de novo methyltransferase and have distinctive protein-protein interactions. DNMT3A and 3B can both form oligomers (but in distinctive forms) to catalyze DNA methylation. The known catalytic mechanisms of DNMT3a/b are not adequately incorporated or acknowledged in the construction of the physical model presented in the manuscript.
4. The model primarily considers linear DNA distance and demonstrates good predictive power. However, long-range contacts between distal genomic regions play a critical role in chromatin regulation and the establishment of the epigenetic landscape. The authors should at least comment on the potential impact of these distal interactions and discuss whether, and in what way, they may influence the chromatin model.
5. Fig. 4 presents STORM images of nucleus. The staining and antibody used was inadequately presented. WT images should also be included as a comparison. The algorithm used for blob determination and cluster size also need to be clearly presented. Without those, it is not possible to assess the quality or conclusion presented in the super-resolution microscopy section.

Minor concerns:
1. Fig. S2 seems to be mislabeled where b and c was swapped. It remains unclear what is presented in the current UMAP projection. If this is sc-SEQ data, the authors need to provide cell annotations.
2. It will very interesting if the authors highlight specific pluripotent genes in Fig. 5 to enhance the biological significance of their findings.
3. How the findings in this work may shed light on human genome organization should be carefully discussed given the potential different role of DNMTs in mice and human.
In conclusion, while this paper presents significant and novel work, the method lacks sufficient detail and transparency, making it difficult to fully assess the validity of the key conclusions. The limitations of experimental and theoretical model are not adequately discussed, and the statistical analyses of the sequencing data are insufficiently described. In addition, several supporting figures and captions are vague or mislabeled. Overall, the manuscript requires substantial revision to improve clarity and rigor.

Reviewer #2

(Remarks to the Author)
The manuscript submitted by Olmeda et al., describes the de novo DNA methylation dynamics at the early stages of embryonic development and applies the physical space calculation to along with multi-omics experiments and super-resolution microscopy, to address the linear development of 5mC marks on the DNA along with chromatin template. It is very interesting data and calculation that describes the connection of the de novo DNA methyltransferase that could form a phase-separated structure for successful methylation on a linear scale. The manuscript is written well; however, the authors should address the following two key points/provide the supportive experiments that would strengthen their findings.

Major points

Figure 1b needs to be explained well. What is the meaning of the three different colors?? If DNA methylation changes over the time (56h), that needs to be clearly indicated. The current representation is not clear to the readers.

Figure 1d: The authors have integrated H3K4me1, H3K27ac, H3K4me3, and H3K27me3 epigenetic marks with de novo DNA methylation, and they found all marks collapsed into a single curve line. There is no clear information or data available for the ChIP sequencing of the above methyl/acetyl marks. Moreover, it is not explained well how both gene active (H3K27ac/H3K4me3) marks overlap well with gene repressive marks (H3K27me3). The data representation is a bit confusing, and explicit information about how the histone modification data was obtained and used to integrate.

Figure 4b: It's not clear what exactly the authors wish to address using super-resolution microscopy. They used an anti-histone antibody? Which one? H3, H2A, H4, H2B or H1? What are the boxes? How many cells do they count to get the statistically significant values? The authors should provide a detailed description. Also, need to provide statistical information on the number of cells and nuclei counted to draw a conclusion.

Minor points

"De-novo" should be written without the hyphen (De novo)
Figure 1d: What is the unit for the De novo methylation (y-axis)?
Should provide a detailed legend describing all the figures, which would guide the readers, for instance, the legend for figure 4 is not written well with complete information for the readers.

Reviewer #3

(Remarks to the Author)
In this manuscript the authors describe an original and interesting approach to demonstrate that dynamical scaling and self-similarity of DNA methylation marks emerge during embryonic development. The work is original and includes compelling observations that recapitulate previous experimental findings. However, there are several considerations that could help improve the biological significance of the study.

Could the authors provide plausible explanations or hypothesis as to why different methylation densities on regulatory regions of different genes, resulting in a variety of regulatory mechanisms across multiple genes, emerge with self-similarity over time? Would it not instead be expected that genes that are differentially regulated would exhibit differences in their temporal regulation? The conclusions presented are provocative and would benefit from further elaboration, particularly regarding their biological implications for genome function.

At line 106 the authors state "…..the inferred interaction kernel has the biophysical interpretation of the compaction of the DNA around methylated sites and the preferential binding of DNMT3 to compacted regions" is it possible to quantify the level of compaction with this function? It is not clear to me whether the authors are referring to large heterochromatin regions and therefore highly compact genomic regions, or to euchromatin regions. Even if the authors investigate "linear" DNA, in reality the nuclear crowding and epigenetic modifications cause the DNA fibers to adopt very different physical states in active versus repressed regions.

Can the proposed interplay between methylation and local geometric condensates be considered a general phenomenon for any cell type and genomic region? it is hard to envision such a scenario given the high heterogeneity of the chromatin fiber which is organised into clutches. Clutches density varies greatly between cells of different origin and differentiation state. How then can this interplay be regarded as a general mechanism found in many cell types? Upon inhibition of the DMT3 enzyme, is this relation abolished? Do clutches or aggregates fail to form? Can this be experimentally tested? The authors do some interesting experiments toward an effort to validate their hypothesis by performing imaging in cells where Dnmt3a and Dnmt3b were knocked down and reported smaller clusters compared to wild type. However, it remains unclear whether the observed smaller clusters are a direct consequence of the proposed dynamic feedback between DNA methylation and the emergence of scaling and self-similarity in physical space. Could these smaller clusters instead be due to alterations of the overall epigenetic state and cell fate?

In the STORM results, it is unexpected that cluster size is larger in 2i cultured cells than in serum or in ESCs released from 2i culturing condition into serum. Given the increased pluripotent state in 2i, clusters would be expected to be smaller. How

many cells were analysed in these experiments? Which histone antibody was used? What is the variability across passages after release from 2i medium?

At lines 135 – 139, the authors conclude that their simulations reproduce nucleosome clutches as they produce domains of around 5000 bp, that they estimate to measure around 40 nm (in diameter?). Can the authors explicitly develop the conversion from base pairs to physical distance? This is a common complicated problem in the field and would benefit from a clearer explanation.

At line 217 the authors report " We found that for genes which are downregulated between a pair of embryonic stages, these changes in DNA methylation patterns emerge up to two days before changes in the transcriptome appear, suggesting that these marks could play an instructive role by priming the genes for silencing during differentiation"
How is this conclusion validated? Can methylation of these genes be blocked to test whether gene silencing is prevented, thereby reversing the phenotype?

In Fig. 3d, the authors argue that the experimental measurements match the theoretical curves. While this seems to happen at short distances (active feedback region), in all cases shown, it is not so clear in the long-distance regime (passive feedback). This becomes especially evident in the comparisons below (CGI and TSSs). The authors need to mention this point in the text, and if possible, clarify why such behavior is occurring. Could this affect the interpretation that large-scale compaction is also affected by methylation on a general level? Additionally, the authors should make sure that concepts like CGI and TSSs are introduced or at least their full names mentioned in the text.

Several figures lack units in their axes and/or colormaps. For example, de-novo methylation and methylation density in Fig. 1 c,d; Fig. 2 e; and single-cell expression results in Fig. 3, or fig. S2. The authors need to make sure that all figures include such information to ensure clarity.

Some references to sections in the Supplementary text are incorrect, and the document contains several typos. Given its length and the fact that it includes most mathematical derivations essential to understand the mathematical modeling, the authors should carefully revise the clarity and correctness of the text. In the main text, some conclusions drawn from the mathematical descriptions in the Supplementary text are only vaguely referenced (i.e. lines 102 – 103, l2/3 scaling) and are difficult to follow. Such particularly relevant conclusions should be referenced more specifically (for example, by citing specific sections in the supplementary text), and ideally accompanied by a few lines of explanation in the main text, or at least facilitating their interpretation. The mathematical modeling part is pivotal to the work and only discussed briefly in the main text, which makes it difficult to follow, especially for readers that are not familiar with the subject.

Version 1:

Reviewer comments:

Reviewer #1

(Remarks to the Author)
The authors have adequately addressed my previous concerns.

Reviewer #2

(Remarks to the Author)
The revised version of the draft submitted by Olmeda et al. has improved a lot and now it has much more clarity in representation. The study will contribute significant knowledge on the mechanisms of de novo DNA Methylation during development. The authors have addressed all my comments very well. I am fine with the revised draft and publishing an important study in Nature Physics.

Reviewer #3

(Remarks to the Author)
The manuscript has been carefully and thoughtfully revised and it is significantly improved. The authors have carefully address all my comments. Congratulation for the work!

Open Access This Peer Review File is licensed under a Creative Commons Attribution 4.0 International License, which permits use, sharing, adaptation, distribution and reproduction in any medium or format, as long as you give appropriate credit to the original author(s) and the source, provide a link to the Creative Commons license, and indicate if changes were made.
In cases where reviewers are anonymous, credit should be given to 'Anonymous Referee' and the source.
The images or other third party material in this Peer Review File are included in the article's Creative Commons license, unless indicated otherwise in a credit line to the material. If material is not included in the article's Creative Commons license and your intended use is not permitted by statutory regulation or exceeds the permitted use, you will need to obtain permission directly from the copyright holder.
To view a copy of this license, visit https://creativecommons.org/licenses/by/4.0/

Reviewer #1 (Remarks to the Author):

This is an interesting paper that investigates the establishment of DNA methylation features on chromatin using mouse embryonic cells. The authors report that DNA methylation follows a power law with an exponent of 5/2 and exhibits self-similar correlations. Notably, they also observed that changes in methylation precede changes in gene expression. However, the manuscript is difficult to follow, and there are several major concerns that should be addressed to improve clarity and enhance its appeal to the broader scientific community.

We thank the reviewer for the appreciation of our work. We have addressed all issues raised in the revised version of the manuscript. In particular, we have now significantly extended the Methods section and figure captions to provide all methodological details. All changes are marked in red.

Major concerns:

1. The tissue culture section is inadequately presented lacking details of cells, essential quality assurance, or cell identify verification. Moreover, the selection of the in vitro culture model is in adequately described. It remains largely unclear whether and how the selected in vitro culture system may mimic the in vivo developmental process hampering the significance of the work. The rigor and transparency of the current method section does not meet the standard of publication in top tier journals; and must be significantly revised.

We apologize for not having provided sufficient details about the tissue culture. We have updated our manuscript to include full experimental details of the *in vitro* model used, including the identity of the cell line. This can be found in a new section at the beginning of the Methods section. We have also revised other parts of the Methods section, including the description of the STORM microscopy experiment and further details of the theoretical and statistical analysis.

The tissue culture system has been shown to recapitulate key transcriptional and epigenetic changes during early development (Ficz et al., Cell Stem Cell (2013); Habibi et al., Cell Stem Cell (2013), Leitch et al., Nature Structural & Molecular Biology (2013), Marks et al., Cell (2012), Kalkan et al., Development (2017), and others). Specifically, the transition of mouse Embryonic Stem Cells (ESCs) from 2i to serum conditions represents the progression from the naïve pre-implantation Inner Cell Mass (ICM) (~E3.5 – E4.5) to the primed post-implantation epiblast (~E4.5 – E5.5). Both epigenetic and transcriptional dynamics are largely maintained in the *in vitro* experiment (Kalkan et al. (2017)).

In the 2i condition, small molecule inhibitors (inhibitors of MEK and GSK3) maintain the cells in a "naïve ground state" of pluripotency, by Inhibition of Erk1/2 and Gsk3β signaling (Ficz et al. (2013), Habibi et al. (2013), Marks et al. (2012)). Cells uniformly express naïve pluripotency factors like Nanog, Klf4, and Rex1. The DNA has very low levels of methylation, similar to the epigenetic reset seen in the early embryo. Culturing cells in serum (usually with LIF) creates "primed" cells for differentiation, much like the post-implantation epiblast just before gastrulation. Upon release to serum, expression of naïve markers (Rex1, Klf4) drops, while expression of "primed" or "formative" markers (Fgf5, Otx2, Pou3f1) increases. The cells undergo a massive wave of de novo DNA methylation (upregulation of Dnmt3a/b). This mirrors the global genome hypermethylation that occurs in the epiblast shortly after implantation, locking down the genome for lineage commitment. DNMT3A and DNMT3B should be upregulated with the first 12-24hrs post release of 2i, with global methylation levels increasing 24-48hrs. At 56hrs (time point of STORM data) methylation levels should be relatively high, although might not have reached the maximum levels.

In addition to the brief statement we had already in the first paragraph of the Results part, in the revised version of the manuscript we have now included more information about the relation between the *in vitro* system and the corresponding stages in

embryonic development in Methods Section “2i release cell culture”, and we refer to this section in the first paragraph of the Results part.

2. It is impressive that the de novo methylation trend can be scaled with a power law as shown in Fig. 1d. However, limited details were provided in terms of how each genetic regions were identified. Moreover, it is imperative for the authors to provide a detailed explanation of the scale factor of 5/2 as shown in Fig. 1d. Instead, the paper refers to the 'Methods' section, but the reviewer did not find a discussion that satisfactorily explained the selection of this scaling factor.

The genomic regions were based on publicly available Chip-seq experiments. Specifically, they are regions that show statistical enrichment for a given protein or antibody (“peaks”). The different genomic annotations have in previous works been shown to behave differently in terms of the regulation of DNA methylation and gene expression (e.g. active marks like H3K4me3, and repressive marks, like H3K27me3). We used the peaks from the original publications of the data. In the revised version of the manuscript, we now include Table S1 which lists all publicly available data sources used in this manuscript, including their unique accession numbers.

We apologise for not having included a specific explanation of the scale factor in the Methods section. We parametrized each curve as $m = a + bt^{c}$, where a is a base DNA methylation level in 2i conditions and b is the scale factor. Figure 1d then shows $m - a$ on the y-axis and t/b on the x-axis. We have now added a detailed explanation of the estimation of a, b and c to the Methods section and give a brief explanation in the figure caption.

While we find that the exponent 5/2 of the power law is generic and reflects the biophysical mechanism of de novo methylation, the prefactor is region specific. This is analogous to other observations of scaling and self-similarity in physics. In our model, the prefactor captures the binding affinity of DNMT3a/b enzymes to the DNA. Although many regulatory factors influence DNMT3A/B binding, CpG density has been shown to

be a major factor in both of DNMT3A/B binding (Baubec et al., Nature (2015)) and DNA methylation (Angemueller et al., Genome Biol. (2017)). To test whether the prefactor reflects DNMT3A/B binding affinity, we analysed DNMT3A/B Chip-seq data from Baubec et al., Nature (2015) and quantified the fold enrichment of the signal over the background signal from a technical control, grouped by CpG density. We observe a non-monotonic behaviour with a characteristic peak between 2% and 3% CpG density (see left plot below). This is in line with Fig. S2a in Baubec et al., Nature (2015). We related this to the prefactor of the scaling law and found that both follow the same trend (see plots below).

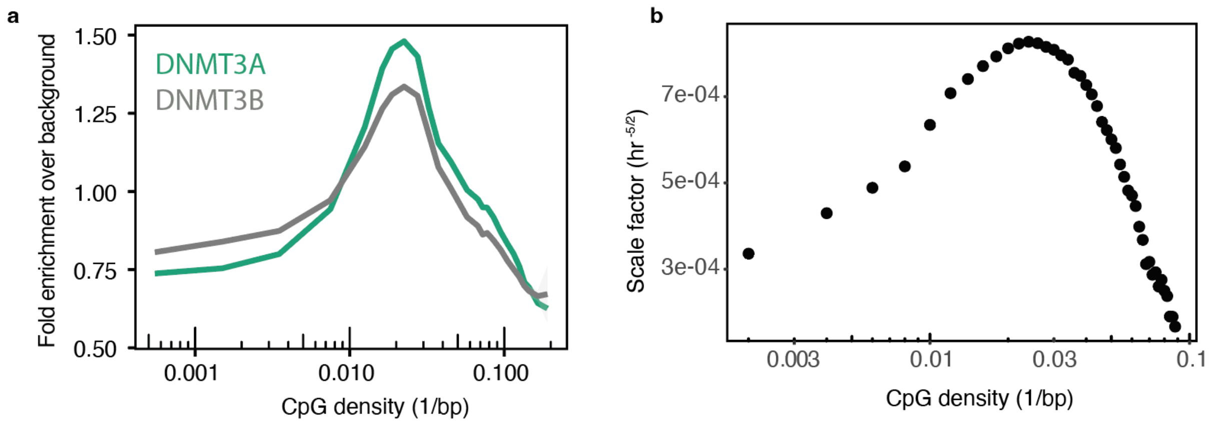

Therefore, as expected, the prefactor reflects the DNMT3A/B binding affinity. The dependence of the methylation rate on the specific genomic position was studied in Ginno et al, Nat. Comm. (2020). We have added a discussion of the interpretation of the scale factor to the second paragraph of the Results part, and added the plots shown above to Supplementary Figure S1.

3. For the first part of the paper, the authors refer to de novo DNA methyltransferase as DNMT3 (e.g., Fig.2). Instead, DNMT3A and 3B both act as de novo methyltransferase and have distinctive protein-protein interactions. DNMT3A and 3B can both form oligomers (but in distinctive forms) to catalyze DNA methylation. The known catalytic mechanisms of DNMT3a/b are not adequately incorporated or acknowledged in the construction of the physical model presented in the manuscript.

We thank the reviewer for highlighting this important point. Our surprising observation is the emergence of self-similarity and scaling *despite* the known differences in the ways in which different regions of the genome are regulated. This applies to DNMT3A and DNMT3B, but also to different histone modifications, or promoters versus gene bodies. The main aim of this work is to give an explanation for this counterintuitive observation.

To directly demonstrate that our observations are independent of the known differences in which DNMT3A/B interact with the DNA, we used Chip-seq binding profiles of both enzymes from Baubec et al., Nature (2015) to identify genomic regions that are either enriched in DNMT3A or in DNMT3B occupancy. We then analysed the whole-genome bisulfite sequencing time course in the same way as shown in Fig. 1D. Both groups of genomic regions show identical behaviour to that shown in Fig. 1D and DNMT3A/B show indistinguishable behaviour of scaling and self-similarity.

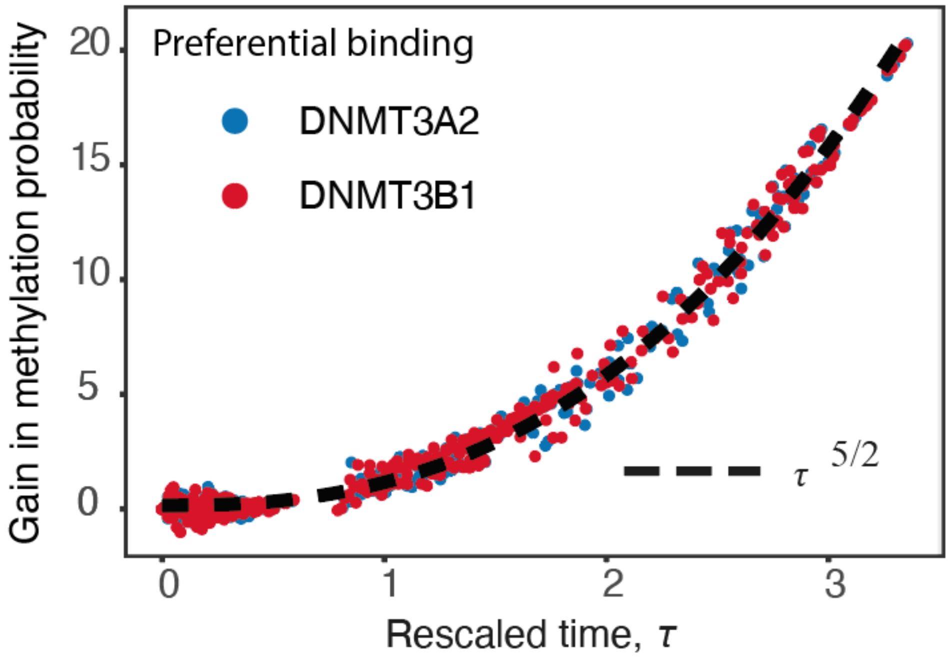

The conceptual reason for why our observations are independent of differences in which genomic regions are regulated (e.g. H3K4me3 and H3K27me3) and differences in DNMT3A/B action is related to the statistical physics concept of universality: systems that follow different rules on the microscopic level can show the same macroscopic behaviour (e.g. a liquid-gas phase transition and a magnetic transition). All systems that

belong to the same universality class are described by the same, often very simple, theory. In the context of our work, our model defines such an attractor theory. Specifically, the feedback between de-novo methylation and chromatin conformation statistically dominates other regulatory processes, such as enzyme processivity. We have added an analytical calculation to the Supplementary Theory Section II.4, in which we show this point mathematically. Our work presents the first observation of this phenomenon in the unexpected context of the molecular regulation of embryonic development.

In the specific case of de-novo DNA methylation, the spatio-temporal behaviour in Figs. 1d and 3d arise because of a positive feedback between de-novo methylation and the local compaction of chromatin. They do not depend on dimensional quantities, like microscopic catalytic details (e.g., DNMT3B being more processive, DNMT3A forming different oligomers, presence/absence of DNMT3L). Such quantities affect dimensional prefactors (amplitudes, onsets, targeting biases, see previous comment) but do not change the emergent scaling form. To demonstrate this explicitly, we took mESC DNMT3A/B Chip-seq data from Bauebec et al., Nature (2015) and partitioned the genome into genomic regions that are either enriched by DNMT3A2 or by DNMT3B1. We then calculated DNA methylation levels as in Fig. 1D separately for DNMT3A2 and DNMT3B1 target regions and by CpG density. Despite the known differences in how both enzymes act, both curves again collapse onto the same scaling form as in Fig. 1d (dashed line).

We also note that, by the same logic, adding DNMT3L explicitly would alter effective recruitment and catalytic rates but is not required to obtain the observed scaling; its effects are captured by the renormalized parameters of the minimal model. Our predictions are even independent of the modes with which DNMT3A/B methylate the DNA (see following paragraph for justification). De novo methylation is therefore an example of one of the core concepts of statistical physics, namely universality.

To also make this point explicit mathematically, we employed renormalization group theory to show that the steric constraints imposed by the feedback between methylation

and compaction dominates all other processes, such as processive methylation of the DNA. In the language of statistical physics, our model defines a universality class that contains potentially many more detailed descriptions that all make the same predictions. We now make this point clear in the main text and added a reference to the specific section in the Supplementary Information supporting this statement.

In the revised version of the manuscript, we

- Added Fig. S1c which reports the analysis using DNMT3A/B Chip-seq data and which demonstrates that the observation of scaling and self-similarity is independent of the specific actions of these enzymes. This further supports the observation of universal scaling behaviour made in Fig. 1d.
- Added a renormalization group calculation to the Supplemental Theory, which analytically and explicitly explains the irrelevance of microscopic details in Figs. 1D and 3d,e. We now also refer to this calculation in the main text, in the last paragraph of Section “From sequence to physical space”
- We now also explain better in the main text why the empirical observations are independent of the molecular details like the differences between DNMT3A/B enzymes or other gene regulatory processes. *“Our theory explains why genomic regions that are regulated in different ways, such as active and repressive genomic regions, follow the same scaling form in Fig. 1d: A renormalization-group argument (Supplementary Information II.4) shows that the feedback between de novo methylation and local chromatin compaction statistically dominate over other processes, such as enzyme processivity (Holz-Schietinger, Journal of Biological Chemistry 2010), cooperativity (Rajavelu, Nucleic acids research 2012), or DNMT3 oligomerisation (Jurkowska Chembiochem 2011). This is analogous to universality classes in critical phenomena.”*

4. The model primarily considers linear DNA distance and demonstrates good predictive power. However, long-range contacts between distal genomic regions play a critical role

in chromatin regulation and the establishment of the epigenetic landscape. The authors should at least comment on the potential impact of these distal interactions and discuss whether, and in what way, they may influence the chromatin model.

The model does already include the interplay between the linear DNA distance and the 3d genome, however only at distances up to roughly 10.000bps. We agree that long-range contacts are important for chromatin regulation and the establishment of the epigenetic landscape. For the phenomenon we found here, scaling and self-similarity during de novo methylation, long-range contacts are, however, of minor importance. The reason for this is that they are statistically rare compared to short-range contacts. Mathematically, the contact probability over long distances decays with an exponent that is larger than the exponents that describe short-range contacts, such that the short-range contacts will dominate the scaling form of time evolution of the average (Fig. 1d) and the correlation functions (Fig. 3). Long-range contacts will, however, influence the shape of the correlation function beyond distances of 10.000bps. These long distances are, however, impossible to resolve with single-cell bisulfite sequencing data as the correlation function there is statistically indistinguishable from 0.

To address this point, we have added the following sentence to the Section ”Validating model predictions”: *“Corrections induced by DNA loops and contacts at distal loci may change the functional form of the correlation functions beyond this length-scale (Lieberman, Science 2009).”*

5. Fig. 4 presents STORM images of nucleus. The staining and antibody used was inadequately presented. WT images should also be included as a comparison. The algorithm used for blob determination and cluster size also need to be clearly presented. Without those, it is not possible to assess the quality or conclusion presented in the super-resolution microscopy section.

We apologize for not reporting these important details in the previous version of the manuscript. To improve the clarity of this part of the manuscript have made the following changes:

1. With the STORM microscopy, we use five cell lines: 2 cell lines where Dnmt3a/b expression was inhibited using knockouts or mutant cell lines (DKO and F2), 2 cell lines representing the corresponding negative controls (cKO and d2A) as well as a wildtype E14 cell line. The images shown in the previous submission was from a conditional knockout cell line, which contains the same genetic elements as the DKO, but where these elements are not activated. It already represented an unperturbed condition. It is important to note that in STORM microscopy, the images shown are computationally generated using Gaussian kernels and do not represent raw data. Further, only a small fraction of nuclei are detected. For this reason, one cannot expect to see "clutches" and differences between conditions in these images. Information about nucleosome clutches comes from the correlations in stochastic localisation events. and rather subtle differences in fluctuations of localisation positions. To not give the false impression that nucleosome clutches are directly discernible in these images we:
    - show an example of an E14 cell (a "proper" wildtype cell line) in the main figure, rather than the cKO cell line shown previously.
    - Added Fig. 4c, which shows pair correlation functions for the cKO and DKO cell lines, and which represent a readout of the STORM signal with minimal statistical processing.
    - Added Figs. S4 and S5, to show examples of cells, localisation signals and pair correlation functions for all analysed conditions and cell lines.
2. We have significantly expanded the corresponding Methods section, including a detailed explanation of cell cultures, staining and antibody used. We have also added a more detailed description of the algorithms used for blob detection and clustering.

Minor concerns:

1. Fig. S2 seems to be mislabeled where b and c was swapped. It remains unclear what is presented in the current UMAP projection. If this is sc-SEQ data, the authors need to provide cell annotations.

Thank you for pointing this out. We corrected the labelling in Fig. S2.

All cells in our single-cell experiments that these UMAPs are based on are mouse embryonic stem cells, which undergo transcriptional and epigenetic changes after the release from 2i to serum conditions. We do not expect qualitatively distinct cell states to emerge within the time frame of this release experiment, such that a comprehensive cell-type annotation might misrepresent the continuous nature of the transition from the naive to the primed state. In Supplemental Figures S2C we provide a visual representation of the expression of a set of relevant genes for all cells.

Nevertheless, the reviewer rightly pointed out a small group of cells that is visually separate from the other cells in the UMAPs and that also clusters separately in our clustering (Cluster D2.2). To investigate what is the biological reason for these cells to cluster separately, we determined their marker genes using the FindMarkers function in Seurat. The following table gives a list of the top 20 genes that are significantly upregulated in cluster D2.2 compared to all other clusters.

p_val_adj	gene
3.43E-21	Gm15772
2.63E-23	Rpl34
1.76E-15	Peg3
1.05E-21	Ppia
1.31E-21	Ftl1
3.38E-23	Rpl29
1.82E-20	Rps27

1.31E-19	Rpl38
9.44E-46	Hprt
2.10E-13	Zfp985
7.18E-22	Hmgn2
4.14E-15	Rps29
3.54E-18	Rpl41
2.52E-22	Rpl23
1.14E-13	Usmg5
2.77E-09	Actg1
2.29E-20	Cbx1
1.82E-16	Uqcr10
6.06E-09	Gja1
1.72E-13	Sugt1

It is notable that there is a number of Ribosomal proteins present, an indicator of cellular stress. A further indication of stress is the presence of Mt1, Ftl1, Sod1, Prdx6 genes, which are indicators of oxidative stress. This view is supported by a gene set

enrichment analysis of all 100 upregulated genes in this cluster.

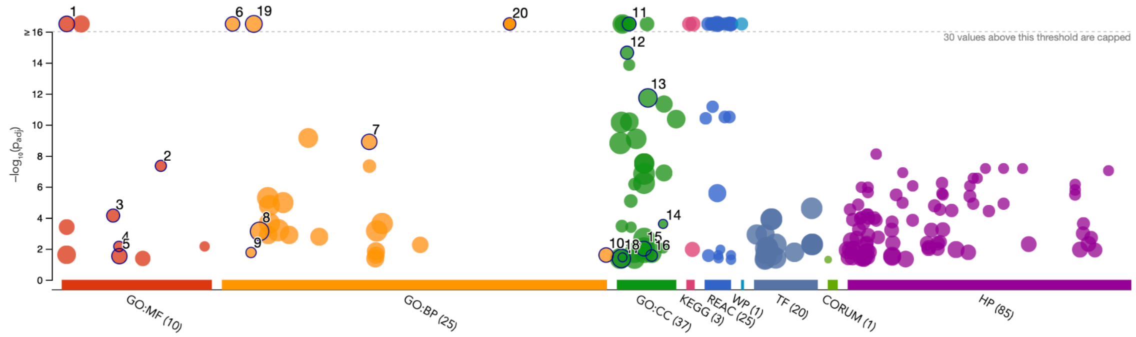

ID	Source	Term ID	Term Name	p_{adj} (query_1)
1	GO:MF	GO:0003735	structural constituent of ribosome	1.582×10^{-24}
2	GO:MF	GO:0048027	mRNA 5'-UTR binding	4.478×10^{-8}
3	GO:MF	GO:0019843	rRNA binding	7.208×10^{-5}
4	GO:MF	GO:0031386	protein tag activity	7.008×10^{-3}
5	GO:MF	GO:0031625	ubiquitin protein ligase binding	2.981×10^{-2}
6	GO:BP	GO:0002181	cytoplasmic translation	1.080×10^{-36}
7	GO:BP	GO:0042254	ribosome biogenesis	1.264×10^{-9}
8	GO:BP	GO:0006915	apoptotic process	7.496×10^{-4}
9	GO:BP	GO:0006122	mitochondrial electron transport, ubiquinol to c...	1.731×10^{-2}
10	GO:BP	GO:2001242	regulation of intrinsic apoptotic signaling path...	2.523×10^{-2}
11	GO:CC	GO:0022626	cytosolic ribosome	3.318×10^{-35}
12	GO:CC	GO:0015935	small ribosomal subunit	2.257×10^{-15}
13	GO:CC	GO:0045202	synapse	1.861×10^{-12}
14	GO:CC	GO:0098556	cytoplasmic side of rough endoplasmic reticul...	2.475×10^{-4}
15	GO:CC	GO:0043209	myelin sheath	9.747×10^{-3}
16	GO:CC	GO:0070069	cytochrome complex	2.729×10^{-2}
17	GO:CC	GO:0005850	eukaryotic translation initiation factor 2 complex	3.566×10^{-2}
18	GO:CC	GO:0005739	mitochondrion	4.220×10^{-2}
19	GO:BP	GO:0006412	translation	2.674×10^{-23}
20	GO:BP	GO:0140242	translation at postsynapse	3.271×10^{-23}

**version**	e113_eg59_p19_f6a03c19
**date**	10/22/2025, 11:44:43 AM
**organism**	mmusculus

g:Profiler

We therefore conclude that the cells in cluster D2.2 are cells that are undergoing cellular stress. Since these cells passed quality control and there is no reason to believe that this stress changes DNA methylation marks on the time scale of this experiment, we did not entirely remove them from our analysis. Indeed, we do not find any deviation in the correlation functions in Fig. 3d. In the revised version of the manuscript, we now discuss this in the legend of Fig. 3b and in more detail in the Methods section.

2. It will very interesting if the authors highlight specific pluripotent genes in Fig. 5 to enhance the biological significance of their findings.

This is an interesting suggestion. For this analysis, we calculated the average correlation function for a set of genes (e.g. pluripotency genes) in the embryo and compared this to the theoretical predictions. We then checked for statistical significance

in the deviation between both quantities. As a rough estimate, the typical length of a gene in mice is 10,000 bps. With 1% CpG density and a typical coverage of 20% this gives, as a rough estimate, 5 covered CpGs per gene. This is insufficient for quantifying any correlations in a single gene, even if one pools all cells. Therefore, while the suggestion is interesting, the statistical power available with current technologies is insufficient to perform this analysis on the level of a single gene. This is why we performed it for gene sets rather than individual genes.

3. How the findings in this work may shed light on human genome organization should be carefully discussed given the potential different role of DNMTs in mice and human.

This is a valid point. While our experiments and theoretical inference are based on mouse embryonic stem cells and the mouse embryo, the fundamental physical mechanism identified here, namely, the feedback between DNA methylation and chromatin compaction, relies on biophysical principles that are conserved across mammals.

Nevertheless, despite having the same domain architecture in mouse and human there are notable species-specific differences between mouse and human DNMT3 enzymes. These are discussed, for example, in Hanna et al., HumanReproduction Update (2018):

- For instance, humans express a broader spectrum of DNMT3B isoforms, including catalytically inactive variants that modulate chromatin binding, whereas in mice DNMT3A and DNMT3B have a more distinct division between germline and embryonic methylation.
- There is also a different role of DNMT3L, which is conserved but regulates different sex-specific promoters in mouse.
- Active demethylation in the paternal genome occurs but is less efficient/complete in humans than in mice, while the maternal genome retains more methylation (~40-50%) than the mouse.

These are all factors that affect the precise localization and kinetic parameters of de-novo methylation. They will therefore be reflected in the scale factors in Fig. 3D (inlay) but not in the emergence of the self-similar scaling form. In Major Point 3 we showed that kinetic differences between DNMT3A/B in mice do not affect the scaling form. In light of this, we have clarified in the revised Discussion that such molecular differences likely modulate the *kinetics* and genomic specificity of methylation establishment, but do not alter the underlying *feedback mechanism* between methylation and chromatin geometry that gives rise to scaling and self-similarity.

To address this point, we have added the following paragraph to the discussion: "The enzymes DNMT3A/B differ in several regulatory aspects between mouse and human, such as in a broader isoform repertoire of human DNMT3B and a stronger role of DNMT3L in mouse compared to. We expect that while such differences, like the differences between DNMT3A and DNMT3B in mouse (Fig. S1c), likely modulate the *kinetics* and genomic specificity of methylation establishment, they are unexpected to alter the underlying *feedback mechanism* between methylation and chromatin geometry that gives rise to scaling and self-similarity."

In conclusion, while this paper presents significant and novel work, the method lacks sufficient detail and transparency, making it difficult to fully assess the validity of the key conclusions. The limitations of experimental and theoretical model are not adequately discussed, and the statistical analyses of the sequencing data are insufficiently described. In addition, several supporting figures and captions are vague or mislabeled. Overall, the manuscript requires substantial revision to improve clarity and rigor.

Reviewer #2 (Remarks to the Author):

The manuscript submitted by Olmeda et al., describes the de novo DNA methylation dynamics at the early stages of embryonic development and applies the physical space calculation to along with multi-omics experiments and super-resolution microscopy, to address the linear development of 5mC marks on the DNA along with chromatin

template. It is very interesting data and calculation that describes the connection of the de novo DNA methyltransferase that could form a phase-separated structure for successful methylation on a linear scale. The manuscript is written well; however, the authors should address the following two key points/provide the supportive experiments that would strengthen their findings.

We thank the reviewer for the positive evaluation of our work. We have addressed all issues raised by the reviewer and think that they have significantly strengthened the manuscript. All changes are marked in red in the revised manuscript.

Major points

Figure 1b needs to be explained well. What is the meaning of the three different colors?? If DNA methylation changes over the time (56h), that needs to be clearly indicated. The current representation is not clear to the readers.

Thank you for highlighting this potential source of confusion. The figure is an illustration of the experimental setup and does not represent actual data. We now have made this point clear in the caption and added a legend to indicate the meaning of different colours. The corresponding empirical figure is Fig. 1c. We now adapted the colours in Fig. 1b such that they match the colours in 1c.

Figure 1d: The authors have integrated H3K4me1, H3K27ac, H3K4me3, and H3K27me3 epigenetic marks with de novo DNA methylation, and they found all marks collapsed into a single curve line. There is no clear information or data available for the ChIP sequencing of the above methyl/acetyl marks. Moreover, it is not explained well how both gene active (H3K27ac/H3K4me3) marks overlap well with gene repressive marks (H3K27me3). The data representation is a bit confusing, and explicit information about how the histone modification data was obtained and used to integrate.

We apologise for not being specific about the origin of these annotations. These annotations are peaks from publicly available Chip-seq data. We have now added a resources table (Table S1) which contains the source for each annotation and other public data used throughout the manuscript. We also now make the processing of the data clear in the figure caption and added a paragraph to the methods section.

Regarding the overlaps between active and repressive marks, we are unsure what the referee is specifically referring to: 1) The overlaps of the respective curves in Fig. 1d, or, 2) the genomic overlaps between these genomic annotations. We address both possibilities below.

1) We thank the reviewer for highlighting this important point. Our surprising observation is the emergence of self-similarity and scaling *despite* the known differences in the ways in which different regions of the genome are regulated. We did not make this point sufficiently clear in the submitted manuscript. The reason why the curves from different genomic regions collapse, despite the known differences in the way in which they are regulated, is related to the statistical physics concept of universality: systems that follow different rules on the microscopic level can show the same macroscopic behaviour (e.g. a liquid-gas phase transition and a magnetic transition). All systems that belong to the same universality class are described by the same, often very simple, theory. In the context of our work, our model defines such an attractor theory. Specifically, the feedback between de-novo methylation and chromatin conformation statistically dominates other regulatory processes, such as enzyme processivity. We have added an analytical calculation to the Supplementary Theory Section II.4, in which we show this point mathematically. Our work presents the first observation of this phenomenon in the unexpected context of the molecular regulation of embryonic development. We have also added a corresponding explanation to the main text in Section “From sequence to physical space”:
“Our theory explains why genomic regions that are regulated in different ways, such as active and repressive genomic regions, follow the same scaling form in

Fig. 1d: A renormalization-group argument (Supplementary Information II.4) shows that the feedback between de novo methylation and local chromatin compaction statistically dominate over other processes, such as enzyme processivity (Holz-Schietinger, Journal of Biological Chemistry 2010), cooperativity (Rajavelu, Nucleic acids research 2012), or DNMT3 oligomerisation (Jurkowska Chembiochem 2011). This is analogous to universality classes in critical phenomena."

2) To quantify the genomic overlaps between the annotations mentioned by the reviewer we performed a co-occurrence analysis using the Jaccard index (Ja) computed on uniformly processed mm10 peak sets (chrM excluded; peaks reduced per mark). The results align with what is expected: enhancer marks co-occur most strongly (H3K27ac–H3K4me1 Ja ≈ 0.28), promoter acetylation and trimethylation show modest overlap (H3K27ac–H3K4me3 Ja ≈ 0.06), and bivalency at promoters is evident but limited (H3K4me3-H3K27me3 Ja ≈ 0.13). Direct co-occurrence between active acetylation and repression is essentially absent (H3K27ac-H3K27me3 Ja ≈ 0.001), with similarly low overlap between H3K4me1 and H3K27me3 (Ja ≈ 0.004) and between H3K4me3 and H3K4me1 (Ja ≈ 0.033). We provide the full Jaccard matrix here:

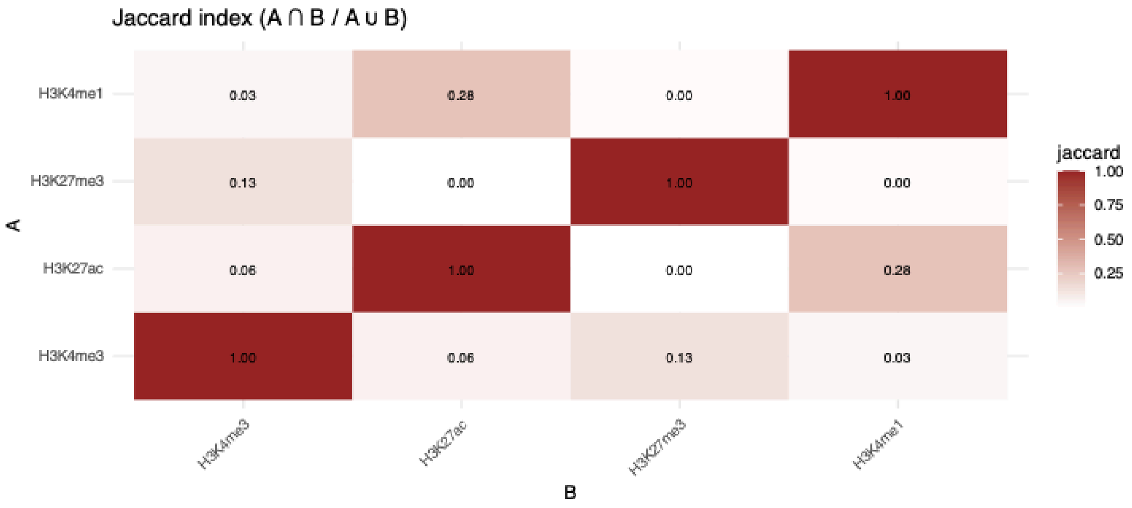

Importantly, the general mechanism we propose, namely an emergent, coarse-grained physical picture that yields a robust 5/2 scaling during de novo methylation, is independent of any specific histone annotation, as explained in the previous point. Histone context primarily renormalizes rates and time scales (cf. inlay of Fig. 1d), not the scaling form itself.

Figure 4b: It's not clear what exactly the authors wish to address using super-resolution microscopy. They used an anti-histone antibody? Which one? H3, H2A, H4, H2B or H1? What are the boxes? How many cells do they count to get the statistically significant values? The authors should provide a detailed description. Also, need to provide statistical information on the number of cells and nuclei counted to draw a conclusion.

We apologize for not explaining the STORM experiment well. Our model makes predictions about the interplay between one-dimensional (epigenetic marks along the DNA sequence) and three-dimensional genome (chromatin conformation). We tested predictions on the 1d genome with single-cell bisulfite sequencing. We tested predictions about the interplay between the 1d and the 3d genome with scNMT-sequencing, sn-m3C sequencing and finally with STORM microscopy. We agree with the reviewer that the STORM experiment is to some degree redundant with the analysis of sc-NMT-seq data in Fig. 3 and sn-m3C data in Fig. 4a. Further, the existence of nanoscale chromatin “clutches” has been reported before. In our work, the STORM experiment serves the following purposes:

- It relies less on bioinformatics and sequencing technologies, making it more easily accessible to the readership of Nature Physics by giving a direct readout of localisations in physical space.
- In contrast to the sn-m3C data, we performed the STORM experiment in the same conditions (2i, release, serum) as in Fig. 3.
- The STORM experiment includes DNMT3 double knockouts. In the release experiment, these constitute the negative control for testing the proposed mechanism behind scaling and self-similarity.

To not bias towards a specific histone we used a pan-histone antibody that targets all five classes of histones. The boxes are examples of regions of interest, in which hotspot localizations were quantified and used for downstream analysis. In the revised version of the manuscript, we have reworked Fig. 4 and the methods section corresponding to STORM microscopy. Specifically, we now provide details on the number of cells and nuclei counted as follows:

Number of cells

	**2i**	**R**	**serum**
cKO	30	25	25
d2A	32	30	26
DKO	29	23	21
E14	26	26	25
F12	27	22	25

Number of ROIs

	**2i**	**R**	**serum**
cKO	326	273	245
d2A	198	242	286
DKO	299	269	181
E14	160	212	253
F12	267	267	221

Number of localisations

	2i	R	serum
cKO	1202757	852883	747523
d2A	762931	872086	638591
DKO	899270	788842	484099
E14	536228	703153	645671
F12	786929	1050692	481086

In Fig. 4d (cluster sizes) we now explicitly show each cell as a dot. The p-values in the previous submission were based on region-of-interest statistics. In the revised version, we now averaged cluster sizes on a cell level and subsequently calculated p-values to better reflect biological variability. As a result, numerical p-values have changed but significances have not.

To compare the variability across biological replicates, we added the following figure to Fig. S5:

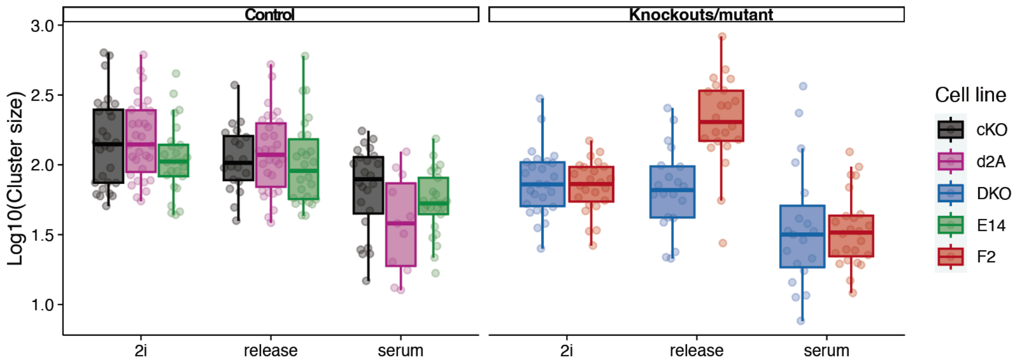

With the exception of DNMT3 inhibited cell line (DKO/F2) in release conditions, results are comparable across cell lines representing the same condition. The difference

between DKO and F2 cell lines likely is a result of the very different genetic mechanisms of inhibition (knockout vs. mutant), and potentially delayed response of the mutant cell line to the change of culture conditions.

In the revised version of the manuscript we now include these numbers and more as a Supplemental Table S2. We have also significantly extended the caption of the revised Fig. 4 and added Figs. S4,5 to better describe the different elements shown in the plots and the cell line used.

Minor points

"De-novo" should be written without the hyphen (De novo)

Thank you for spotting these typos. We have corrected them in the revised version of the manuscript.

Figure 1d: What is the unit for the De novo methylation (y-axis)?

In 2i conditions (time t=0) DNA methylation is not entirely erased. Rather, residual methylation remains in inaccessible regions of the genome (base level). For the y-axis we calculated the fraction of methylated CpGs in a given genomic region, subtracted the base level in that region and then averaged over all regions in a given annotation. Being a difference of fractions or probabilities, the quantity on the y-axis therefore is dimensionless. We changed the axis labels of the main figure and the inlay to make this point clearer.

Should provide a detailed legend describing all the figures, which would guide the readers, for instance, the legend for figure 4 is not written well with complete information for the readers.

We have significantly revised and extended the legends/captions of all figures.

Reviewer #3 (Remarks to the Author):

In this manuscript the authors describe an original and interesting approach to demonstrate that dynamical scaling and self-similarity of DNA methylation marks emerge during embryonic development. The work is original and includes compelling observations that recapitulate previous experimental findings. However, there are several considerations that could help improve the biological significance of the study.

We thank the reviewer for the positive evaluation of our work. We have addressed all issues raised by the reviewer and think that they have significantly strengthened the manuscript. All changes are marked in red in the revised manuscript.

Could the authors provide plausible explanations or hypothesis as to why different methylation densities on regulatory regions of different genes, resulting in a variety of regulatory mechanisms across multiple genes, emerge with self-similarity over time? Would it not instead be expected that genes that are differentially regulated would exhibit differences in their temporal regulation? The conclusions presented are provocative and would benefit from further elaboration, particularly regarding their biological implications for genome function.

This is an insightful question and it directly relates to the heart of what we investigate in this manuscript. Indeed, the reviewer is right, it is expected that the complexity of epigenetic regulation is reflected in the way in which different regions gain DNA methylation over time. We here make the empirical observation that all this complexity can be attributed for by a simple scale factor (scaling behaviour) and that otherwise the gain of DNA methylation follows a universal, self-similar power law. To make this point even clearer, we mESC DNMT3A/B Chip-seq data from Bauebec et al., Nature (2015) and partitioned the genome into genomic regions that are either enriched by DNMT3A2 or by DNMT3B1. Both enzymes target different parts of the genome and have distinct protein-protein interactions. We then calculated DNA methylation levels as in Fig. 1d separately for DNMT3A2 and DNMT3B1 target regions and by CpG density. Despite the

known differences in how both enzymes act, both curves again collapse onto the same scaling form as in Fig. 1d (dashed line).

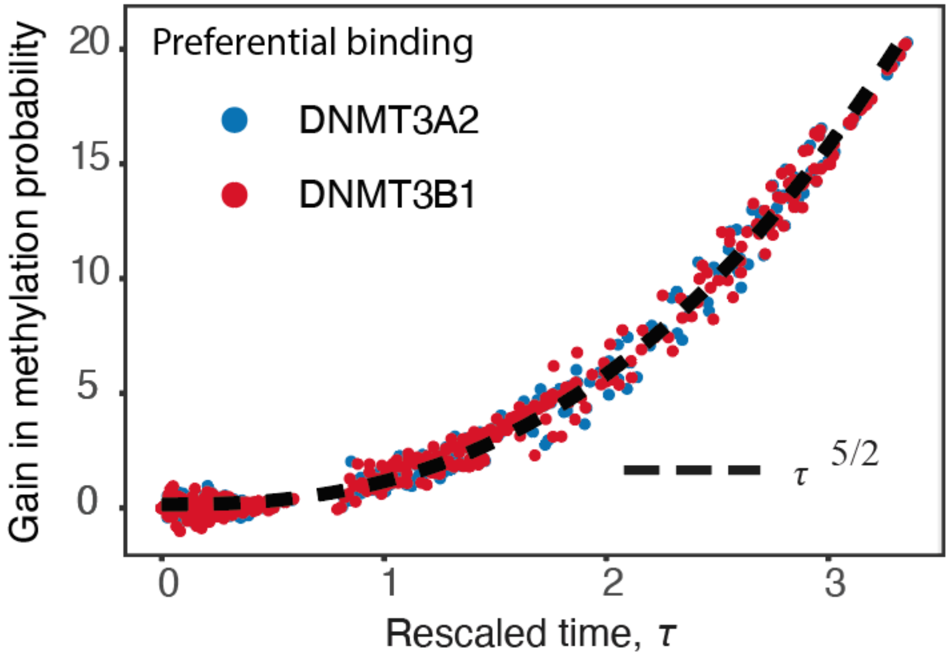

The reason why the curves from different genomic regions collapse, despite the known differences in the way in which they are regulated, is made transparent by our theory: It is related to the statistical physics concept of *universality*: systems that follow different rules on the microscopic level can show the same macroscopic behaviour (e.g. a liquid-gas phase transition and a magnetic transition). All systems that belong to the same universality class are described by the same, often very simple, theory. In the context of our work, our model defines such an attractor theory. Specifically, the feedback between de novo methylation and chromatin conformation statistically dominates other regulatory processes, such as enzyme processivity. We have added an analytical calculation to the Supplementary Theory Section II.4, in which we show this point mathematically. We have also added a corresponding explanation to the main text in Section “From sequence to physical space”:

“Our theory explains why genomic regions that are regulated in different ways, such as active and repressive genomic regions, follow the same scaling form in Fig. 1d: A renormalization-group argument (Supplementary Information II.4) shows that the

feedback between de novo methylation and local chromatin compaction statistically dominate over other processes, such as enzyme processivity (Holz-Schietinger, Journal of Biological Chemistry 2010), cooperativity (Rajavelu, Nucleic acids research 2012), or DNMT3 oligomerisation (Jurkowska Chembiochem 2011). This is analogous to universality classes in critical phenomena."

At line 106 the authors state "…..the inferred interaction kernel has the biophysical interpretation of the compaction of the DNA around methylated sites and the preferential binding of DNMT3 to compacted regions" is it possible to quantify the level of compaction with this function? It is not clear to me whether the authors are referring to large heterochromatin regions and therefore highly compact genomic regions, or to euchromatin regions. Even if the authors investigate "linear" DNA, in reality the nuclear crowding and epigenetic modifications cause the DNA fibers to adopt very different physical states in active versus repressed regions.

In general, the kernel describes co-occurences of de-novo methylation events and it has units 1/time. Therefore, one can only use it to quantify the level of compaction if one has consecutive measurements of DNA methylation in a given genomic region over time. The kernel itself therefore cannot be used to quantify the degree of compaction. However, in Supplemental Theory Section V we derive the theoretical prediction of the cross-correlation between DNA methylation and accessibility, which is based on the interaction kernel. The prefactor of the cross-correlation function shown in Fig. S3a within this framework is a quantification of the degree of compaction. Further, the prediction of the cross-correlation function can be used to estimate chromatin density from DNA methylation measurements. We have now made this clear in the revised version of the Supplemental Theory Sections I and V.

Within the context of large-scale de-novo methylation our results apply to both, large heterochromatin and large euchromatin regions. The density of chromatin affects the prefactor of the scaling form in Fig. 1d, but not the emergence of self-similarity and scaling. This is again a notable observation that results from the statistical physics

concept of universality, as explained above. We have already shown that regions with active (H3K4me3) and repressive (H3K27me3) marks show the same behavior in Fig. 1d. To show explicitly that our results are independent of chromatin compartments, we computed A and B compartments for mESCs from McLaughlin et al., Cell Rep (2019) using the getCompartments function of the HiContacts R library, and calculated average DNA methylation levels as before in Fig. 1d. We again obtain the same scaling form as in Fig. 1d, independent of the chromatin context.

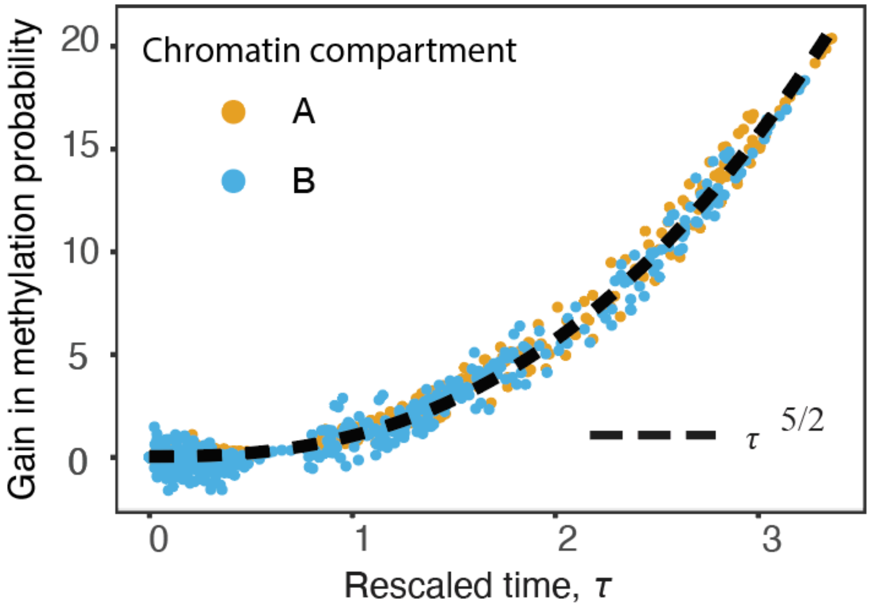

We emphasize that while this is counter intuitive biologically, it is a predicted consequence of the physical mechanism leading to the emergence of scaling and self-similarity. We have added this analysis to Supplemental Figure S1, a description of it to the figure caption and mention this analysis along with the one concerning DNMT3A/B in the main text.

Can the proposed interplay between methylation and local geometric condensates be considered a general phenomenon for any cell type and genomic region? it is hard to envision such a scenario given the high heterogeneity of the chromatin fiber which is organised into clutches. Clutches density varies greatly between cells of different origin and differentiation state. How then can this interplay be regarded as a general mechanism found in many cell types?

We agree that our findings cannot be extended to any cell type. We think that our work applies to scenarios involving large-scale de novo methylation, such as in early embryonic development. It does not apply to scenarios where there is no de novo methylation, such as in fully differentiated cells. De-novo methylation also occurs in other contexts, such as

- Establishment of germline imprints (Smallwood et al., Mol Cell (2011)),
- X chromosome inactivation (Prayer and Lee, Nat Rev Gen (2008)),
- Differentiation and maturation of red blood cells (Challen et al., *Nat Genet* (2012)),

and others. These examples usually comprise only focal de novo methylation, for example of lineage-related genomic regions. Our work applies to these scenarios only when the region undergoing de novo methylation is much larger than the typical size of the predicted condensates. In Fig. 5 we ourselves show that cells “alter” our mechanism to silence pluripotency related genes during priming for differentiation. Given that, our prediction is that the scaling and self-similarity emerge independent of the genomic or chromatin context, as we show in Fig. 1d, Fig. 3, and in the new Figs. S1c and S1d, as well as with our theoretical predictions.

To avoid over-generalization, we have added a discussion of the scope of our findings to the revised Discussion section: *“Our work applies to scenarios involving large-scale de novo methylation. Beyond embryonic development, applications may also include focal de novo methylation processes if the genomic regions being methylated are much larger than the predicted size of the condensates, for example in the establishment of germline imprints, X-chromosome inactivation or the differentiation of red blood cells.”*

Upon inhibition of the DMT3 enzyme, is this relation abolished? Do clutches or aggregates fail to form? Can this be experimentally tested?

The mechanism leading to self-similar scaling in de novo methylation is inherently dynamic. Inhibition of DNMT3A/B largely removes de novo DNA methylation activity.

Inhibition of DNMT3A/B, while still maintaining significant de novo methylation dynamics is not possible.

That said, one can still test the effect of DNMT3 inhibition on the consequences of the de novo methylation process. We have conducted multiple experiments that test the effect of DNMT3A/B inhibition in double knockouts and mutant cell lines. We have tested this in two different ways:

- By analysing genomic correlation functions of DNA methylation marks from scBS-seq experiments, comparing wildtype mouse embryonic stem cells to Dnmt3a/b knockouts in Fig. S3d. We found that correlations in DNA methylation marks are reduced compared to wildtype cells, while the qualitative shape of the correlation function remains similar.
- We compared multiple cell lines, where DNMT3A/B enzymes were inhibited to their negative controls and wildtype cell lines in our STORM microscopy experiment (Figs. 4, S4, S5): 1) Dnmt3a/b double knockouts (DKO) compared to the respective conditional knockouts (cKO), which have the same genetic elements as the double knockouts but where the knockout is not activated; 2) Dnmt3a/b mutant cell lines (F2) compared to the corresponding wildtype cell lines (d2A). We also included the E14 wildtype cell line. These experiments show that upon DNMT3 inhibition clutches are not completely erased, but decrease in size (Fig. 4d) and are more loosely localised (Fig. S5).

Together, these observations show that the presence of DNMT3A/B is not strictly necessary for the existence of condensates. This is in full agreement with previous works (Ricci et al., Cell (2015)). However, in the absence of DNMT3A/B DNA methylation correlations (Fig. S3c) and condensates are reduced (Figs. 4b-d, S5). This implies that the feedback between methylation and chromatin compaction can partially be mediated by DNA methylation itself or the non-catalytic enzyme DNMT3L. Indeed, after DNMT3 inhibition cells retain significant levels of DNA methylationl. This is in agreement with studies that have shown that DNA methylation can lead to tetra-nucleosome compaction *in vitro (Jimenez-Useche, Biophysical Journal 2014)*.

Further investigating the precise molecular role of DNMT3A/B and DNA methylation in this feedback would require cell lines, which have DNMT3A/B but not DNA methylation. This is biologically not feasible, because cells need DNA methylation for genome stability and survival.

That said, the mechanism leading to self-similar scaling is inherently dynamic. Inhibition of DNMT3A/B largely removes de novo DNA methylation activity. Inhibition of DNMT3A/B, while still maintaining de novo methylation dynamics is not possible. The rough similarity of correlation functions and residual condensates in DNMT3A/B knockouts are likely remnants of the de novo methylation process that established DNA methylation in these cells before the knockout was activated and DNA methylation got passively diluted over cell divisions.

The authors do some interesting experiments toward an effort to validate their hypothesis by performing imaging in cells where Dnmt3a and Dnmt3b were knocked down and reported smaller clusters compared to wild type. However, it remains unclear whether the observed smaller clusters are a direct consequence of the proposed dynamic feedback between DNA methylation and the emergence of scaling and self-similarity in physical space. Could these smaller clusters instead be due to alterations of the overall epigenetic state and cell fate?

We thank the reviewer for raising this point. First, we would like to reiterate the point we make in the Discussion, namely that we do not claim that the presence of 5mC directly and alone leads to chromatin compaction. It is well established that factors like MeCP2, HP1 or H3K9 trimethylation interact with DNA methylation and may contribute to the feedback we describe here. While the focus of this manuscript is on understanding the emergence of scaling and self-similarity, we think that disentangling the details of molecular pathways would not contribute to this understanding and be of minor interest to the readers of Nature Physics.

We agree with the reviewer that the Dnmt3 double knockouts could have consequences that in themselves lead to a large-scale reorganization of chromating, such as due to perturbations of other epigenetic factors or cell fate changes. To test this, we analysed single-cell RNA-sequencing data from Dnmt3a/3b double-knockout mouse embryonic stem cells (*Kinoshita,et al PNAS 2021*). The DKO cell lines used in this experiment are the same as we used for the STORM data. We computed differentially expressed genes between WT and DKO using the FindMarkers function in Seurat and performed gene set enrichment analysis using gprofiler:

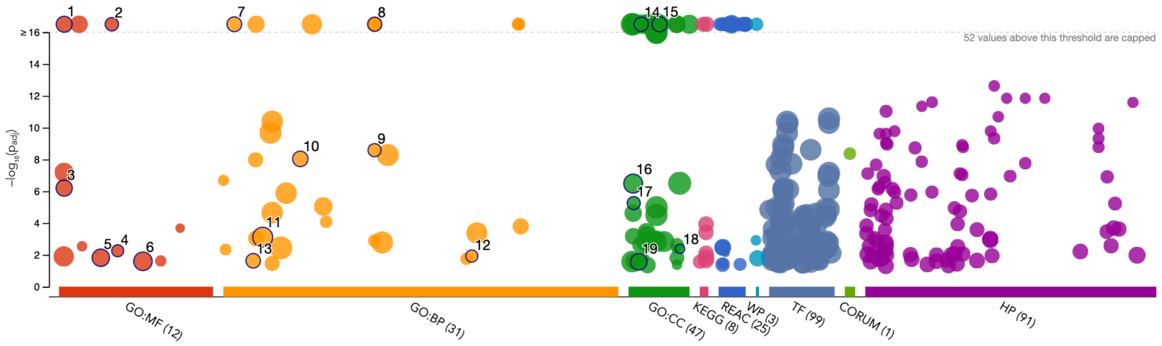

ID	Source	Term ID	Term Name	p_{adj} (query_1)
1	GO:MF	GO:0003735	structural constituent of ribosome	9.110×10^{-58}
2	GO:MF	GO:0019843	rRNA binding	1.209×10^{-17}
3	GO:MF	GO:0003729	mRNA binding	6.332×10^{-7}
4	GO:MF	GO:0031369	translation initiation factor binding	5.526×10^{-3}
5	GO:MF	GO:0016491	oxidoreductase activity	1.517×10^{-2}
6	GO:MF	GO:0044877	protein-containing complex binding	2.531×10^{-2}
7	GO:BP	GO:0002181	cytoplasmic translation	9.044×10^{-78}
8	GO:BP	GO:0042274	ribosomal small subunit biogenesis	2.083×10^{-21}
9	GO:BP	GO:0042255	ribosome assembly	2.619×10^{-9}
10	GO:BP	GO:0016072	rRNA metabolic process	9.217×10^{-9}
11	GO:BP	GO:0006996	organelle organization	7.753×10^{-4}
12	GO:BP	GO:0071353	cellular response to interleukin-4	1.185×10^{-2}
13	GO:BP	GO:0006119	oxidative phosphorylation	2.353×10^{-2}
14	GO:CC	GO:0022626	cytosolic ribosome	7.137×10^{-82}
15	GO:CC	GO:0044391	ribosomal subunit	4.937×10^{-76}
16	GO:CC	GO:0005739	mitochondrion	3.281×10^{-7}
17	GO:CC	GO:0005791	rough endoplasmic reticulum	5.556×10^{-6}
18	GO:CC	GO:0120115	Lsm2-8 complex	4.157×10^{-3}
19	GO:CC	GO:0014069	postsynaptic density	2.749×10^{-2}

**version**	e113_eg59_p19_f6a03c19
**date**	02/12/2025, 08:35:40
**organism**	mmusculus

g:Profiler

There is no enrichment in gene categories related to cell fate or epigenetic remodelling, although the presence of ribosomal and mitochondria-related categories could indicate altered cell stress in this particular experiment. This suggests that *in vitro* Dnmt3a/b

double knockouts do not lead to large scale epigenetic or cell-fate changes beyond the intended effect of the knockouts.

This is fully in line with recent work by some of the co-authors studying the effect of Dnmt3a/b single knockouts *in vivo* (Clark et al., Genome Biol (2022)). We showed that Dnmt3a/b knockouts had little effect on transcriptomes and cell-type proportions at embryonic day E8.5, i.e. shortly after the cell fate decisions that follow on the de-novo DNA methylation process studied in this work.

In the revised version of the manuscript, we now mention these results in the main text in Section “Super-resolution microscopy” and give a more detailed description in the Methods Section “Tissue culture”.

In the STORM results, it is unexpected that cluster size is larger in 2i cultured cells than in serum or in ESCs released from 2i culturing condition into serum. Given the increased pluripotent state in 2i, clusters would be expected to be smaller. How many cells were analysed in these experiments? Which histone antibody was used? What is the variability across passages after release from 2i medium?

It is correct that the larger cluster size in 2i conditions compared to serum conditions is unexpected. This has also been found by Ricci et al., Cell (2015) using a different approach. The larger clusters in 2i conditions inferred with our methods are, however, consistent across all three cell lines and are therefore unlikely to be an experimental artefact. We therefore reasoned that the larger apparent cluster size in 2i conditions must be a result of the statistical nature of the STORM method and the DBSCAN algorithm used for cluster assignment. To test this, we calculated pair-correlation functions of all detected nucleosomes. These pair-correlation functions, shown below, show that nucleosomes are clustered in all conditions (decay of the correlation function).

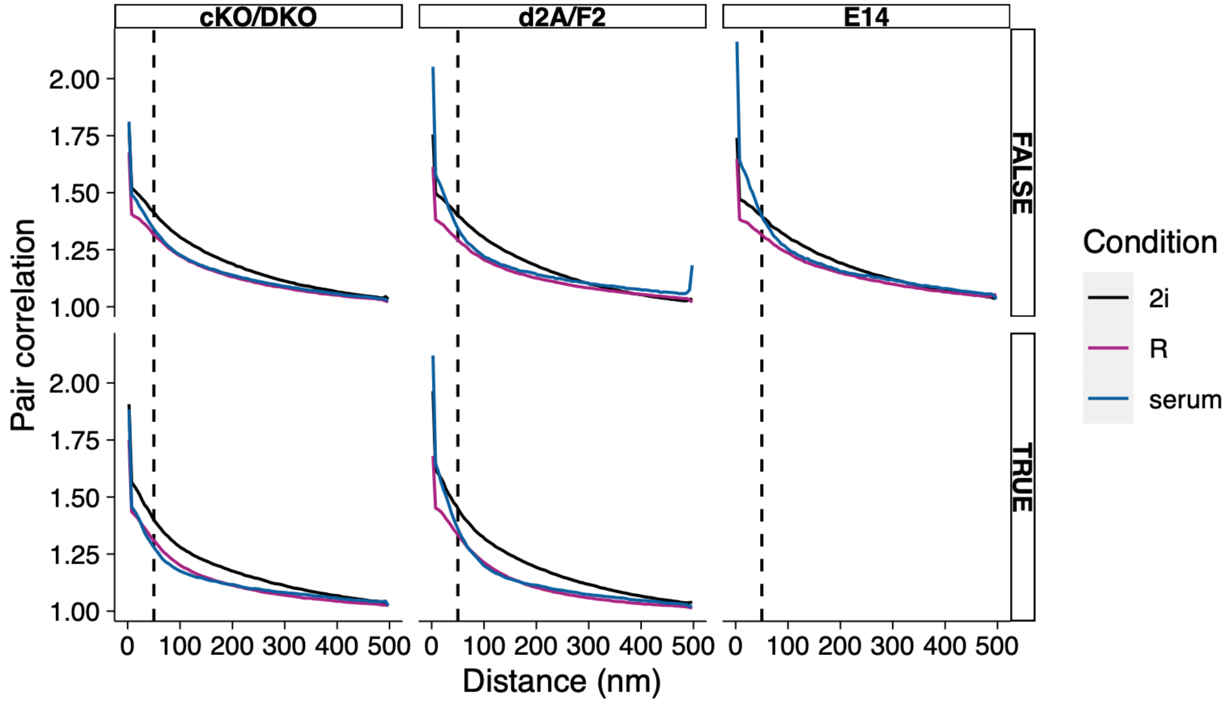

The statistical structure of clusters in both conditions is, however, different: while clusters in serum condition are strongly localized (high correlations at short distances) nucleosomes in 2i conditions are comparatively correlated over longer distances (higher correlations beyond approximately 50nm). Notably, the crossover between both occurs on a similar length scale as predicted by our theory and observed in the sn-m3C experiments (vertical dashed lines).

Therefore, the larger apparent size of clusters in 2i conditions is a result of the DBSCAN algorithm identifying these looser, larger scale correlations as clusters. A possible solution to this problem could be to use specialized clustering algorithms that have higher sensitivity to smaller, strongly localized clusters. This would, however, bias the analysis towards an intended result. We think that the most transparent approach is to use the commonly used DBSCAN algorithm for the detection of clusters, discuss the limitations of the DBSCAN algorithm and report the pair-correlation function analysis in the supplement.

In the revised version of the manuscript we now explain the unexpected differences in the cluster sizes between 2i and serum conditions in the caption of Fig. 4 and report the pair-correlation analysis in Fig. 4c and S5.

The numbers of analysed cells are as follows:

Number of cells

	**2i**	**R**	**serum**
cKO	30	25	25
d2A	32	30	26
DKO	29	23	21
E14	26	26	25
F12	27	22	25

In the revised version of the manuscript we now provide these numbers, along with many other quantities describing the STORM experiment in Supplemental Table S2.

We used a pan-histone antibody to not bias towards any histone. We describe this now in detail in the revised Methods section.

Finally, we assume that by “passages” the reviewer does not refer to passages of the cell lines used in this experiment. All cell lines stem from cell cultures held long-term in different labs and we handle them according to standard protocols. Rather, we assume that the reviewer refers to the variability of our measurements across biological replicates of our experiment, in particular the release from 2i to serum conditions. We have three biological replicates for the unperturbed condition (E14, cKO, d2A) and two replicates for the perturbed condition (DKO, F2) for each of the three studied experiments (2i, release, serum). We emphasise that these are all mouse embryonic stem cells, but that by using cell lines with different genetic backgrounds we obtain a

more realistic estimate of the combination of biological and technical variability than by doing “technical” replicates with repetitions using the same cell line. In particular, our estimate of the biological variability is larger than what would get from technical replicates.

Our estimates of biological variability were already implicit in the boxplots of Fig. 4. In the revised version of the manuscript, we now show them more directly in Fig. S5b.

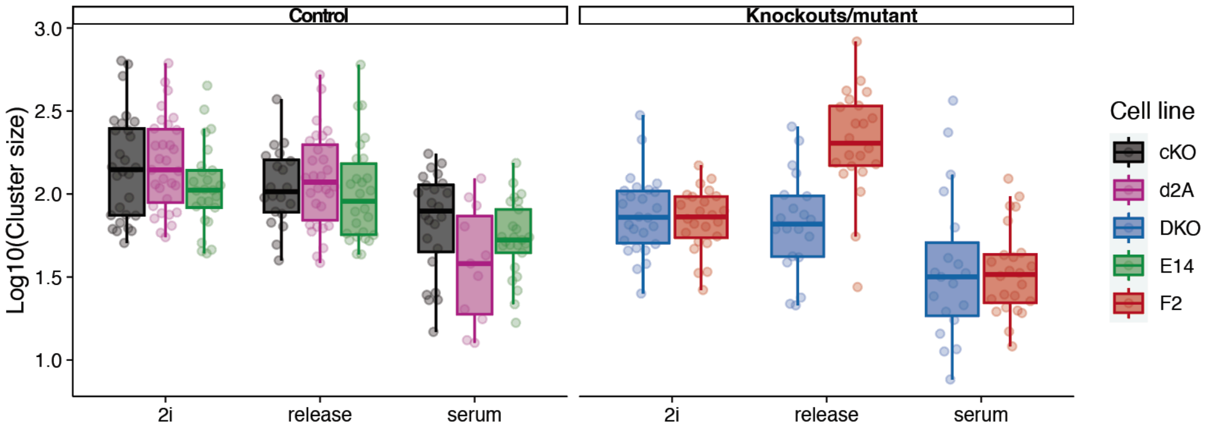

We find general consistency across replicates. Variability of cells (points) reflects the known heterogeneity of embryonic stem cell populations. An exception to this consistency is the different behaviour of the F2 and DKO cell lines in the release experiment. This is not unexpected, because both cell lines have biologically very different means for the inhibition of DNMT3 (knockout vs. mutant). Since the starting (2i) and end (serum) point of the transition are identical we think that the F2 cell line may respond differently or delayed to the change in culture conditions.

At lines 135 – 139, the authors conclude that their simulations reproduce nucleosome clutches as they produce domains of around 5000 bp, that they estimate to measure around 40 nm (in diameter?). Can the authors explicitly develop the conversion from base pairs to physical distance? This is a common complicated problem in the field and would benefit from a clearer explanation.

We are sorry that we were not clearer about this point. The description of this conversion was hidden in the supplement. Apart from geometric considerations we used the random packing fraction to determine a rough estimate of the clutch size. This estimate therefore is a lower bound to the true value.

The reason for why we used a relatively rough estimate here is that we think that other sources of uncertainty outweigh the uncertainty of this step of the analysis, including the heterogeneity of clutch sizes due to stochasticity or the genomic context and the inability to precisely measure the 3d extension of clutches with the methods we have available. We have now added a specific subsection in the Methods part, Section 1.5, where we provide the details of the derivation. We now also make clear in the main text and in the Methods section that the estimate is a lower bound to the predicted typical clutch size.

"To convert sizes of compact chromatin structures with radius r to genomic distances in base pairs, n, and vice versa, we, in a rough approximation, equated the spherical volume in physical space, $4/3\pi r^3$ *and the volume occupied by N nucleosomes,* $4/3\pi r_n^3 N/p_f$*. Here,* $r_n \approx 5.5nm$ *is the radius of a single nucleosome and* $p_f = 0.64$ *is the random packing fraction. By using the packing fraction our result for the predicted diameter of chromatin clutches is a lower bound. Genomic distances were obtained by solving for N and using that the distance across a pair of nucleosomes corresponds to roughly 200bp in sequence space. Genomic and spatial distances were rounded to 1000bp or 10nm, respectively, to reflect that these conversions represent rough estimates."*

At line 217 the authors report " We found that for genes which are downregulated between a pair of embryonic stages, these changes in DNA methylation patterns emerge up to two days before changes in the transcriptome appear, suggesting that these marks could play an instructive role by priming the genes for silencing during differentiation"

How is this conclusion validated? Can methylation of these genes be blocked to test whether gene silencing is prevented, thereby reversing the phenotype?

We agree with the reviewer that these observations would need to be experimentally tested in order to conclude that methylation of these loci is causally associated with gene expression. However, this is beyond the scope of this work in which we report the phenomenon of scaling in the establishment of DNA methylation marks rather than investigate the functional consequences of DNA methylation changes during development. Further, the technical challenges associated with epigenome editing (we do not know of any published work where this has been achieved at a specific embryonic stage *in vivo*) and the possibility of redundant regulatory mechanisms mean that such an undertaking would itself be a separate and extensive body of work. As such we have, instead, toned down our conclusions by adding the following sentence: *"In the future it may be possible to test these observations experimentally via epigenome editing."*

In Fig. 3d, the authors argue that the experimental measurements match the theoretical curves. While this seems to happen at short distances (active feedback region), in all cases shown, it is not so clear in the long-distance regime (passive feedback). This becomes especially evident in the comparisons below (CGI and TSSs). The authors need to mention this point in the text, and if possible, clarify why such behavior is occurring. Could this affect the interpretation that large-scale compaction is also affected by methylation on a general level? Additionally, the authors should make sure that concepts like CGI and TSSs are introduced or at least their full names mentioned in the text.

Our model makes two key predictions in Fig. 3d: first, that there are two regimes of where the correlation function decays with power law exponents -⅓ and -1, respectively. The latter exponent depends weakly on the DNA methylation level. Secondly, it predicts that the position of the crossover between both exponents decreases with the DNA

methylation level. As a result, because the cross-over happens at a large distance, lowly methylated regions (left columns in Fig. 3d) show only the first regime.

In Fig. 3d we compare experimental values with predictions from our analytical theory. The visible deviations between theory and experiment stem from three sources:

- To make the two power-law regimes visible in the plots we must log-transform both the x-axis and the y-axis. This log-transformation can induce a bias in the tails, where the correlation function obtains values close to 0 and, due to noise, negative values. These negative values are undefined after log-transformation, giving a small bias towards larger values of the correlation function when averaging over cells. Note that in the Fig. S2A, which compares analytical predictions and numerical simulations, we see the same deviation between simulations and analytical theory as in some of the panels in Fig. 3d.
- In CGIs and TSSs specifically, correlation for long distances is sparse. Therefore, data points in the tails sometimes are averages over only a few observations. This leads to strong fluctuations in the tails, which are further emphasised by the log-transformation.
- In the model we do not consider genomic systematic variation in the DNA sequence. For example, in CGIs high CpG density regions at small distances are typically followed by lower CpG densities at larger distances in the shores. These changes in CpG density correlate with corresponding systematic changes in DNA methylation. While systematic changes in the average DNA methylation should be taken into account for by the way we calculate (connected) correlation functions, corresponding changes in higher moments of the distribution of $m_i * m_j$ could still lead to systematic deviations.

In the revised version of the manuscript we now explain these differences in the caption of Fig. 3. We have also added an explanation of the terms CGI and TSSs in the caption of Fig. 3.

Several figures lack units in their axes and/or colormaps. For example, de-novo methylation and methylation density in Fig. 1 c,d; Fig. 2 e; and single-cell expression results in Fig. 3, or fig. S2. The authors need to make sure that all figures include such information to ensure clarity.

We apologise for these omissions. Regarding the UMAPS in Fig. 3, we have added axes to all UMAPs. Because the UMAP method is a stochastic algorithm that projects onto a learned manifold, the axes in these plots do not have any particular meaning. To avoid overinterpretation, UMAPS are represented without axes ticks and labels, which is a convention we follow here as well. In the supplement we show ticks and labels. In the revised version of the manuscript, whenever units are not specifically mentioned, like in correlation or cross-correlation functions and probabilities, the plotted quantities are dimensionless. We have gone through all figures and made the following changes:

- We have revised and extended the figure captions of all main figures and supplementary figures 1-5
- We have fixed all dimensions. If no dimensions are given, e.g. for correlation functions or probabilities, plotted quantities are dimensionless.
- We have revised Figs. 1b, 1d, 3c, 4b, 4c, 4d, and 5a.
- We have included one more panel in the main Fig. 4 and two additional supplementary figures.
- Added three panels for Supp Fig. S1

Some references to sections in the Supplementary text are incorrect, and the document contains several typos. Given its length and the fact that it includes most mathematical derivations essential to understand the mathematical modeling, the authors should carefully revise the clarity and correctness of the text. In the main text, some conclusions drawn from the mathematical descriptions in the Supplementary text are only vaguely referenced (i.e. lines 102 – 103, l2/3 scaling) and are difficult to follow. Such particularly relevant conclusions should be referenced more specifically (for example, by citing specific sections in the supplementary text), and ideally accompanied by a few lines of explanation in the main text, or at least facilitating their interpretation.

The mathematical modeling part is pivotal to the work and only discussed briefly in the main text, which makes it difficult to follow, especially for readers that are not familiar with the subject.

We thank the reviewer for carefully reading the supplement. We have carefully revised the Supplemental Theory in the following ways:

- We have corrected many typos and wrong references in the Supplemental Theory. These changes are not marked in red in the revised version of the supplemental theory.
- We agree that the theory is important for deriving the results presented in the main text, and that in its previous form it was not understandable for the broad readership of Nature Physics. The theory makes heavy use of advanced, PhD-level methods from non-equilibrium statistical physics: like second quantisation formalisms, coherent-state path integrals and renormalization group theory applied to non-equilibrium systems. These are tools that are not part of the curriculum in most graduate programs. To convey the reasoning in the Supplemental Theory, we have now added a new section entitled “Overview over the key arguments presented in this supplemental theory”. In this section, we now elaborate on the key logical steps, assumptions and findings presented rigorously in the later parts of the supplemental theory. This section replaces the previous, much shorter “Overview” section, and it should now be understandable for a broad readership.
- In the main text, we have added specific references to the section in the supplemental theory, that a specific result is derived from. Where possible, we now also elaborate more extensively about the reasoning behind a given mathematical result taken from the supplemental theory.

Altogether, the supplemental theory is now more tightly connected to the main text, which should allow a broader readership to follow the interplay between theoretical and experimental reasoning.
